# Renaming the ‘OS-D/CSP’ Family (Part 2): ‘4-Cysteine Soluble Proteins’ (4CSPs)—Intracellular Functions

**DOI:** 10.3390/insects17090940

**Published:** 2026-09-08

**Authors:** Guoxia Liu, Botong Sun, Wei Fan, Shousong Yue, Qiuxia He, Jean-François Picimbon

**Affiliations:** 1 Shandong Academy of Agricultural Sciences, Jinan 250100, China; girlgx@sina.com (G.L.); yueshousong@163.com (S.Y.); 2 Science and Technology Service Platform of Shandong Academy of Sciences, Shandong Academy of Sciences Foreign Students Pioneer Park, Shandong Academy of Sciences, Jinan 250103, China; sunnybotong@163.com (B.S.); fanwei0928@163.com (W.F.); heqx@sdas.org (Q.H.); 3 Shandong Institute for Product Quality Inspection, Jinan 250100, China

**Keywords:** 4CSP (4-Cysteine Soluble Protein), N-4CSP, mucin, actin skeleton regulatory complex, nuclear pore complex protein, transcription initiation factor, cell regulation

## Abstract

The gut, brain, fat body, and various glands, although rich in “CSPs”, are not involved in chemosensory functions. We propose renaming the “CSPs” to “4CSPs” (4-Cysteine Soluble Proteins) to clarify their role, avoiding confusion with proteins also found in wings, hemolymph, and eggs. This report highlights studies related to insecticide resistance and lipid transport and expands on potential intracellular functions, strongly indicating non-chemosensory features of 4CSPs. Additionally, we show a significant correlation between 4CSPs and mucins, translation initiation factors, and actin complex proteins, reinforcing the proposal to rename “CSPs” and consider developmental pattern, tissue distribution, and intracellular localization.

## Simple Summary

Odorant-binding proteins (OBPs) and chemosensory proteins (CSPs) are two distinct groups of small soluble proteins that have been considered significant components of insect olfaction for thirty years. These terms are often used to refer to proteins that carry odor molecules to the sensory receptors; however, the argument becomes chaotic when one considers the tissue expression and development pattern of OBPs and CSPs. We need to look at a particular “CSP”, Mp10, which further challenges the protein family’s “known” function by triggering an immune response when transplanted into plants.

## 1. Introduction

Chemosensory proteins (CSPs) and odorant-binding proteins (OBPs), formerly linked primarily to olfaction, are found in various tissues and life stages of insects and react to insecticide exposure [1,2,3]. This review advocates for revising the conventional view of CSPs and OBPs, particularly the OS-D/A10 Pfam domain, and strongly suggests focusing on their potential intracellular functions rather than their chemosensory roles, providing further evidence against their classification as olfactory proteins [4,5,6,7,8,9].

The intracellular distribution of so-called “chemosensory proteins”, a group that, as we argue in a compelling and reasonable way, is not directly related to chemosensing as it has been customary to believe, is the focus of the review study. By describing what is known about the intracellular distribution of these proteins, we focus on molecular functions while following this group from bacteria to hexapods and examining the wide range of functions carried out by its members. It is now abundantly evident that CSPs are a pleiotropic protein family that serves a variety of purposes in different organisms [10,11,12,13,14]. As such, our claim that a family name reflecting the unique structural elements of the proteins rather than a singular historical function is more than valid.

In Part 1, the focus is on the structure of CSPs, highlighting their cysteine patterns—four conserved cysteines in two distinct disulfide bridges—rather than their vague chemosensory function [1]. The presence of CSPs (and OBPs) in fat body and gut tissues suggests limited chemosensory capabilities [1,2,3,4,5,6,7,8,9]. CSPs are expressed early in development, before significant morphological features like legs and antennae appear [10,11]. The evolution of insect metamorphosis is examined through various species, comparing Holometabola’s life cycle stages to those of hemimetabolous insects. Regardless of morphological variability, “CSPs” are consistently found during dramatic larval transformations leading to a new adult form, which is also true for crustaceans undergoing significant ontogenetic changes [1,12,13,14]. There is no typical CSP structure for chemosensory organs. The sensory tissue protein shares the same structure as gut and developing tissues, characterized by disulfide bridges, a hydrophobic tunnel, and six α-helices, with a molecular weight around 10–12 kDa [1,15,16,17,18]. The ligand-binding site is flexible, accommodating various ligand sizes [19,20]. “CSP” proteins bind to long-chain fatty acids (FAs), such as linoleic acid (LA), which are involved in multi-cascade intracellular processes (i.e., pleiotropy) [21,22,23]. Mp10, a notable protein in this group, retains the structural features of “CSP” but functions in the immune response of plants, suggesting insects may utilize these proteins to bypass plant defenses [24,25]. The example of Mp10 in aphids and the binding of LA by whitefly “CSPs” activated by pesticides provide evidence for the intracellular action of these transporter proteins [21,22,23,24,25]. Part 2 discusses the sequence homology between “CSPs,” “CSP-like proteins”, and many other “bigger” proteins with a range of basic intracellular functions. This strongly advocates renaming this large group of proteins “4CSPs” (“4-Cysteine Soluble Proteins”). Since referring to an intracellular protein as “chemosensory” is a bit ludicrous, we shall use 4CSP throughout the text, the study, and the literature.

Part 1 provides a practically relevant definition of 4CSPs along with a detailed description of their definition and distinguishing characteristics. The Pfam OS-D/A10 (“CSP”) domain may not always match the sequence of a protein that is small, soluble, α-helical, and has at least a few cysteines in places to form disulfide bridges, according to a novel algorithm we present in Part 1 [1]. A 4CSP member is defined by a profile of “4” cysteines and their highly conserved position; not all proteins containing 4 Cys will be members. Then several other proteins, including OBPs, C2H2 transcription factors, cytokines, and RNA polymerases, would not belong here. A formal definition and methodology for including or excluding proteins is the number of cysteines (“4”) and their conserved location (renaming the Pfam Domain OS-D/A10 to 4CSP) [1].

The sequence divergence in “CSPs” from bacteria and insects has been described by Picimbon [14]. Bacterial “CSPs” that show roughly 44–100% identity to insect “CSPs” are similarly subject to renaming [1,14]. Specifically, the genomes of *Kitasatospora* and *Streptomyces* exhibit 44–65% similarity with Diptera 4CSPs. The bacterial/insect 4CSP group is monophyletic, meaning that it is essentially the same protein family (101–124 aa, 11.2–13.6 kDa). However, other proteins (bigger ones with the Pfam “OS-D/CSP” domain in the C-terminus, 457–462 aa, 47.6–48.4 kDa) are inserted into this phylogeny [14]. This is a crucial point because, in this case, this group of proteins should not only be renamed, but also not be referred to as a protein family. This group of bigger 4CSPs could also be proposed concurrently with the idea of renaming (such as “C-4CSP”). A variant of a structure can convergently evolve to exist in multiple unrelated protein families, similar to some conserved folds like barrels, helix-turn-helix, zinc fingers, or something similar.

The role of 4CSPs is reconsidered, proposing their existence within cells due to sequence and structural similarities with various intracellular proteins like PAN-1, mucin, actin skeleton regulatory complex (ASRC), nuclear pore complex protein (NPCP), and transcription initiation factor (TIF) [1,26,27]. The notion of chemosensing is challenged, and the significance of *4CSP* genes in insect physiology and resistance is emphasized. We advocate for renaming “CSP/OS-D” and suggest new roles for “4CSP” in FA lipid transport, intracellular immunological responses, and broader intracellular activities beyond chemosensing, potentially redefining this protein group’s biological function as mainly related to lipoids and metabolic pathways across multiple cells and tissues.

## 2. The Function of 4CSPs in Bacteria: The Earliest Indication of Intracellular Activities

Our study establishes orthology groupings for 4CSP, Thap1, and PAN-1, indicating that these proteins are likely unrelated to chemosensory functions [1,26]. PAN-1 and Bommo-4CSP10 suggest an intracellular role linked to the actin complex or vesicular membranes [1,26,28].

A second clue for intracellular function is the existence of 4CSPs in bacteria [14], which are among the first systems to emerge on Earth [29] and do not have sensillar dendrites [30]. While functional studies in bacteria are needed for conclusive roles, the absence of sensory lymph in prokaryotes strongly supports the idea that 4CSPs serve an intracellular function [14,30]. Bacterial adaptability and resistance to environmental changes relate to their ability to modify intracellular trafficking and lipid distribution [31]. The discovery of *4CSP* genes in various bacteria, including the soil-derived species *Kitasatospora griseola*, supports the view against their chemosensory roles in general [14]. It is not unheard of that a group of proteins with similar structures, or even related ones, can have different functions in different taxa, especially as different as bacteria and insects. For example, there are multiple rhodopsin-related proteins that are totally independent of light, but this does not argue against the function of these proteins in other organisms, nor the other way around [32]. But with 4CSPs, things are a little different. Firstly, unlike opsin, 4CSPs are small soluble proteins with a molecular weight of 10–12 kDa. Second, mutations that change amino acid sequences or folding patterns cause rhodopsins to diverge functionally, moving proteins from ancestral light-driven proton pumps into a variety of roles such as ion channels, sensory receptors, or visual pigments. These modifications affect downstream signaling relationships, spectrum tuning, and tertiary stability [33]. When 4CSPs share the same structural folding, this is not the case. Thirdly, they have been spread by bacteria to insects [14]. Algae and unicellular marine predators are known to acquire a rhodopsin photosystem via a unique lineage of giant viruses (Mimiviridae) [34]. A direct horizontal gene transfer (HGT) of functional *opsins* from bacteria to insects is not supported by any established scientific proof or literature. Insect rhodopsins, which are G protein-coupled receptors required for vision, evolved vertically through the original metazoan lineage rather than being acquired from prokaryotic sources; however, HGT from bacteria and other microorganisms does occur in insects (e.g., for *4CSP* genes) [14,35]. The high degree of identity between bacteria–insect gene pairs (Coleoptera: 64–84%, Diptera: 44–83%, Hemiptera: 74–90%, Hymenoptera: 53–71%, Lepidoptera: 71–100%) indicates that *4CSPs* have only recently been horizontally transferred from bacteria to insects during symbiosis, which is more consistent with a conservation of the original function [14]. In addition to these recent HGTs, there may be some older HGT events that could offer the possibility of additional functional roles of CSP/4CSP proteins developing over evolutionary time. However, these “new” functions may never reach or concern chemosensation because none of the CSP/4CSP proteins found thus far are found to be specific to the chemosensory system [1,2,3,14]. For odorant-related roles developed over evolutionary time, one would expect strict expression in the olfactory organs. Beyond *Kitasatospora*, genera such as *Coccobacillus*, *Staphylococcus*, and *Actinobacteria* from families like Enterobacteriaceae and Pseudonocardiaceae also possess 4CSPs [14]. Additionally, genomic studies reveal *4CSPs* in Firmicutes and other phyla [14]. These bacteria, notable for their roles in the digestive tract and as opportunistic pathogens, are not recognized for their ability to bind plant odors.

Bacterial 4CSPs may be expelled and used in “sensing”, potentially for endosymbionts and/or gut microorganisms, but are unlikely to serve as odorant carriers due to their short signal peptides, which suggests intracellular roles. Secretory proteins must have a signal peptide chain of 16–30 amino acid residues at their N-terminus in order to pass via membranes in both prokaryotic and eukaryotic organisms [36]. But like most insect 4CSPs (see [1] and Figures S1 and S3), the majority of microbial 4CSPs have a signal peptide (SP) that is too short (less than 15 amino acid residues) to cross the membrane [14], meaning they cannot be discharged from the cell. Intracellular roles (cellular homeostasis, cellular scaffolding, and/or signaling pathways) are strongly supported by the size of 4CSP signal peptide [1,14,36]. Our preferred theory posits that 4CSPs are involved in the transport and metabolism of lipoids and nutrients rather than odor transport. While experimental evidence for 4CSPs transporting lipoids is lacking, common molecular and biochemical techniques, such as recombinant protein expression and binding assays, can be applied. Instead of focusing on pheromones or plant odors, researchers should test nutrients or substrates like nitrogen or organic matter. Some bacteria, including specific marine species, can metabolize hydrocarbons and aromatic compounds, suggesting that binding studies for 4CSPs should adhere to a “metabolic paradigm” rather than an “odorant paradigm” [37,38]. The “metabolism” process, which is used by all living cells, enables bacteria and insects to use fat as a source of energy [39,40]. Although glucose is the primary energy source for many bacteria, many can also break down fatty acid and lipid molecules to use them as a significant and viable energy source to sustain diverse functions (that is, pleiotropy) [41,42,43]. 4CSPs may show the intracellular metabolic variety in microbial and insect cells rather than the chemosensory diversity of bacteria and insects [44,45,46]. This effectively addresses the tissue distribution and developmental stages of 4CSPs [1].

The identification of 4CSPs in various motile and nonmotile spore-forming bacteria suggests that this protein family does not function in chemosensation or quorum sensing (QS). While bacteria employ QS with varying chemical signals and mechanisms, there is no difference in 4CSPs between Gram+ and Gram− bacteria [47,48,49,50]. Gram+ bacteria use peptides as auto-inducers (AI), whereas Gram-negative bacteria use compounds like N-acyl homoserine lactone [51,52]. Therefore, the evolution of *4CSP* genes [14] does not align with QS evolution, indicating that 4CSP plays a much broader (metabolic) role predating the divergence of Gram+ and Gram− bacteria.

Interestingly, the 4CSP proteins of *Actinoallomurus* and *Shewanella* range in length from 89 to 129 amino acids (9.9 to 14.9 kDa), but the *Sorangiinae* protein is 482 amino acids long and weighs about 50.7 kDa [14]. In both prokaryotic and eukaryotic cells, the 4CSP region at the C-terminus (“C-4CSP”) may play a crucial function in intracellular FA lipid transport and may be found in even bigger molecular complexes. 4CSPs are thought to engage with AI in the cytosol, TIF, or large transmembrane enzyme receptor protein complexes for bacterial QS and/or substrate degradation. “In insects, 4CSPs are believed to bind to plant odorants at the periphery of ORs rather than functioning intracellularly”. The existence of “C-4CSP” poses a serious challenge to this common wisdom.

## 3. Functional Evidence Supporting the Immune System’s Response, JH, Ecdysone, and the Intracellular Roles of 4CSPs in Insects

Polyclonal antibodies targeting the 4CSP protein were utilized in immunocytochemistry to label the insect antennal sensillum, extending to the cuticle, supporting cells, sensory structures, and intracellular components like the Golgi apparatus and endoplasmic reticulum [53,54]. The distinct labeling of intracellular elements resembles that of OBPs, with lysosomes and vesicles also strongly marked by OBP antibodies within antennal neuronal cells [54].

### 3.1. Intracellular Response to Exposure to Xenobiotics

*4CSP* genes are expressed primarily in the gut and fat body of insects, influencing their response to insecticides and their immune reaction to various pathogens including bacteria and viruses [2,55,56,57,58,59,60]. Therefore, rather than merely responding to pesticides, 4CSPs take part in a very broad innate immunity response. Overexpression of *4CSPs* is noted in response to insecticides, bacteria, viruses, and fungi, indicating their connection to general insect resistance mechanisms. These include “intracellular” lipid pathways enhancing metabolic detoxification, modifying cellular membranes to combat oxidative stress, and building thickened cuticular barriers to slow down toxin penetration [21,22,60,61]. Key processes of insect defense involve lipid mobilization through fat bodies and hydrocarbon synthesis in oenocytes [61].

Insects exhibit similar responses to environmental stressors, including bacteria, insecticides, and viruses, by overexpressing 4CSPs (and OBPs) [3,62,63]. This overexpression aids in resistance to certain insecticides, such as thiamethoxam, in species like *Aphis gossypii* and *Rhopalosiphum padi*, where reducing 4CSP levels increases susceptibility [64,65,66]. However, as demonstrated in whiteflies, 4CSP does not interact with the pesticide chemical thiamethoxam. Instead of attaching to thiamethoxam, the 4CSP protein binds to linoleic acid (C18:2) in the intracellular lipid pathways when thiamethoxam is present [21,22,67]. This introduces 4CSP into intracellular lipid signaling pathways rather than the neonicotinoid’s chemosensation. Increased expression of 4CSPs in many different tissues in response to pesticides further supports their role in pesticide tolerance through activation of intracellular pathways [2]. Furthermore, since the immune system—rather than the olfactory system—is the primary organ that detects bacterial or viral products, it seems improbable that 4CSPs are engaged in chemosensing [68,69,70,71]. Instead of participating in chemosensing at the sensilla level, 4CSPs more likely seem to contribute to pesticide resistance by interacting with cuticular membranes and binding to lipids and/or degradative chemicals of the pesticide.

### 3.2. Intracellular Response to Exposure to Avermectin

Methylated deoxysugar I-oleandrose is a disaccharide in the insecticide avermectin. The 4CSP structure, as a lengthy hydrophobic tunnel, likely binds ‘parts’ of avermectin rather than the entire molecule (C_48_H_72_O_14_). It seems improbable for the 4CSP protein to encapsulate the full macrocyclic lactone due to its structural characteristics [15,21]. Binding of small compounds like benzene and carbohydrates may attract 4CSPs and other protein families, such as OBPs, positioning them near intracellular degradative enzymes instead of near ORs on the dendritic neuronal surface in antennal odor sensilla [2,3,72,73].

Not only is the 4CSP structure too ‘thin’ for C48-chains, but the insecticide’s path through the whole insect body makes a role in relation to avermectin binding impossible. The most likely reason for the expression of 4CSPs in all tissues would be the pesticide moving from the intestines to the adipose body and hemolymph for metabolism [2,74].

This applies to avermectin as well as other insecticides that penetrate the tegument or cuticle but are broken down in the fat body and gut [74,75,76]. It has been suggested that the cuticle contains 4CSP proteins that provide resistance to multiple insecticides [77]. Similar to this, 4CSPs found in the tegument are thought to help the migratory locust tolerate deltamethrin by adhering to the cuticular membrane [78]. This is not a comprehensive explanation for the role of 4CSPs in insect defense because, in locusts and moths, 4CSPs are found in the gut, hemolymph, and fat body both under normal conditions, such as during growth, and in response to dietary changes or diseases [1,2,3,8,21,79,80,81]. Since 4CSPs are expressed in the absence of toxins, they have a fairly broad role in insect physiology from the beginning, most likely in intracellular lipid transport and signaling pathways that are recruited during flight, development, or over stress exposure [2,22].

**Figure 1:**
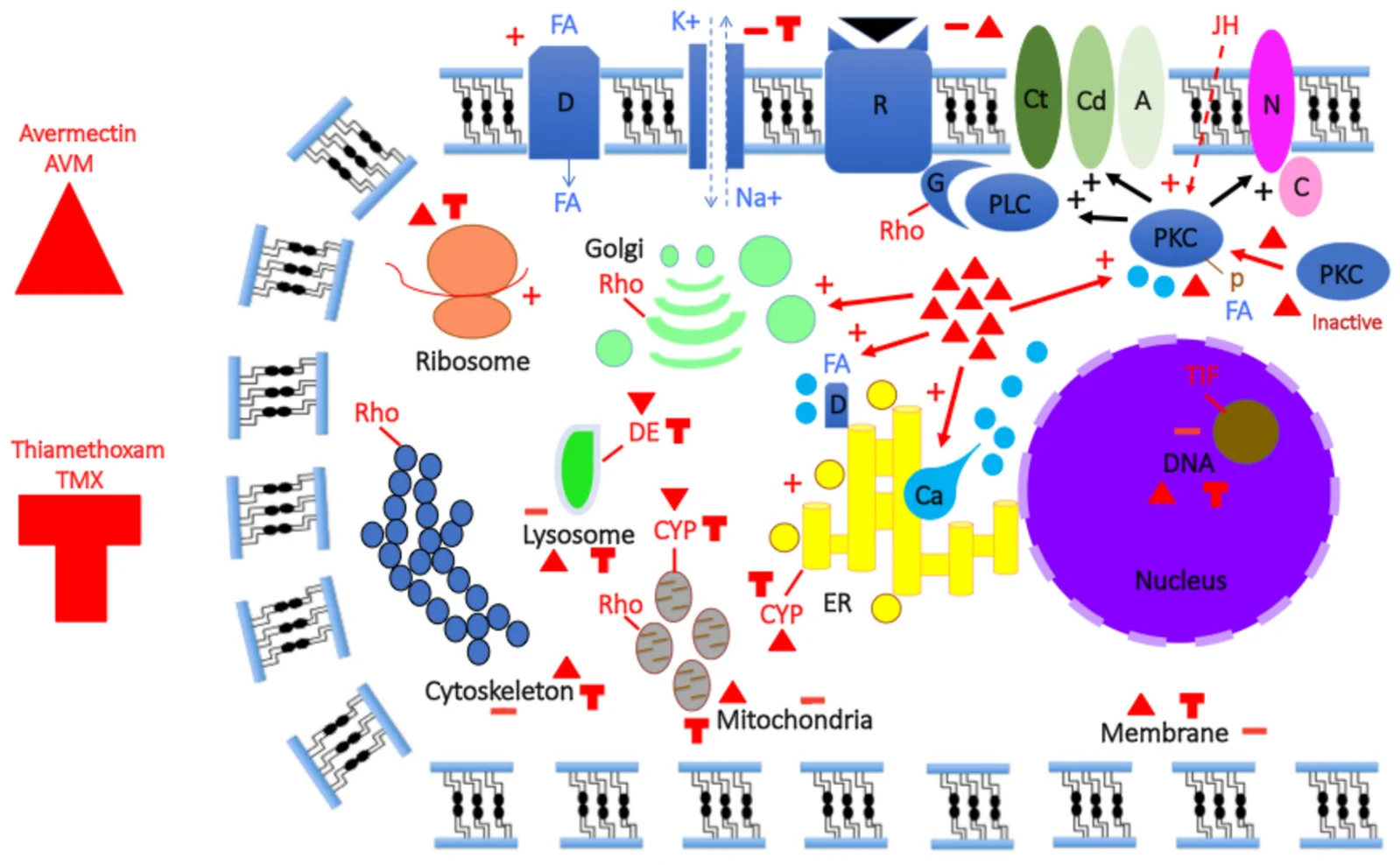
Insect and hexapod cell targets for avermectin and thiamethoxam insecticides. Insecticidal compounds (avermectin: Red triangle; thiamethoxam: Red letter T) enter or pass through the cell membrane via active (transporter-based, endocytosis) or passive (diffusion) pathways. They will seriously harm several organelles inside the cells, including the actin skeleton (expression of Rho), mitochondria (respiratory chain), lysosomes (enzyme inhibition), plasma membrane (fluidity), and nucleus (impaired DNA repair, decreased DNA methylation, point mutations, and transcription deregulation). They will lead to increased lipid uptake, FA accumulation, phosphorylation (p), ribosomal protein expression, ribosome biogenesis, and enlarged vacuoles in the Golgi apparatus. In the ER system, they will cause a prolonged release of calcium from internal stores, which raises the cytoplasmic Ca^2+^ concentration. This significant increase in intracellular concentration of Ca^2+^ ions and lipids is a crucial step in inducing the activation of protein kinase C (PKC), which in turn activates the intercellular junction proteins (cell adhesion molecules; A: Armadillo, C: Coracle, Cd: Cadherin, Ct: Catenin, N: Neurexin) that regulate the insect’s resistance to insecticide penetration. Juvenile Hormone (JH) is involved, and it activates PKC pathways. +: Insecticide-induced reactions that lead to activation of PKC, A, C, Cd, Ct, N, cytochrome P450 (CYP), Degradative Enzyme (DE), and the many enzymes involved in the metabolism of lipids, carbohydrates, and proteins. **−**: Toxic effects. Avermectin (AVM) inhibits muscle contractility and neuronal activity by targeting receptors, ligand-gated ion channels, and glutamate-gated chloride channels (see red triangle). By causing an imbalance between sodium and potassium, neonicotinoid thiamethoxam (TMX) insecticide damages neurons and disrupts normal nerve impulse transmission (see red letter T). D: Desaturase enzyme, K+ intake/Na+ outflow: potassium–sodium exchange pump, R: Receptor, G: G-protein, PLC: phospholipase kinase C, PKC: protein kinase C, Rho: Rho GTPase, ER: endoplasmic reticulum, DE: Degradative Enzyme, TIF: transcription initiation factor.

Another critical issue regarding 4CSPs and intracellular functions is their role in relation to insecticide chemicals and mechanisms of resistance. Insecticides like avermectin can enter cells, damaging the nucleus and activating pathways such as protein kinase C (PKC), thus enhancing resistance by promoting intercellular communication [82,83]. Avermectin triggers different binding proteins in various tissues, leading to the production of 4CSPs and OBPs mainly in the gut and fat body [2,3]. Molecular studies indicate that these proteins, despite their ‘olfactory’ roles, are unrelated to binding pesticides or pesticide metabolites. Notably, while 4CSPs are linked to intracellular fatty acid lipids, general odorant binding protein-2 (GOBP2) and pheromone binding protein-1 (PBP1) have a strong affinity for vitamins [2,3,21,22], which are essential for insect immunity and can influence the development of insecticide resistance [84,85,86]. Additionally, intracellular FA metabolic pathways significantly impact pesticide resistance, as resistant strains often modify their lipid composition to mitigate toxic stress, thereby affecting vulnerability to insecticides [87]. Instead of coming into direct contact with the insecticide, this leads to the 4CSPs being affected intracellularly, along with the intracellular lipid pathways (see Figure 1).

### 3.3. Intracellular Response to Exposure to Thiamethoxam

A study points to a relationship between thiamethoxam-upregulated 4CSPs and FA, indicating that 4CSPs engage in an insecticide response via a lipid pathway instead of direct insecticide contact [21]. This is based on the observation that, while the insecticide thiamethoxam induced *BtabCSP1/Bemta-4CSP1*, Bemta-4CSP1 protein bound to linoleic acid instead of thiamethoxam [21,67]. The mode of action shifted to the transport of intracellular FA lipids after this study introduced 4CSP research into pesticide resistance [22]. This highlights the contrast between the theoretical model of insecticide resistance proposed by Tsouri and Douris [88], which indicates preferential binding of insecticides to 4CSP [89,90,91,92,93,94,95,96], and findings related to whiteflies. The study by G. Liu et al. suggests that Bemta-4CSP1 prefers binding to lipoids over neonicotinoids [21,22]. It emphasizes the importance of using comparative approaches for validating binding data and notes that Bemta-CSP1 is widely produced in various tissues and developmental stages [21], indicating a general role in transporting intracellular lipoids [22]. The PBPs and GOBPs, which are triggered by insecticides but also expressed during insect development as larvae and nymphs as well as during adult reproduction, represent a very similar situation [3]. This finding contradicts the binding of insecticides to PBPs and GOBPs; nonetheless, vitamins, which are crucial for both development and pesticide resistance, are suitable candidates to fulfill the physiological function in these OBPs [3,97]. Pesticides activate different proteins in different tissues, although they mainly affect the intestines instead of the antennae [2,3]. Like other insecticides, thiamethoxam acts by initially interacting with the gut, which controls food absorption and digestion, instead of the antennae. Thiamethoxam molecules can penetrate the intestinal epithelium via endocytic vesicles, transmembrane protein transport, or diffusion across the plasma membrane of epithelial cells [98]. No 4CSP is needed until it reaches the intracellular level (Figure 1).

The role of 4CSPs appears complex, raising important questions about their binding capacity, their expression in the absence of toxins and their structural adaptability in cooperating with insecticide-degrading enzymes [99]. 4CSPs have a major intracellular physiological function in aquatic arthropods, as evidenced by their enhanced expression in response to environmental changes, such as salt and temperature [14]. It also looks into how proctolin affects *4CSP* gene expression in the shrimp heart [14], demonstrating an intracellular function and a broader physiological role for 4CSPs in arthropods [1,14]. Further investigation is needed to fully understand the implications of 4CSPs on insect resistance to toxins and the behavior of bacteria with similar proteins. Even at sublethal doses, glyphosate modifies the expression of genes linked to forager honeybee brains. *4CSPs* (and *OBPs*) are included in this [100,101]. All these changes in 4CSPs in response to environmental changes clearly imply an intracellular function (Figure 1).

### 3.4. Intracellular Response to Exposure to Hormones

4CSPs’ intracellular presence in the corpora allata (CA) and prothoracic glands (PG), key sources of juvenile hormone (JH) and ecdysone in insects, suggests a role beyond olfaction, instead supporting hormone pathways crucial for insect development and resistance to insecticides [1,14,102,103,104,105,106]. These hormones activate intracellular pathways, including PKC and PLC, essential for immune response and lipid metabolism [107,108,109,110,111], and their actions are linked to the regulation of 4CSPs independently of sensory ORs [1,2,3]. The association of 4CSPs with both JH and similar compounds in bacteria proposes a broader functional hypothesis, recommending a reevaluation of their classification away from traditional olfactory proteins [112,113]. When pesticide exposure occurs, JH activates the phospholipase C (PLC) pathway, which includes PKC (Figure 1). Therefore, the insecticide’s impact on 4CSPs, PLC, PKC, and JH biosynthesis strongly implies that, independent of 4CSP binding the insecticide chemical, 4CSPs, various hormonal factors, JH, ecdysone, AKH (adipokinetic hormone), peptide hormones, and lipids, as well as numerous intracellular components like PKC and PLC, can largely cooperate to produce insecticide resistance [2,21,22,107,108,109,110,111,112,113,114,115,116] (see Figure 1).

The presence of 4CSPs in the context of CA indicates that they promote JH intracellular pathways rather than OR activation or other olfactory functions. JH is produced via multiple enzymes [117,118], and its transport may be comparable to the role of OBPs (see [113]). This calls into question terminology—should CSP be referred to as “4CSP” and OBP as “6CSP” instead of chemosensory/olfactory proteins? The existence of 4CSPs in both bacteria and arthropods and their relationship with JH strongly imply potential roles in phurealipids and JH impacts on insect immunity and development, warranting further investigation into their roles in intracellular mechanisms in pertinent species like *Photorhabdus bacilli*. This would be a highly plausible notion until it is shown that 4CSPs are a component of the bacilli’s olfactory hedonics.

## 4. Functional Evidence Supporting the Lipid Transport (LA, C18:2) and Intracellular Roles of 4CSPs in Insects

Evidence indicates that 4CSPs are involved in intracellular lipid transport across various species, including bacteria and arthropods [14]. 4CSPs are present in the lipid sacs of copepods [14]. The widespread presence of 4CSP in multiple species and tissues underlines its diverse functions in intracellular processes. They play roles in insect brain development, molting, immunity, and the regulation of sex pheromone production [1]. Specifically, 4CSP proteins help recognize cuticular lipid hydrocarbons in ants, although their numbers are clearly insufficient for managing the variety of chemical signatures present in other insect species [1,119]. Additionally, studies on whiteflies suggest that 4CSPs participate in pesticide degradation linked to intracellular C18:2-FA pathways [21,22].

C18:2 is integral to all stages of innate immune cell activity beyond energy supply, influencing various intracellular processes such as the omega-6 FA lipid pathway and mitochondrial systems. C18:2-FA lipids significantly impact innate responses through their effects on cell membranes and regulatory functions [120]. For hemocytes, they are vital for energy during critical immune tasks like encapsulation and phagocytosis, demonstrating rapid cytosolic distribution upon infection [121,122], which is highly consistent with the presence of 4CSPs in hemocytes (see [1,14]). Hemocytes depend on 4CSPs and lipids for energy during crucial immunological functions.

A general regulatory role in insect physiology is shown for 4CSPs like Bemta-4CSP1, which is broadly expressed and associated with LA [21,22]. LA has not been detected in insect chemical signatures, raising questions about its function as a contact pheromone. With few species capable of producing LA due to the rarity of Δ12-desaturase [123], it is uncertain if LA/C18:2 could evolve as a specific pheromone. Some insects synthesize pheromones from plant-derived compounds, but this chemical sequestration may impede reproduction, especially among closely evolving plant–insect relationships [124]. Most plant-derived defenses in certain Lepidoptera (Erebidae and Arctiidae) involve pyrrolizidine alkaloids rather than long-chain FAs [124]. Furthermore, the methyl ester component of the FA is often employed as a pheromone instead of the fatty acid itself [125,126]. Thus, doubts persist regarding the pheromonal activation potential of LA.

Food is the primary means of absorbing LA, which, along with food and nutrients, enters the gastrointestinal tract. While some insects use LA as a pheromone precursor [127], its role as a “necromone” suggests the potential for studying ORs related to dead body signals [128]. LA is produced primarily by eusocial insects like aphids and termites, which engage in behaviors like clearing sick or dead bodies [128,129,130]. While ants use “necromone” to find prey [131], aleyrodids do not display any of these behaviors, which makes a compelling case for LA’s intracellular function rather than an OR stimulation [21,22].

## 5. *Myzus persicae* Mp10: Evidence of 4CSP’s Intracellular Activity

The extensive expression of 4CSPs in bacteria and insects, their binding to LA, and their capacity to trigger innate immune pathways when injected into plant phloem are all indicators of their intracellular function [1,21,24,25]. Aphids, particularly the green peach aphid, *Myzus persicae*, are sap-feeding pests that induce plant responses through salivary proteins. The study by Bos et al. [24] and Rodriguez et al. [25] identified about 48 effector candidates, including Mp10 and Mp42, which were analyzed through transient overexpression in the wild tobacco *Nicotiana benthamiana*. Mp10 initiated chlorosis and cell death, stimulated salicylic acid and jasmonic acid pathways, and set off plant defenses. Distinct subcellular localizations for these effectors were observed, suggesting different mechanisms of action. Notably, Mp10 interfered with *Agrobacterium*-mediated overexpression assays, emphasizing its role in plant defense manipulation [24,25].

**Figure 2:**
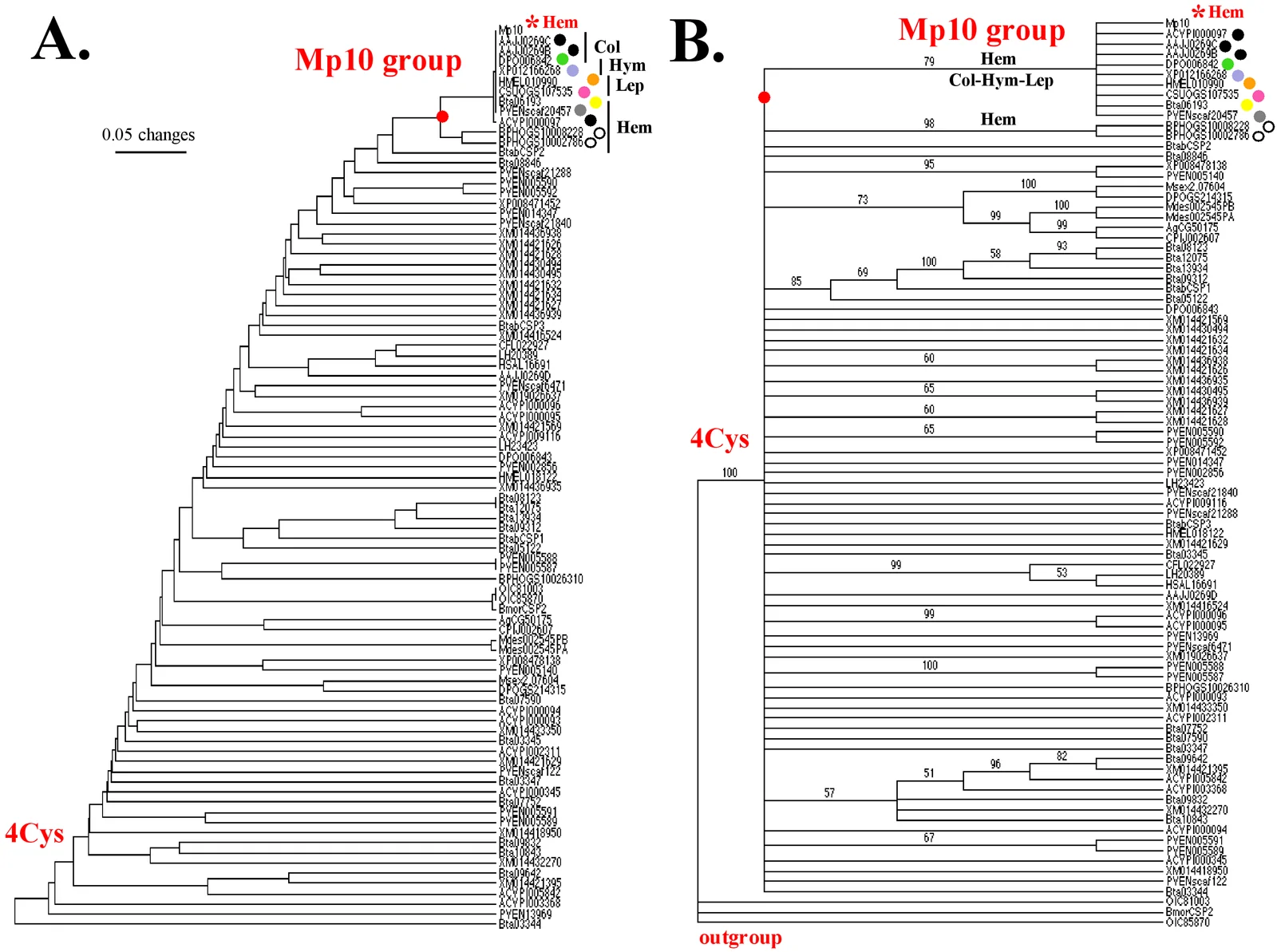
Molecular phylogenetic comparison (PAUP*10Altivec) of Mp10 with related proteins from various insect transcriptomes and genomes from species in the Order Hemiptera (Table S1). (**A**) UPGMA analysis: agglomerative (bottom-up) hierarchical clustering based on the distance matrix of the analyzed taxa that were calculated from a multiple alignment in ClustalX. Evolution (i.e., mutation rate) across all sequences is constant. Each pair-wise distance contributes equally. The red asterisk indicates Mp10’s position (*): Mp10 grouping with AAJJ0269C, AAJJ0269B (in blue), DPO006842 (green), XP_012166268 (purple), HMEL010990 (orange), CSUOGS107535 (pink), Bta06193 (yellow), PVENscaf20457 (gray), ACYPI000097 (black), BPHOGS10008228, and BPHOG10002786 (white) at the top of the tree. (**B**) Bootstrap/Jackknife algorithm analysis with bacterial *A. baumannii* 4CSPs (OIC81003 and OIC85870) as outgroup. Amino acid tree (data matrix: total characters 457, constant characters 48, variable parsimony-uninformative characters 144, parsimony-informative characters 265, all characters of type unord, all characters have equal weight): Length 4832, CI 0.423, RI 0.318, RC 0.134, HI 0.577, G-fit −147.563). AAJJ: Trica, Bta: Bemta, DPO: Denpo, XP_012166268: Bomte, HMEL: Helme, CSUOGS: Chisu, PVEN: Pacve, ACYPI: Acypi, BPHOGS: Nillu. The red dot indicates the distance between the Mp10 group and Nillu sequences. For comparative molecular analysis in the Mp10 family, the protein amino acid sequences are used. InsectBase: All Coleoptera Protein (length 96–132, identity 38–50%, e-value 5.33 × 10^−31^–8.50 × 10^−34^, score 108–118), All Diptera Protein (length 99–101, identity 47–49%, e-value 2.34 × 10^−34^–4.82 × 10^−35^, score 124–127), All Hemiptera Protein (length 94–145, identity 44–97%, e-value 3.54 × 10^−31^–4.45 × 10^−101^, score 109–287), All Hymenoptera Protein (length 97–121, identity 44–49%, e-value 5.19 × 10^−31^–3.38 × 10^−32^, score 111–113), All Lepidoptera Protein (length 109–134, identity 39–43%, e-value 2.08 × 10^−26^–4.93 × 10^−34^, score 98–121). Col: Coleoptera, Hem: Hemiptera, Hym: Hymenoptera, Lep: Lepidoptera. Every protein (taxon) follows the pattern of four cysteines (4Cys; Pfam Domain OS-D/A10).

**Figure 3:**
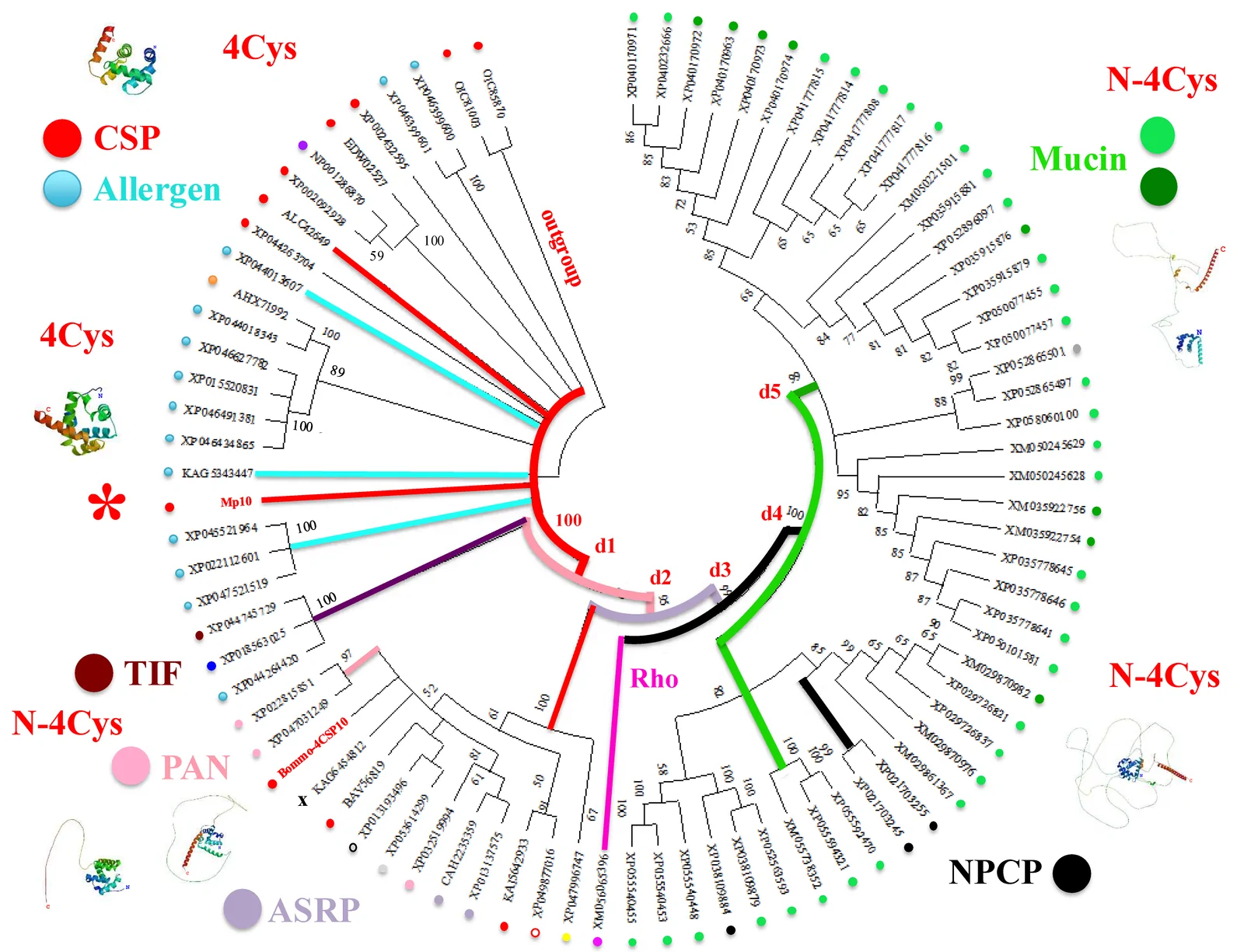
Molecular phylogenetic comparison (PAUP*10Altivec) of Mp10 with related proteins from the protein groups 4CSP, Allergen, TIF, PAN, ASRP, NPCP, and Mucin. Neighbor-Joining (NJ) analysis (sequences from Table S2), phylogeny reconstruction, bootstrap method, 500 bootstrap replications, p-distance, uniform rates. The circular NJ tree and the trees depicted in Figure S4 both read in the same order. The degree of relatedness among amino acid sequences from 4CSPs (Ebsp-3/PebIII, A10/OS-D; red dots), Allergens (Thap1, cyan dots), TIF (brown dots), PAN (salmon dots), ASRP (purple dots), Rho (Rho GTPase-activator, pink dots), NPCP (DAN4, black dots), and Mucin (Mucin-like/Extensin-like, green dots) is indicated by the relative depths of the nodes supported by bootstrap values >50%. Purple: Pherokine-3, orange: Acid trehalase, dark blue: CWA-3, gray: WAS/WASL, light gray: Formin-1, light gray in dark circle: WASP-2, light purple: Jg5928, yellow: RickA-like, white in red circle: YLP motif protein 1, X: Hypothetical protein (unknown function). The bootstrap value for groups of intracellular proteins and 4CSPs is 100 in red. 4CSP (red) branches mix with Allergens, TIF, PAN, ASRP, Rho, NPCP, and Mucin (green) branches. Mp10’s position, with Allergens close to TIF, is shown by the red asterisk (*). d1–d5: sequential gene duplication events that lead to Mucin. The amino acid sequences were used as templates for ClustalX alignments with bacterial *A. baumannii* 4CSPs (OIC81003 and OIC85870) as an outgroup for comparative molecular evolutionary analysis (PAUP*10Altivec). The typical 3D protein structure is depicted for each major protein group. The four-cysteine pattern (4Cys; Pfam Domain OS-D/A10) is followed by all proteins in the 4CSP/Allergen group. The 4Cys pattern appears in the N-terminus of intracellular proteins or taxa in TIF, PAN, ASRC, Rho, NPCP, and Mucin (N-4cys).

Myzpe-Mp10 is a fairly common “4CSP” (153 amino acids, 17.2 kDa, “consensus residues”, four-cysteine patterns, adult whole-body expression, asexual female; see XP_022173691). It builds a phylogenetic tree backed by a highly significant bootstrap value when joined with other 4CSPs and intracellular protein families (see Figure 2 and Figure 3 and Figures S1–S4). Our objective was to provide further information regarding the presence of Mp10/4CSPs in the salivary secretions of the aphid species *M. persicae*, which act as host plant effectors to the aphids’ benefit, in order to begin research investigations into the intracellular function of 4CSPs.

We included species such as *Acyrthosiphon pisum* (Acypi), *Bemisia tabaci* (Bemta), *Halyomorpha halys* (Halha, Brown Marmorated Stink Bug), and *Pachypsylla venusta* (Pacve, Hackberry Petiole Gall Psyllid) in our analysis of the Mp10-4CSP group, using available transcript annotation and genome assembly (see Table S1). We compared Mp10 to 4CSPs from various hemipterans and performed a blastp search against “All Species’s Protein” Database from InsectBase [132], yielding multiple hits (length 101–145, identity 47–97%, e-value 1.80 × 10^−34^–6.35 × 10^−100^, score 126–287) across different species, like *A. pisum*, *Nilaparvata lugens* (Nillu), the brown planthopper, and *Mayetiola destructor*, the Hessian fly. Additional Mp10-hits with varied length, identity, e-value, and score values were recovered by selectively blasting Mp10 in “All Coleoptera Protein”, “All Diptera Protein”, “All Hemiptera Protein”, “All Hymenoptera Protein”, and “All Lepidoptera Protein”, in species such as *A. pisum* (Acypi), *Anopheles gambiae* (Anoga), *Bombus terrestris* (Bomte), *Camponotus floridanus*, *Chilo suppressalis* (Chisu), *Culex pipens* (Culpi), *Danaus plexippus* (Danpl), *Dendroctonus ponderosae* (Denpo), *Diaphorina citri*, *Harpegnathos saltator*, *Heliconius melpomene* (Helme), *Linepithema humile*, *Manduca sexta*, and *Tribolium castaneum* (Trica). However, no matches were found in the transcriptomes of Phasmatodea, Thysanoptera, Odonata, or several other insect taxa (*Archaeopsylla*, *Blattella*, *Catajapyx*, *Chrysopa*, *Ephemera*, *Forticula*, *Limnephilus*, *Locusta*, *Nemurella*, *Mengenilla*, *Pediculus*, *Sialis*, and *Zootermopsis*). A tblastx search corroborated these findings, confirming the specificity of Mp10s.

In our study, a phylogenetic analysis was conducted to examine the Mp10 4CSP-like group within the Hemiptera order, comparing Mp10s from various insect species, particularly focusing on aphids (Table S1 and Figure 2). Using the Unweighted Pair Group Method with Arithmetic mean (UPGMA) method in PAUP*10Altivec [133], the analysis revealed a significant grouping of Mp10s with several 4CSPs from different species, supported by high consensus in protein sequence alignment, particularly in conserved motifs. UPGMA built the initial phylogenetic tree using these primary sequencing data (Figure 2A and Figure S1). The UPGMA tree showed a particular grouping of Mp10 with 4CSPs from Trica (AAJJ0269C and AAJJ0269B, Coleoptera), Denpo (DPO006842, Coleoptera), Bomte (XP_012166268, Hymenoptera), Helme (HMEL010990, Lepidoptera), Chisu (CSUOGS107535, Lepidoptera), Bemta (Bta06193, Hemiptera), Pacve (PVENscaf20457, Hemiptera), Acypi (ACYPI000097, Hemiptera), and Nillu (BPHOGS10008228, and BPHOG10002786, Hemiptera; see Figure 2A). The Mp10 grouping is highly supported by high consensus in the N-terminus and central 88-PDAL-91 motif, as well as conserved amino acid residues like Q, W, L, K-D, and the four Cys characteristic of 4CSPs (Figure S1). We highlight the Mp10-4CSP consensus sequence, important conserved residues in the Mp10s, and the presence of Mp10 “hits” in insect species such as nectar pollinators and plant suckers (Figure S1).

The initial UPGMA tree proposed a hypothesis about the tree structure and Mp10 group identification via sequence alignment (Figure 2A and Figure S1). Bootstrapping confirmed connections among Mp10-related proteins with a 100% bootstrap value (see Figure 2B). The UPGMA and Jackknife trees showed similar topology, indicating a robust Mp10 group (79% bootstrap value), while Nillu sequences were confirmed as distantly related. According to bootstrapping in Figure 2B, the Mp10 group was strong (79% bootstrap), whereas Nillu sequences slid off the Mp10 group, reinforcing their more distant relation to Mp10 compared to pollinators and other insects like Pacve. Further validation by Bootstrap-Jackknife analysis highlighted a strong association between Mp10 and various herbivorous and pollinator insects (Figure 2B). 79% bootstrap supported the grouping of Mp10 with ACYPI000097 (Hem), AAJJ0269C (Col), AAJJ0269B (Col), DPO006842 (Col), XP_012166268 (Hym), HMEL010990 (Lep), CSUOGS107535 (Lep), Bta06193 (Hem), and PVENscaf20457 (Hem; see Figure 2B). This is rather surprising because we would only imagine that Mp10 helps herbivorous insects overcome plant immunity (see [24,25]). Given this, it is noteworthy that Mp10 is more closely related to 4CSPs from Trica, Denpo, Bomte, Helme, and Chisu, than it is to Nillu (see Figure 2, red dot). The presence of Mp10 in aphids, psyllids, whiteflies, and stem borers indicates that it is associated with insects that either burrow or tunnel into plants or consume plant phloem. It is much more surprising that it is also related to the family of insect pollinators like bumblebees and butterflies (see Figure 2A,B and Figure S1).

Notably, the presence of Mp10 in pollinators challenges previously held assumptions about its role, suggesting a potential function in flower dynamics that balances attracting pollinators while deterring herbivores. Moreover, our study calls attention to the implications of pollinator behavior, which may impact plant reproductive success through herbivory and ‘florivory’. Overall, our work highlights the complex ecological interactions between pollinators and blooming plants and includes Mp10.

Examining the role of 4CSPs as flower effectors for pollinators is a recent concept. While evidence shows that Mp10 in aphids aids in controlling plant immunity [24,25], the broader implications for other insects were previously overlooked. Flowering plants face the challenge of attracting pollinators while deterring herbivores, with the risk that frequent pollinator visits could lead to flower damage or reduced nectar. Pollinator interactions can negatively affect fruit and seed development, highlighting that their visits are not always beneficial [134,135]. Molecular ecology indicates that some pollinators, like bees and butterflies, use Mp10s to bypass flower defenses. The evolutionary analysis suggests Mp10 is an ancient mechanism present across various insect taxa, with further research needed to clarify its presence and functional evolution (see Figure 2 and Figure S1).

Our study reveals that the number of Mp10 proteins varies by species, influenced by the host plant or diet (see Figure 2 and Figure S1). Notably, Nillu, which feeds exclusively on rice, exhibits distinct Mp10s compared to other insects (Figure 2). Most Mp10s consist of 147–153 amino acids and show high conservation (97%) in their sequences (Figure S1), indicating a maintained function throughout evolution, particularly in targeting host-plant immune systems in the Aphididae family. Additionally, the split of Acypi and Myzpe approximately 22 million years ago shows stability in Mp10. Future research on Mp10-4CSP in pollinators may enhance understanding of plant defense mechanisms, supporting angiosperm evolution and environmental preservation. Mp10 mechanisms may have emerged in the early Permian era (~298 Mya) at the same time as angiosperms, pollinivory, and the first plant–insect interactions (Figure 2 and Figure S1, [136]).

## 6. Mp10 and Multiple Intracellular Functions from Nucleus to Actin

Interestingly, **Mp10** shares significant similarities with various protein families, such as TIFs, **neural Wiskott–Aldrich Syndrome (WAS/WASL)-like** proteins, **ASRPs**, **NPCPs**, Mucins, **PAN**, **Rho GTPase (Rho)** activators, **serine/threonine-protein kinase C (SamkC)**, and numerous viral **proteins**, revealing a 31–45% identity range (Figure 3 and Figures S2–S4 and Tables S2 and S3). This raises interesting questions regarding the potential functions of 4CSPs in cellular processes, including interactions with molecular components, RNA/DNA binding, transcription control, and the activation of protein complexes, even though homologous function is not always implied by sequence homology.

The experimental role of 4CSP in the actin system and nucleus remains unproven, despite significant sequence homology with proteins related to actin, mucin, and nuclear pores (our study). Phylogenetic and sequence analyses suggest clustering of intracellular protein families with 4CSPs (Figure 3 and Figures S2–S4 and Tables S2 and S3). Functional analyses, including RNAi knockdowns, highlight 4CSP’s involvement in lipid transport, phosphorylation, and immune response, steering attention away from olfactory functions [21,22,137]. Furthermore, glucose-related protein 94 (GRP94), a critical chaperone, assists in 4CSP’s trafficking from the ER to the Golgi, which is vital for pesticide resistance and nutrient assimilation [138,139]. These findings emphasize the need for further research into 4CSP’s interactions with GRP94 and actin, confirming its role in cellular processes.

**Figure 4:**
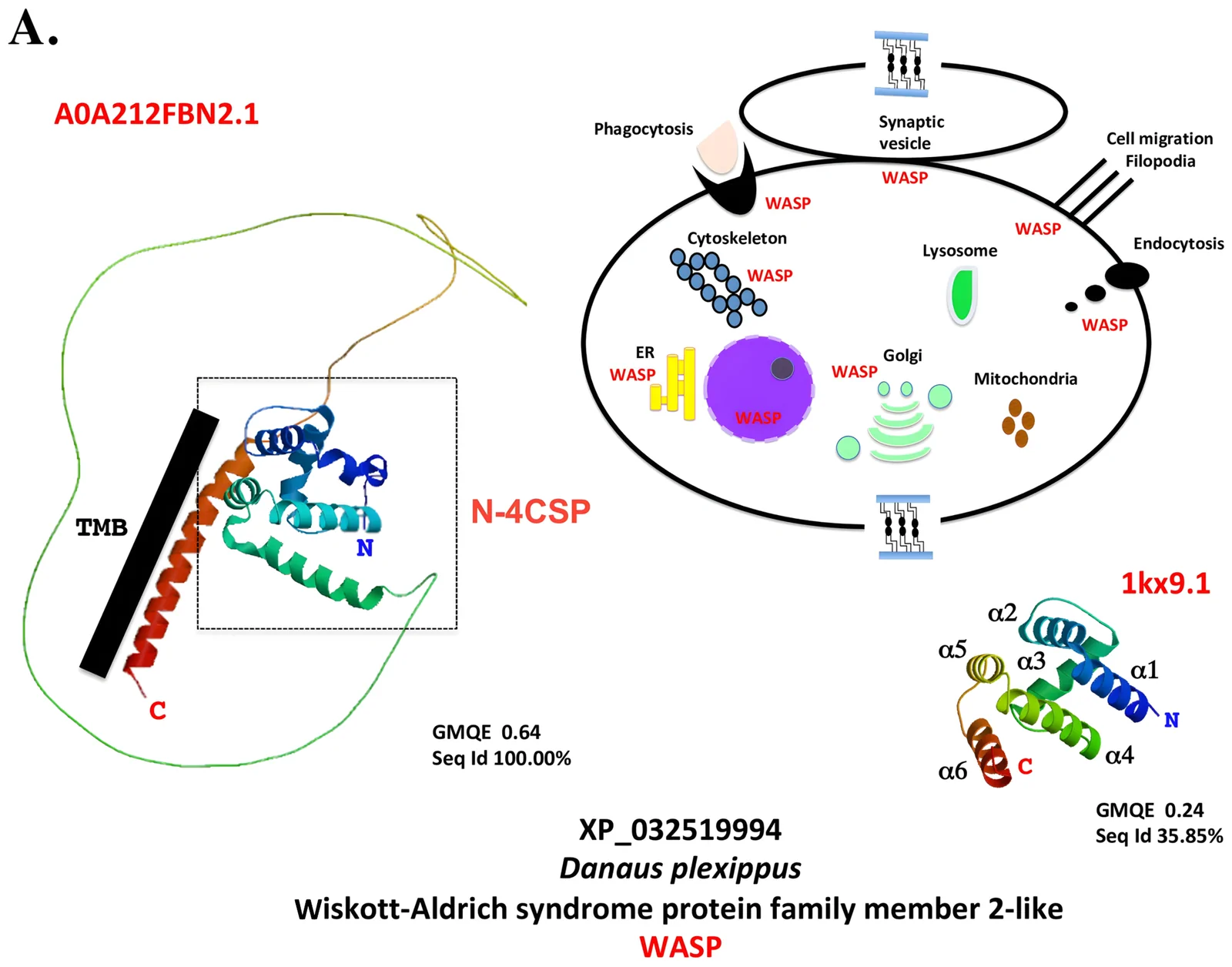

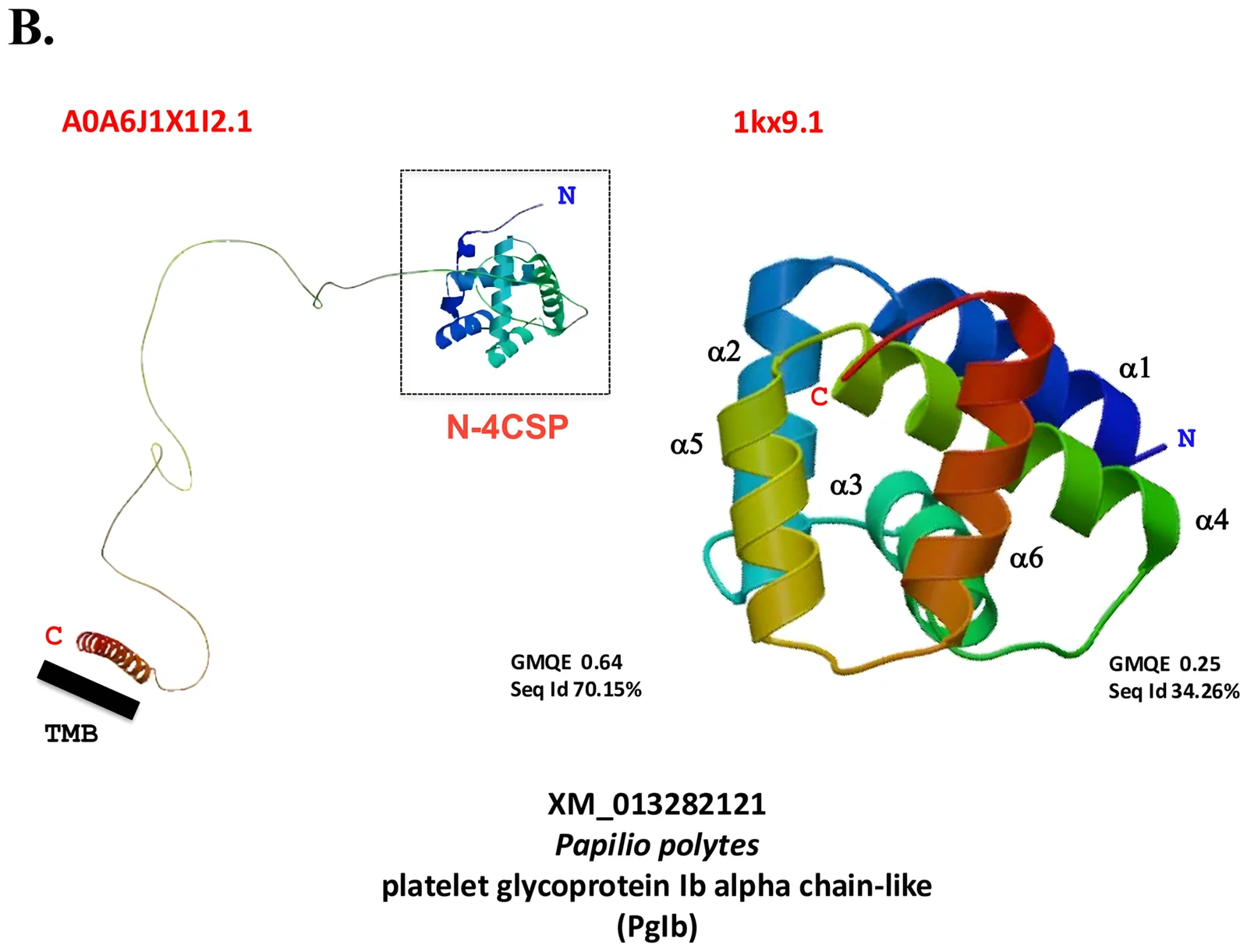
Molecular structure modeling of 4CSP and intracellular N-4CSP proteins. (**A**) Molecular structure modeling of Danpl-WASP (XP_032519994). (**B**) Molecular structure modeling of Pappo-PgIb (XM_013282121). Danpl-WASP and Pappo-PgIb sequences were aligned with Bommo-4CSP1 in order to identify the signal peptide and cut it off based on Edman sequencing [7]. The amino acid sequence of the mature protein was then subjected to molecular structure modeling using SWISS-MODEL Workspace/GMQE. The molecules with the highest identity score were used as template references: 1kx9.1 (“Chemosensory Protein A6”, X-ray, 1.6 Å, monomer, cabbage moth, *Mamestra brassicae*) and A0A212FBN2.1.A (WASP family member 2-like, AlphaFold DB model of A0A212FBN2_DANPL, LOC116772069 gene, monarch butterfly, *D. plexippus*) for WASP (in **A**); 1kx9.1 and A0A6J1X1I2.1.A (Mucin-2-like, AlphaFold DB model of A0A6J1X1l2_GALME, LOC113521739 gene, greater wax moth, *Galleria mellonella*) for PgIb (in **B**). For WASP (**A**) and PgIb (**B**), the Global Model Quality Estimation (GMQE) and the percentage of Sequence Identity (Seq Id) are shown. C: C-terminus, N: N-terminus. The α-helices that make up the 4CSP structure are numbered 1 through 6. The location and function of WASP in the cytoskeleton, ER, Golgi, filopodia, nucleus, and near endocytotic, phagocytotic, and synaptic vesicles point to the N-terminal tail of WASP and PgIb protein molecules in the intracellular compartment as the location of the 4CSP structure. The 4CSP prism is indicated by the black square with dotted lines. The two molecules share the same model of construction inside the cell: N-terminus 4CSP prism (N-4CSP), long loop, and transmembrane domain. The black bar indicates the position of the transmembrane segment (TMB; Swissmodel.expasy.org and services.healthtech.dtu.dk).

We propose a new paradigm that contrasts with the traditional olfactory model. The phylogenetic and modeling analysis of Mp10 orthologs reveals evolutionary relationships among intracellular protein families linked to actin and various organelles. While evolutionary analysis does not determine function, focusing on the connections between proteins like 4CSP, Rho, TIF, ASRP, SamkC, NPCP, viral tegument proteins, and many others suggests new research directions, particularly related to cellular roles consistent with the distribution and development of 4CSPs (see [1,140] and Figure 3 and Figure 4).

The relationship between 4CSP, Mp10, mucin, TIF, and various intracellular regulatory components challenges the notion of 4CSPs being chemosensory molecules. Comparison of Mp10 with the peplomer and cross-linkers of the inner tegument indicates that both bacteria and viruses express it. This shifts the focus of 4CSP research towards the detection of virus particles, distinguishing it from the study of scents and plant odors (see Figure 3 and Figure 4 and Tables S2–S4).

### 6.1. Evolutionary Evidence Derived from Phylogenetic Analysis

The first phylogenetic analysis of 4CSPs used bacterial 4CSPs, maximum likelihood (ML), and an empirical Bayesian approach in IQ-Tree [141], revealing relationships among various groups of proteins, including 4CSPs, Cell Wall Proteins, Rho, Sec31, TIF, SamkC, WAS/WASL, DNA-binding proteins (DNA-BPs), DNA-regulatory proteins (DNA-RPs), and various ribonucleic acid-binding proteins (RBPs), among others [140,141]. The follow-up analysis of insect Mp10-4CSPs (this study) employed the neighbor joining (NJ) algorithm and maximum parsimony (MP) to further investigate protein group relationships. The study revealed that these intracellular proteins show some common evolutionary processes, often interbreeding with the “chemosensory” 4CSP family, which was rather unexpected (see the branches in red in Figure 3).

We chose NJ and MP due to their involvement with various intracellular proteins and enzymes, anticipating a rather heterogeneous evolutionary process characterized by diverse evolutionary models and a high rate of change over time. NJ analysis is perfect for heterogeneous data because it does not assume a constant molecular clock, while MP finds the evolutionary tree with the fewest changes. Both NJ and MP are highly effective in complex models with fast change rates relevant to 4CSPs and intracellular proteins. Orthology grouping reveals that 4CSPs are grouped with allergens (IgE-binding proteins) in the TIF, PAN, and ASRP protein groups, indicating intracellular functional expression (see Figure 3 and Figures S2–S4). These proteins have roles in nucleotide binding, transcription, translation, trafficking of transcription factors, and/or actin regulation, which encourages additional CRISPR research to investigate the intracellular functions of 4CSPs.

In order to reconstruct phylogenetic trees using NJ or MP methods, PAUP* does not perform de novo multiple sequence alignment; instead, it requires an already aligned matrix, usually in NEXUS format. ClustalX was used to handle sequences of different lengths by matching conserved regions and inserting gaps during progressive alignment [142]. Trimming of amino acid sequences was avoided to preserve valuable data from longer sequences because there were no missing data issues [143]. There were roughly 100 amino acid sequences in the dataset, ranging in length from ~120 to ~500 residues. This was achievable without reducing the number of amino acids to 120, which would eliminate important information from longer sequences (Figure S3). Instead, care was taken to ensure shorter sequences aligned to the same common domain as longer ones, allowing meaningful distance calculations despite initial length disparities. If one short sequence maps to the protein’s N-terminus and the other to its C-terminus with zero overlap, the algorithm is unable to calculate the relative distance between them; however, this is not the case in our study (Figure S3). The N-terminus of the longer proteins is mapped to all of the short sequences (4CSPs, Pfam Domain OS-D/A10; Figure S3), which enables us to refer to them as “N-4CSPs” (see Figure 3).

To demonstrate the connection between 4CSPs and groups of intracellular proteins, we used a variety of phylogenetic analysis methods and different sets of taxa. We added a common ancestor node (outgroup) to the “unrooted” trees (examining the relationships between various groups) in order to determine an evolutionary path [10,144]. Based on their location in the evolutionary tree of bacterial 4CSPs, we chose *A. baumannii* 4CSPs and Bommo-CSP2 as the outgroup [14,140]. *A. baumannii*’s origin has deep ancestral roots [145]. In the investigation of insect 4CSPs and intracellular derivatives, we considered that CRAB 4CSPs could be utilized as a known relative that branched off early because they represented a late duplication when we looked at the development of bacterial 4CSPs and HGTs [14]. In an analysis of long 4CSPs (“N-4CSPs”), any other short 4CSPs would have been trustworthy to employ as an outgroup.

Using Myzpe-Mp10 in conjunction with 4CSPs, xenobiotics responsive elements (XREs), Mucins, and RBPs in PAUP analysis revealed that Mp10 is more closely related to the Mucins group (G1) than the 4CSP group (Figure S2A). One of the main conclusions was that ‘EbalCSP3’ and Rho-activator isoform X3 were significantly comparable, and their association with G1, which included Bommo-4CSP10, was shown by a strong bootstrap value (94%; Figure S2A). Interestingly, ‘EbalCSP4’ showed a clear divergence from the 4CSP and Mucin groups. The BioNJ study found that “chemosensory proteins” are strongly linked to several intracellular protein families and suggested renaming “CSPs”. It also suggested utilizing CRISPR techniques for further intracellular investigation.

Using Mp10 in conjunction with intracellular protein families, the analysis indicates that Mp10 does not cluster with the other 4CSPs. The closest similarities are between Mp10 and mucin proteins, while 4CSPs are related to allergens (Figure 3 and Figure S4). This supports the assertion that 4CSPs have minimal impact on chemosensing, with TIF linked to Mp10 reinforcing this claim. Furthermore, the presence of 4CSPs across mucin-, nuclear pore-, and actin-related sequences suggests they do not play a role in olfactory functions (see Figure 3 and Figures S3 and S4). Instead, the study suggests focusing on the role of 4CSPs in nuclear and actin sites, strongly emphasizing their involvement in Rho-activator proteins and cellular processes. It emphasizes that 4CSPs and small GTP-binding Rho proteins like “RhoGAPs” (XM_056065396) are intracellular regulators of actin cytoskeleton-related pathways [146]; therefore, their functional significance—rather than their connection to OR activation—should be the main focus.

The 4CSP sequences can be categorized into the ASRP and Allergen groups based on UPGMA analysis, suggesting ancestral origins for the latter (see Figure S4A). The ASRP group comprises outer envelope proteins, nuclear nucleoside kinases (NNKs), and RickA-like components (Arp2/3), while the Allergen group includes IgE-binding proteins (Thap1) and others linked to cell motility and gene regulation (see Figure 3 and Figure S4) [147,148,149,150]. Therefore, the evolution of 4CSP diverges from that of ORs and OBPs [151,152], tracing back to a distantly shared ancestral origin among Mp10/4CSP, Allergen, Thap1, TIF, Rho, ASRP, NPCP, and Mucin (Figure 3 and Figure S4). Allergen, which includes *4CSP* and *TIF* genes, appears to be the result of multiple duplication events. Mp10 and Rho emerged before further multiple duplications (d1-d5) led to various Mucin variants, notably in mosquitoes (Figure S4) [153,154]. UPGMA tree analysis, based on the Molecular Clock Hypothesis [155], indicates that these gene duplications are noted across various taxa, including ants, butterflies, damselflies (*Ischnura* forktails), flies, flour beetles, pierids, ladybirds, lice, neodiprions, parasitoid wasps, and tuberworm moths (Figure 3 and Figure S4 and Table S2), illustrating an evolutionary gap between ASRPs/4CSPs and Allergen/Thap1. Our evolutionary analysis indicates that *Allergens*, *TIF*, *Rho*, *ASRPs*, *NPCPs*, and *Mucins* arose from common ancestral duplications, preceded by *Mp10* and other *4CSP* variants (see Figure 3 and Figure S4). Our analysis reveals ancient gene duplications across numerous taxa, including different insect groups. This suggests that these protein families may date back to the Devonian or Mississippian periods, prior to the emergence of flying insects [156].

Maximum parsimony (MP) analysis in PAUP* confirmed strong relationships among the Mp10, 4CSP, Rho, Allergen, TIF, Mucin, NPCP, and ASRP protein groups, evidenced by high bootstrap values (89–100%; Figure S4B). Notably, the grouped proteins showcase a close connection, particularly with NPCP and Mucin at approximately 99%. The ASRP/Rho and Mucin/NPCP relationships are also supported by high Jackknife values (93–99%; Figure S4B). Additionally, 4CSPs share significant relationships with PAN and ASRP (94–100% bootstrap values; Figure S4B). These proteins are extensively expressed across adult body parts and in developmental stages (Tables S2 and S3), engaging primarily in DNA/protein interactions and enzymatic functions [157,158]. The links with intracellular proteins and gene promoter regions warrant further investigation, suggesting that CRISPR-4CSP might focus on more common intracellular functions.

The circular NJ phylogenetic tree further illustrates interconnections among protein groups such as 4CSP, Allergen, TIF, PAN, Rho, ASRP, NPCP, and Mucin (see Figure 3). High bootstraps (100%) suggest that 4CSPs have no role in olfaction, as they are primarily intracellular and influence sensory processes in nerve tissues (see [1]). Our study shows that Mp10 and TIF share similar evolutionary pathways, while ASRPs always cluster closely with Mp10 (Figure 3 and Figures S3 and S4), indicating rapid evolution despite their ancient origins [159,160,161,162]. Our study proposes a new working hypothesis that TIFs, ASRPs, and 4CSPs may interact together to control the actin skeleton and ribosomal gene expression, although further experimental evidence is strongly needed.

Our phylogenetic evolutionary analysis identified proteins related to Allergen/Mp10 across various insect taxa, including aphids, beetles, dragonflies, flies, lice, mosquitoes, moths, and sawflies. These proteins are intracellular and belong to groups like Mucin, NCPC, ASRP, and TIF, unrelated to taste or chemosensing. Their common root indicates that these protein groups diversified prior to insect emergence during the Carboniferous Period (~299–359 Mya; see Figure 3). The *4CSP* gene underwent multiple duplications (d1–d5), with early duplications (d1–d2) producing Allergen/Mp10, TIF, and PAN, and late duplications (d3–d5) forming Rho, ASRPs, NPCPs, and Mucins (referred to as N-4CSPs; see Figure 3 and Figure S4). Our phylogenetic study shows that 4CSPs and N-4CSPs have an old common ancestor. The *N-4CSP*/*Mucin* group was formed by five sequential duplications beginning with *4CSP* (see Figure 3 and Figures S3 and S4 and Table S2).

### 6.2. Intracellular Function Derived from Amino Acid Sequence Modeling Analysis

In addition to shared phylogeny, protein amino acid sequence alignment and structure modeling show further functional links between 4CSPs and many groups of intracellular proteins (see Figure 4 and Figure S3). Interestingly, 4CSP and the sequences of TIF, ASRP, NPCP, and Mucin have an exact N-terminal match (Figure S3), which enables us to refer to all of these protein groups as “N-4CSPs”. The majority of N-4CSP proteins show a similar tunnel-like configuration of six α-helices using SWISS-MODEL Workspace/GMQE [163], especially in the N-terminus (Figure 4). The highly distinctive 4CSP structure [15,16,17,18,19,20] was present in allergens Thap1, acid trehalase, Phk-3, Bommo-4CSP, Mp10, immune response proteins, and *Coccinella septempunctata* nuclear TIF (Figure S5). With unique characteristics in its structure and gene locus, the Danpl-WASP protein, which has a large transmembrane domain, is involved in filament synthesis and actin cytoskeleton regulation [164,165]. These big N-4CSP molecules, like the WASP structure, are found in intracellular organelles such as the cytoskeleton, microtubules, integuments, cell wall, nuclear pore, and cytoplasm. Important discoveries show that the N-terminus and C-terminal domains are connected by a sizable loop in N-4CSPs (see Figure 4 and Figure S5).

The structure of the protein CAH2235359 from the Palearctic butterfly, known as “jg5928,” suggests that the 4CSP protein may be present in viral particles (see Figure S5). Like WASP and PgIb, Jg5928 folds into a prominent loop that links the 4CSP domain to the transmembrane domain of the virus’s principal outer envelope glycoprotein (BLLF1, pfam0519; Figure S5). It is connected to viral envelope glycoproteins, indicating a role in viral attachment to host lipids, possibly via the 4CSP domain. Some bacterial proteins also have a similar structure, with a transmembrane region connected to a glycoprotein that contains the 4CSP domain (C-4CSP; see [14]). This structural resemblance raises questions about the significance of 4CSP in immune response and infection processes, with an emphasis on its possible function in binding with bacterial and viral components rather than its previously considered chemosensory roles.

### 6.3. “4CSP, Intracellular Mode of Action” Derived from Location, Size, Structure, and Expression in Microbes and Viruses

**Figure 5:**
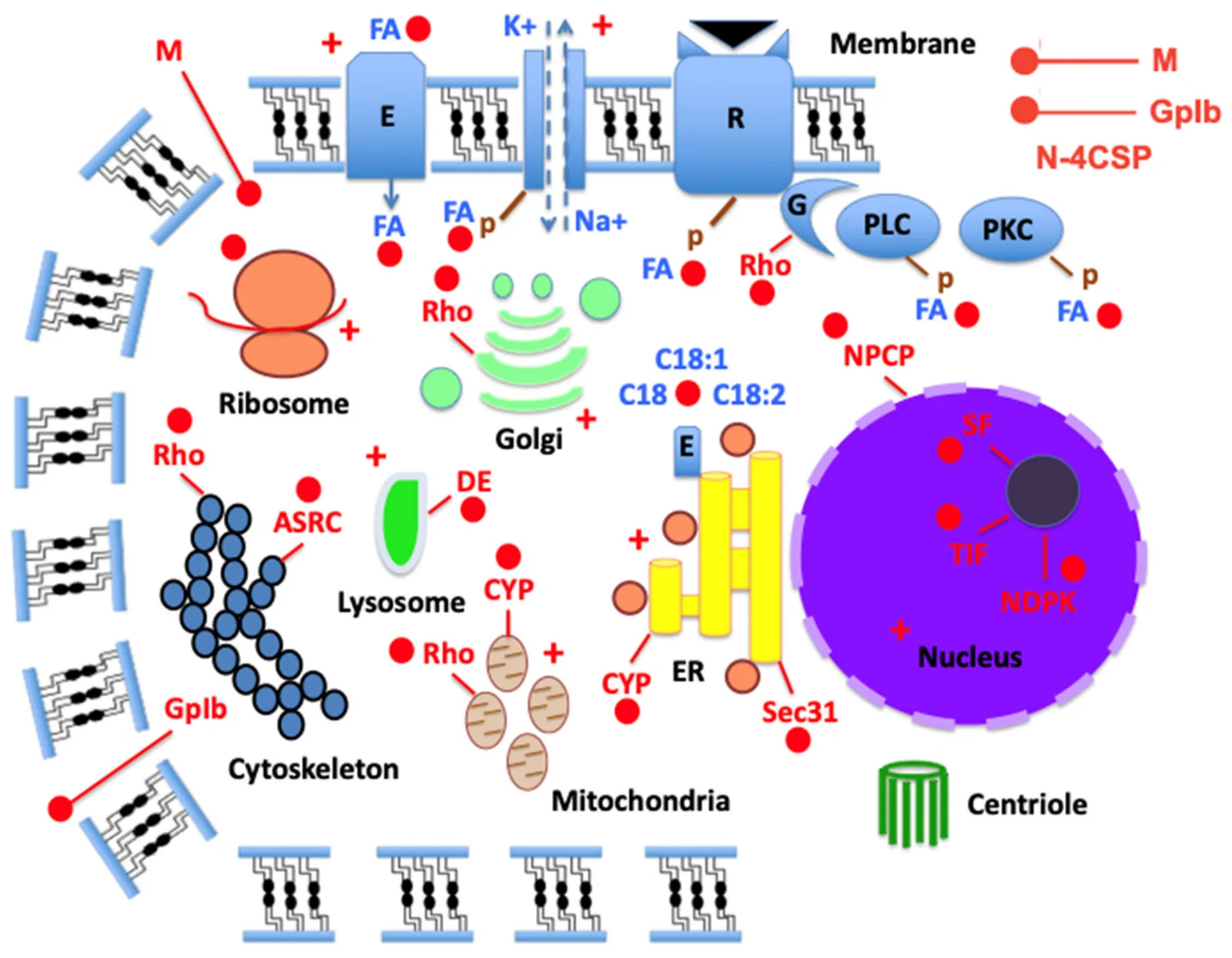
The “4CSP Intracellular Mode of Action” Hypothesis. 4CSP binds to fatty acid (FA), which mediates the phosphorylation (p) of different plasma membrane-bound protein molecules. K+ intake/Na+ outflow: potassium–sodium exchange pump, R: Receptors, G: G-protein, PLC: phospholipase kinase C, PKC: protein kinase C, Rho: Rho GTPase, ER: endoplasmic reticulum, E: Desaturase Enzyme, which interacts with 4CSPs that transport FAs, such as linoleic acid (C18:2) and its precursors (stearic acid C:18 and elaidic acid C:18-1). +: Stress reactions that lead to 4CSP interacting with different molecules in the membrane, nucleus, ribosome, Golgi, lysosome, ER and mitochondria, including cytochrome P450 (CYP), Degradative Enzyme (DE), Sec31 protein complex (Sec31), and Mucin (M). 4CSP proteins attach to the actin skeleton regulatory complex (ASRC), Gplb-like protein (Gplb), and nuclear pore complex protein (NPCP) in the actin cytoskeleton, plasma membrane, and nuclear membrane, respectively. Nucleoside diphosphate kinase (NDPK), splicing factor (SF), and transcription initiation factor (TIF) are bound in the nucleus. M also stands for mucin-like fractions, secretory molecules that bolster the immune system’s protection against microbial stress, while PgIb plays a part in the cell-adhesion system (LRR motif). Based on phylogeny and modeling studies of the amino acid sequence, the large red dots represent each potential protein, protein assembly, or supramolecular complex in each intracellular organelle that 4CSP may interact with in both insects and hexapods (see Figure 2, Figure 3 and Figure 4 and Figures S1–S5).

Given the similarities among 4CSP and several groups of intracellular proteins, it is hypothesized that 4CSP has diverse intracellular functions in insects and hexapods (see Figure 5). To explore the “4CSP intracellular mode of action”, further research using CRISPR, RNAi, or gene knockout is advised, in conjunction with general cell biology, biochemistry, and molecular biology approaches. Quantifying lipids in cells and locating 4CSP close to organelles including the ER, nuclear pores, mitochondria, and Golgi may be an essential part of this study.

The “4CSP intracellular mode of action” hypothesis states that 4CSP interacts with linoleic acid (LA, C18:2, ω-6 fatty acid) based on G. Liu et al. [21,22]. LA is derived from arachidonic acid (ARA, C20:4, ω-6 fatty acid), which is essential for the production of hormones such as prostaglandins, thromboxanes, and leukotrienes. Ion transfer, innate immune response, egg formation, and reproduction are only a few of the physiological processes that these hormones control [166,167,168]. Furthermore, C18:2 and C20:4 ω-6 FA lipids function as intracellular modulators, affecting a range of proteins and phosphorylating many signal transduction pathways and physiological processes (see [22]).

Insect growth and stress responses depend on these 4CSPs’ role in phosphorylation and lipoid activity within several intracellular organelles (see Figure 5). The widespread expression of the LA-4CSP complex throughout the insect body indicates a significant mediating role in various physiological functions (see [21]). LA influences Ca^2+^ signaling and kinase phosphorylation across gustatory and metabolic cells, as well as activating multiple signaling pathways in mammals [169,170]. Moreover, LA targets vital tissues in insects, such as the brain and gut, participating in oxidative phosphorylation linked to immune responses [171,172,173,174,175,176]. The reticulum, Golgi, mitochondria, lysosomes, ribosomes, proteasomes, microtubules, and actin cytoskeleton are all targets of LA [177,178,179,180,181,182]. 4CSP is exclusively intracellular, associated with LA and other lipids, emphasizing its relevance in general cell biology rather than sensory processes.

In summary, 4CSPs play a crucial role in regulating lipid metabolism during the cell cycle in various organisms, including bacteria, insects, and hexapods [1,14,21,22,183,184,185,186]. They interact with cytochrome P450 enzymes under stress conditions, impacting cellular metabolism, homeostasis, and detoxification linked to insecticide exposure [2,3,187,188]. Additionally, 4CSPs would be involved in the lysosomal defense against pathogens and the degradation of macromolecules (Figure 5). The hypothesis suggests that close cooperation between 4CSPs, fatty acids (FAs), and the ER is essential for lipid droplet production and sex pheromone biosynthesis (Δ-desaturases; [189,190,191,192]). Our study proposes using CRISPR to explore these interactions and functions further, supported by evidence of 4CSP tissue distribution and their role in lipoid biosynthetic pathways.

The “4CSP intracellular mode of action” hypothesis also states that 4CSPs interact with a variety of cellular elements, such as the nucleus and different cell membranes, via the Rho GTPase signaling complex and related intracellular proteins (see Figure 3, Figure 4 and Figure 5). They may play a role in FA lipid and protein transport, particularly between the nucleus, the Golgi apparatus, and lysosomes [193,194,195] (Figure 5). The 4CSP Pfam domain is part of multiple proteins such as Pglb, resembling a membrane glycoprotein receptor, indicating an ancient evolutionary origin tied to glycoprotein mechanisms [196]. This implies that 4CSPs are related to roles in bacteria, worms, and the ancient enteric endocrine system in ecdysozoans, and that they largely precede olfactory receptors. This connects 4CSPs to the intracellular CYP-P450 family, which has been associated with metabolic processes and mitochondria since Bya [14,197,198,199].

## 7. Concluding Remarks and New Perspectives

Our study emphasizes the evolutionary and physiological parallels between 4CSPs and Mucins. Both protein groups are necessary for insect growth and development and work together to control immunological and gastrointestinal responses [1,2,200,201,202,203,204,205,206,207,208]. Interestingly, blood feeding increases the expression of 4CSPs and Mucins in mosquitoes [209,210,211], indicating that 4CSPs have similar functions to Mucins in many insect species.

The most crucial factor for new study direction in this regard may be the fact that Mucins, which are abundantly expressed in true bug salivary glands and secreted into rice during feeding, elicit plant immune responses in a way similar to that of Mp10 [212]. This shows the importance of these two protein groups distant from olfactory receptors. Furthermore, our study shows that 4CSPs interact not only with Mucins but also with many other intracellular proteins, including ASRPs and NPCPs, providing insight into non-chemosensory functions and possible intracellular roles that call for more research.

During the “OS-D/CSP” era (1994–2003), the connections between 4CSPs, Mucins, ASRPs, NPCPs, cytoskeleton complexes, and other nuclear genetic elements were rather inconceivable. However, the non-chemosensory roles of this protein family have been highlighted by more recent studies, particularly with regard to insecticide resistance [1,2,3,14,21,22,67]. On the other hand, little is known about intracellular roles associated with 4CSP, Mucin, ASRP, NPCP, and cell regulation. Since their functions in actin and nuclear function are not yet supported by experimental data, the evolutionary link of “OS-D/CSP” requires a name change and new research perspectives. Instead of focusing on “OS-D/CSP” in olfaction, future research should examine the biological roles of 4CSPs in intracellular activities, strongly suggesting a change toward researching their impact on cell functions, notably with CRISPR technologies.

Numerous studies have demonstrated that 4CSPs are found in a variety of tissues, away from sensilla, cuticles, and teguments (see [1]). The “Mp10 experiment” reveals significant sequence homology with Mucin, suggesting a more extensive function for 4CSP in intracellular contexts. Actin-related proteins and nuclear complexes are among the bigger molecules it interacts with (see Figure 1, Figure 2, Figure 3, Figure 4 and Figure 5 and Figures S1–S5). Future research should focus on validating the structure of proteins containing 4CSP at the N-terminus (hence dubbed “N-4CSP”) using X-ray or NMR, emphasizing the intracellular aspects of 4CSP’s role.

Additionally, using CRISPR to cut the 4CSP at the transcription initiation factor’s N-terminus could provide insights into its role in cellular function. The 4CSP is linked to various intracellular complexes, suggesting its function as a regulatory module rather than merely scavenging odorant molecules. It is present in many tissues (see [1]) and implicated in processes like lipid metabolism, significantly impacting gene expression and intracellular signaling pathways. Interactions between 4CSPs, Mucins, and lipids indicate a complex role in transporting and aggregating lipid molecules, warranting further investigation through binding assays and X-Ray studies [213,214,215,216,217,218,219].

There is consensus regarding the protein’s role in transporting lipoid molecules, particularly in insects like moths that use 4CSPs and FAs for sex pheromone production. Moreover, the position of 4CSPs inside the cell in both bacteria and insects is firmly supported by a function associated with lipid metabolism. It is interesting to note that bacteria express “C-4CSPs”, whereas insects have “N-4CSPs”. These proteins are found inside the cell and nucleus, in all of the many cell organelles, and in all of the functions of bacteria, viruses, insects, and hexapods (see Figure 5), with Mucins, 4CSPs, and OBPs showcasing strong interactions with lipoids. While 4CSPs may transport long-chain FAs, Mucins could facilitate lipoid aggregation and droplet formation, indicating a protective mechanism in the insect tissue. Further investigation into the interaction of Mucins with short-chain FAs and 4CSPs with long-chain FAs is warranted.

Based on its tissue distribution, developmental profile, pesticide sensitivity, and binding of intracellular lipids like LA, the “CSP/OS-D” protein family does not appear to be involved in olfaction [1,220]. Therefore, prior names such as OS-D/A10, CSP, and SAP “sensory appendage protein” are totally inappropriate. Instead, it is advised to rename the protein to “4CSP”, which reflects its structure, prior to its capacity to associate FAs, lipoids, TIFs, nucleic acids, actin, and other intracellular components. In order to focus on the novel investigation of intracellular processes, this protein family has to be renamed.

## Data Availability

The original contributions presented in the study are all included in the article/Supplementary Materials; further inquiries can be directed to the corresponding author.

## Supplementary Materials

The following supporting information can be downloaded at: https://www.mdpi.com/article/10.3390/insects17090940/s1, Figure S1: Alignment of the amino acid sequence of Mp10 with those of other aphid and hemipteran sequences, as well as their InsectBase counterparts in Coleoptera, Hymenoptera, and Lepidoptera; Figure S2: PAUP*10Altivec phylogenetic analysis of insect 4CSPs, DBPs, and RBPs; Figure S3: Myzpe Mp10’s alignment with related proteins from the 4CSP, Allergen, Mucin, Rho, TIF, ASRP, and NPCP families; Figure S4: UPGMA and Bootstrap/Jackknife analyses of Mp10 and intracellular counterparts; Figure S5: The modeling of the molecular structures of Mp10 and its related proteins belonging to the Allergen, Mucin, Rho, TIF, ASRP, and NPCP families in SWISS-MODEL. Table S1: Acypi ‘4CSP’ gene repertoire in comparison to other Hemiptera, genome assembly, and protein identity; Table S2: Sequences producing significant alignments with Mp10; Table S3: Sequences producing significant PAUP-Tree between 4CSP and intracellular proteins; Table S4: Sequences of RNA-binding proteins (RBPs) used in PAUP-tree for molecular comparisons with 4CSPs and other intracellular proteins [2,14].

## Abbreviations

The following abbreviations are used in this manuscript:

4CSP	4-Cysteine Soluble Protein (“CSP/OS-D”)
6CSP	6-Cysteine Soluble Protein (“OBP”)
AcrR	Regulator of adjacent acrAB efflux genes
Acypi	*Acyrthosiphon pisum* (pea aphid)
Aedae	*Aedes aegypti* (dengue yellow fever mosquito)
AKH	Adipokinetic hormone
AI	Auto-inducer
Allergen Tha p 1	IgE-binding protein (15 kDa) and major allergen of pine processionary caterpillar (*Thaumetopoea pityocampa*, Thapi)—variant 1
Anoga	*Anopheles gambiae* (African malaria mosquito)
ARA	Arachidonic acid
Arp2/3	Actin-related protein 2/3 complex
ASRC	Actin skeleton regulatory complex
ASRP	Actin skeleton regulatory protein
Avd	Accessory variability determinant
Bemta	*Bemisia tabaci* (silverleaf whitefly)
Bemta-4CSP1	*Bemisia tabaci* “Chemosensory Protein”-1 renamed to Bemta-4CSP1
BioNJ	Improved version of Neighbor Joining algorithm based on simple model of sequence data
BLASTp	Protein BLAST
BLLF1	Epstein–Barr virus envelope glycoprotein encoded by BLLF1 gene
Bomte	*Bombus terrestris* (buff-tailed bumblebee)
C-4CSP	Long intracellular proteins with a C-terminus 4CSP (Pfam OS-D/A10)
CA	Corpora allata
CHC	Cuticular hydrocarbons
Chisu	*Chilo suppressalis* (Asiatic rice borer or striped rice stemborer)
CNS	Central nervous system
Cocse	*Coccinella septempunctata* (seven-spot ladybird)
CRISPR	Clustered Regularly Interspaced Short Palindromic Repeats
CSP	Chemosensory protein
Culpi	*Culex pipiens* (common house mosquito)
CWA	Cell wall anchored
CWP	Cell wall protein
CYP	Cytochrome P450
DAN4	Cell wall mannoprotein expressed under delayed anaerobic conditions (*Saccharomyces)*
Danpl	*Danaus plexippus* (monarch butterfly)
DE	Degradative enzyme
Denpo	*Dendroctonus ponderosae* (mountain pine beetle)
DGR	Diversity-generating retroelement
DNA-BP	Deoxyribonucleic acid-binding protein
DNA-RP	Deoxyribonucleic acid-regulatory protein
DP	Diphosphate
Drome	*Drosophila melanogaster* (fruit fly)
EA	Elaidic acid
Ebsp	Ejaculatory bulb-specific protein (also called Peb)
ER	Endoplasmic reticulum
Eupco	*Eupeodes corollae* (migrant overfly)
FA	Fatty acid
Fox	Forkhead box (transcriptional regulator, cell growth regulator)
GDP	Guanosine diphosphate
GMQE	Global Model Quality Estimate
GOBP	General Odorant Binding Protein
GpIb	Glycoprotein Ib receptor complex
GR	Gustatory receptor
GRP94	Glucose-regulated protein 94—Endoplasmic Reticulum (ER) chaperone
GTP	Guanosine triphosphate
Halha	*Halyomorpha halys* (brown marmorated stink bug)
Helme	*Heliconius melpomene* (postman butterfly)
HGT	Horizontal gene transfer
Histone-like	Histone-like RNA/DNA-binding protein family (nucleus)
Hu IgE	Human immunoglobulin E
IgE-BP	Immunoglobulin E-binding protein
Iphpo	*Iphiclides podalirius* (scarce swallowtail)
IR	Ionotropic receptor
Jg5928	Major outer envelope protein
JH	Juvenile hormone
LA	Linoleic acid (cis,cis-9,12-octadecadienoic acid C18:2)
LMAN1-MCFD2	Calcium-dependent cargo receptor complex that transports certain glycoproteins from the ER to the Golgi apparatus
LRR	Leucine-rich repeat protein complex
M	Mucin
Mambr	*Mamestra brassicae* (cabbage moth)
ML	Maximum likelihood
MP	Maximum parsimony
Mp10	Myzpe p10-like protein
Mraz	DNA-binding transcription factor encoded by mraZ
Myzpe	*Myzus persicae* (green peach aphid)
N-4CSP	Long intracellular proteins with an N-terminus 4CSP (Pfam OS-D/A10) domain
NDPK	Nucleoside diphosphate kinase
Nillu	*Nilaparvata lugens* (brown planthopper, BPH)
NME	Nucleotide metabolism enzymes
NNK	Nuclear nucleoside kinase
NPCP	Nuclear pore complex protein
OBP	Odorant-binding protein
ODE	Odorant degrading enzymes
OR	Olfactory receptor
OS-D	Olfactory Specific-type D protein
OXPHOS	Oxidative phosphorylation
P	Phosphate
Pacve	*Pachypsylla venusta* (hackberry petiole gall psyllid)
PAN	Hexameric ATPase complex
PAN-1	Protein encoded by the pan-1 gene in the nematode *Caenorhabditis elegans*
Pappo	*Papilio polytes* (common Mormon)
PAUP	Phylogenetic analysis using parsimony
PBP	Pheromone Binding Protein
Pedhu	*Pediculus humanus humanus* (human body louse)
PG	Prothoracic gland
PgIb	Platelet glycoprotein Ib alpha chain-like
Phk	Pherokine (hemolymph protein)
PKC	Protein kinase C
PLC	Phospholipase C
QS	Quorum sensing
Ras	Family of GTPases derived from rat sarcoma virus
RBP	Ribonucleic acid-binding protein
Rho	Family of GTPases, family of small (~21 kDa) signaling G proteins, subfamily of the Ras superfamily
RhoGAP	Rho GTPase-activating protein, regulator of Rho-related protein family (motility, contractility, growth, differentiation, development)
RickA	*Rickettsia conorii* surface protein A (activator of Arp2/3)
RNAi	RNA interference
SA	Stearic acid
SamkC	Serine/Threonine-protein kinase C (from social amoeba)
SAP	Sensory appendage protein
Sec31	Protein transport protein encoded by the SEC31A gene (human)
SF	Splicing factor
SP	Signal peptide
Tat	Twin-arginine translocation pathway
TERT	Telomerase reverse transcriptase
TetR	Tetracycline repressor
TFTR	TetR-family transcriptional regulator
TIF	Transcription initiation factor
TP	Triphosphate
Trica	*Tribolium castaneum* (red flour beetle)
TSSC1	Tumor-suppressing subtransferable candidate 1 (endosomal machinery)
UL36	Large tegument protein deneddylase encoded by UL36 gene (herpesviridae)
UPGMA	Unweighted pair group method with arithmetic mean
Uralo	*Uranotaenia lowii* (pale-footed Uranotaenia)
WAS/WASL	Wiskott–Aldrich Syndrome/Wiskott–Aldrich Syndrome-like protein
WASP	Wiskott–Aldrich Syndrome protein
XRE (Xre)	Xenobiotic response element family of DNA-binding transcriptional regulators
YhbY	RNA-binding protein (folded like TIF) encoded by YhbY gene (*Escherichia coli*)
YLPM1	YLP motif containing 1 encoded by YLPM1 gene (human)

## References

[1] Liu G., Sun B., Fan W., Yue S., He Q., Picimbon J.F. (2026). Renaming the ‘OS-D/CSP’ family (Part 1): ‘4-Cysteine Soluble Proteins’ (4CSPs)—Molecular nomenclature, structure, expression, evolution, tissue-distribution, and pleiotropy. Insects.

[2] Xuan N., Guo X., Xie H., Lou Q., Lu X.B., Liu G., Picimbon J.F. (2015). Increased expression of CSP and CYP genes in adult silkworm females exposed to avermectins. Insect Sci..

[3] Guo X., Xuan N., Liu G., Xie H., Lou Q., Arnaud P., Offmann B., Picimbon J.F. (2021). An expanded survey of the moth PBP/GOBP clade in *Bombyx mori*: New insight into expression and functional roles. Front. Physiol..

[4] Pikielny C.W., Hasan G., Rouyer F., Rosbach M. (1994). Members of a family of *Drosophila* putative odorant-binding proteins are expressed in different subsets of olfactory hairs. Neuron.

[5] McKenna M.P., Hekmat-Scafe D.S., Gaines P., Carlson J.R. (1994). Putative *Drosophila* pheromone-binding-proteins expressed in a subregion of the olfactory system. J. Biol. Chem..

[6] Angeli S., Ceron F., Scaloni A., Monti M., Monteforti G., Minnocci A., Petacchi R., Pelosi P. (1999). Purification, structural characterization, cloning and immunocytochemical localization of chemoreception proteins from *Schistocerca gregaria*. Eur. J. Biochem..

[7] Picimbon J.F., Dietrich K., Angeli S., Scaloni A., Krieger J., Breer H., Pelosi P. (2000). Purification and molecular cloning of chemosensory proteins from *Bombyx mori*. Arch. Insect Biochem. Physiol..

[8] Picimbon J.F., Dietrich K., Breer H., Krieger J. (2000). Chemosensory proteins of *Locusta migratoria* (Orthoptera: Acrididae). Insect Biochem. Mol. Biol..

[9] Picimbon J.F., Dietrich K., Krieger J., Breer H. (2001). Identity and expression pattern of chemosensory proteins in *Heliothis virescens* (Lepidoptera, Noctuidae). Insect Biochem. Mol. Biol..

[10] Picimbon J.F., Blomquist G.J., Vogt R.G. (2003). Biochemistry and evolution of CSP and OBP proteins. Insect Pheromone Biochemistry and Molecular Biology, The Biosynthesis and Detection of Pheromones and Plant Volatiles.

[11] Wanner K.W., Isman M.B., Feng Q., Plettner E., Theilmann D.A. (2005). Developmental expression patterns of four chemosensory protein genes from the Eastern spruce budworm, *Choristoneura fumiferana*. Insect Mol. Biol..

[12] Truman J.W., Riddiford L.M. (2019). The evolution of insect metamorphosis: A developmental and endocrine view. Philos. Trans. R. Soc. Lond. B Biol. Sci..

[13] Haug J.T., Anger K., Harzsch S., Thiel M. (2020). Metamorphosis in Crustaceans. Developmental Biology and Larval Ecology: The Natural History of the Crustacea.

[14] Picimbon J.F. (2026). Molecular phylogeny of “Chemosensory Proteins” in bacteria and arthropods: *CSP* as an extremely ancient gene. J. Mol. Evol..

[15] Lartigue A., Campanacci V., Roussel A., Larsson A.M., Jones T.A., Tegoni M., Cambillau C. (2002). X-ray structure and ligand binding study of a moth chemosensory protein. J. Biol. Chem..

[16] Jansen S., Chmelik J., Zídek L., Padrta P., Novak P., Zdrahal Z., Picimbon J.F., Löfstedt C., Sklenar V. (2007). Structure of *Bombyx mori* Chemosensory Protein 1 in solution. Arch. Insect Biochem. Physiol..

[17] Tomaselli S., Crescenzi O., Sanfelice D., Ab E., Wechsel-Berger R., Angeli S., Scaloni A., Boelens R., Tancredi T., Pelosi P. (2006). Solution structure of a chemosensory protein from the desert locust *Schistocerca gregaria*. Biochemistry.

[18] Jia Q., Zeng H., Zhang J., Gao S., Xiao N., Tang J., Dong X., Xie W. (2021). The crystal structure of the *Spodoptera litura* Chemosensory Protein CSP8. Insects.

[19] Campanacci V., Lartigue A., Hällberg B.M., Jones T.A., Giuici-Orticoni M.T., Tegoni M., Cambillau C. (2003). Moth chemosensory protein exhibits drastic conformational changes and cooperativity on ligand binding. Proc. Natl. Acad. Sci. USA.

[20] Mosbah A., Campanacci V., Lartigue A., Tegoni M., Cambillau C., Darbon H. (2003). Solution structure of a chemosensory protein from the moth *Mamestra brassicae*. Biochem. J..

[21] Liu G., Ma H.M., Xie Y.N., Xuan N., Guo X., Fan Z.X., Rajashekar B., Arnaud P., Offmann B., Picimbon J.F. (2016). Biotype characterization, developmental profiling, insecticide response and binding property of *Bemisia tabaci* chemosensory proteins: Role of CSP in insect defense. PLoS ONE.

[22] Liu G., Xuan N., Rajashekar B., Arnaud P., Offmann B., Picimbon J.F. (2020). Comprehensive history of CSP genes: Evolution, phylogenetic distribution, and functions. Genes.

[23] Weisiger R.A. (2007). Mechanisms of intracellular fatty acid transport: Role of cytoplasmic-binding proteins. J. Mol. Neurosci..

[24] Bos J.I., Prince D., Pitino M., Maffei M.E., Win J., Hogenhout S.A. (2010). A functional genomics approach identifies candidate effectors from the aphid species *Myzus persicae* (green peach aphid). PLoS Genet..

[25] Rodriguez P.A., Stam R., Warbroek T., Bos J.I. (2014). Mp10 and Mp42 from the aphid species *Myzus persicae* trigger plant defenses in *Nicotiana benthamiana* through different activities. Mol. Plant-Microbe Interact..

[26] Bradford M.K., Whitworth K., Wendland B. (2015). Pan1 regulates transitions between stages of clathrin-mediated endocytosis. Mol. Biol. Cell.

[27] Esbin M.N., Cookis T., Anantakrishnan S., Abidi A.A., Karr J., Cattoglio C., Darzacq X., Tjian R. (2025). Assembly and dynamics of transcription initiation complexes. Annu. Rev. Biochem..

[28] Toshima J.Y., Furuya E., Nagano M., Kanno C., Sakamoto Y., Ebihara M., Siekhaus D.E., Toshima J. (2016). Yeast Eps15-like endocytic protein Pan1p regulates the interaction between endocytic vesicles, endosomes and the actin skeleton. eLife.

[29] Levin P.A., Angert E.R. (2015). Small but mighty: Cell size and bacteria. Cold Spring Harb. Perspect. Biol..

[30] Hug I., Deshpande S., Sprecher K.S., Pfohl T., Jenal U. (2017). Second messenger-mediated tactile response by a bacterial rotary motor. Science.

[31] Siliakus M.F., van der Oost J., Kengen S.W.M. (2017). Adaptations of archaeal and bacterial membranes to variations in temperature, pH and pressure. Extremophiles.

[32] Kojima K., Sudo Y. (2023). Convergent evolution of animal and microbial rhodopsins. RSC Adv..

[33] Kouyama T., Murakami M. (2010). Structural divergence and functional versatility of the rhodopsin superfamily. Photochem. Photobiol. Sci..

[34] Needham D.M., Yoshizawa S., Hosaka T., Poirier C., Choi C.J., Hehenberger E., Irwin N.A.T., Wilken S., Yung C.-M., Bachy C. (2019). A distinct lineage of giant viruses brings a rhodopsin photosystem to unicellular marine predators. Proc. Natl. Acad. Sci. USA.

[35] Nakabashi A. (2015). Horizontal gene transfers in insects. Curr. Opin. Insect Sci..

[36] Zhang S., He Z., Wang H., Zhai J. (2025). Signal peptides: From molecular mechanisms to applications in protein and vaccine engineering. Biomolecules.

[37] Wang Y., Yang J., Lee O.O., Dash S., Lau S.C., Al-Suwailem A., Wong T.Y., Danchin A., Qiang P.Y. (2011). Hydrothermally generated aromatic compounds are consumed by bacteria colonizing in Atlantis II Deep of the Red Sea. ISME J..

[38] Xu H., Wang X., Wei Y., Cao Y., Wang S., Xia P. (2025). Pathway crosstalk enables degradation of aromatic compounds in marine *Roseobacter* clade bacteria. Appl. Environ. Microbiol..

[39] Santos T.C.B., Futerman A.H. (2023). The fats of the matter: Lipids in prebiotic chemistry and in origin of life studies. Prog. Lipid Res..

[40] Pavlowsky A., Silva B., Basu R., Correia Delecourt A., Geny D., Danglot L., Plaçais P.Y., Preat T. (2025). Neuronal fatty acid oxidation fuels memory after intensive learning in *Drosophila*. Nat. Metab..

[41] Brown W., Ralston A., Shaw K. (2008). Positive transcription control: The glucose effect. Nat. Educ..

[42] Jeckelmann J.M., Erni B. (2020). Transporters of glucose and other carbohydrates in bacteria. Pflüg. Arch.-Eur. J. Physiol..

[43] Balmand S., Rivard C., Peignier S., Santarella-Mellwig R., Ghanem-Debbache M., Maire J., Engl T., Galvão Ferrarini M., Dell’Aglio E., Soriana-Saiz B. (2025). Bacterial tubular networks channel carbohydrates in insect endosymbiosis. Cell.

[44] Jurtshuk P., Baron S. (1996). Bacterial metabolism. Medical Microbiology.

[45] Martinez-Guryn K., Hubert N., Frazier K., Urlass S., Musch M.W., Ojeda P., Pierre J.F., Miyoshi J., Sontag T.J., Cham C.N. (2018). Small intestine microbiota regulate host digestive and absorptive adaptive responses to dietary lipids. Cell Host Microbe.

[46] Visser B., Scheifler M. (2026). Insect lipid metabolism in the presence of symbiotic and pathogenic viruses and bacteria. Adv. Exp. Med. Biol..

[47] Miller M.B., Bassler B.L. (2001). Quorum sensing in bacteria. Annu. Rev. Microbiol..

[48] Waters C.M., Bassler B.L. (2006). The *Vibrio harveyi* quorum-sensing system uses shared regulatory components to discriminate between multiple autoinducers. Genes Dev..

[49] Lerat E., Moran N.A. (2004). The evolutionary history of quorum-sensing systems in bacteria. Mol. Biol. Evol..

[50] Coolahan M., Whalen K.E. (2025). A review of quorum-sensing and its role in mediating interkingdom interactions in the ocean. Commun. Biol..

[51] Monnet V., Gardan R. (2015). Quorum-sensing regulators in Gram-positive bacteria: ‘cherchez le peptide’. Mol. Microbiol..

[52] Papenfort K., Bassler B.L. (2016). Quorum sensing signal-response systems in Gram-negative bacteria. Nat. Rev. Microbiol..

[53] Jin X., Brandazza A., Navarrini A., Ban L., Zhang S., Steinbrecht R.A., Zhang L., Pelosi P. (2005). Expression and immunolocalization of odorant-binding and chemosensory proteins in locusts. Cell. Mol. Life Sci..

[54] Steinbrecht R.A., Ozaki M., Ziegelberger G. (1992). Immunocytochemical localization of pheromone-binding protein in moth antennae. Cell Tissue Res..

[55] Sabatier L., Jouanguy E., Dostert C., Zachary D., Dimarcq J.L., Bulet P., Imler J.C. (2003). Pherokine-2 and -3: Two *Drosophila* molecules related to pheromone/odor-binding proteins induced by viral and bacterial infections. Eur. J. Biol..

[56] Jimenez L.V., Kang B.K., deBruyn B., Lovin D.D., Severson D.W. (2004). Characterization of an *Aedes aegypti* bacterial artificial chromosome (BAC) library and chromosomal assignment of BAC clones for physical mapping quantitative trait loci that influence *Plasmodium* susceptibility. Insect Mol. Biol..

[57] Loftus B.J., Utterback T., Pertea G., Koo H., Mori A., Schneider J., Lovin D., de Bruyn B., Song Z., Raikhel A. (2005). Aedes aegypti cDNA Sequencing.

[58] Noriega F.G., Ribeiro J.M.C., Koener J.F., Valenzuela J.G., Hernandez-Martinez S., Pham V.M., Feyereisen R. (2006). Comparative genomics of insect juvenile hormone biosynthesis. Insect Biochem. Mol. Biol..

[59] Nene V., Worthman J.R., Lawson D., Haas B., Kodira C., Tu Z., Loftus B., Xi Z., Megy K., Grabherr M. (2007). Genome sequence of *Aedes aegypti*, a major arbovirus vector. Science.

[60] Einhorn E., Imler J.L., Picimbon J.F. (2019). Insect immunity; from systemic to chemosensory organs protection. Olfactory Concepts of Insect Control-Alternative to Insecticides.

[61] Balabanidou V., Kampouraki A., MacLean M., Blomquist G.J., Tittiger C., Juárez M.P., Mijailovsky S.J., Chalepakis G., Anthousi A., Lynd A. (2016). Cytochrome P450 associated with insecticide resistance catalyzes cuticular hydrocarbon production in *Anopheles gambiae*. Proc. Natl. Acad. Sci. USA.

[62] Wei Z., Ortiz-Urquiza A., Keyhani N.O. (2021). Altered expression of chemosensory and odorant binding proteins in response to fungal infection in the red imported fire ant, *Solenopsis invicta*. Front. Physiol..

[63] Qi Z., Gu T., Li W., Deng J., Zhang Q.H., Wickham J.D., Bai J., Zhang L. (2026). Chemosensory proteins-mediated immune response to *Beauvaria bassiana* infection in *Monochamus alternatus*. Insect Sci..

[64] Xu H., Yan K., Ding Y., Lv Y., Li J., Yang F., Chen X., Gao X., Pan Y., Shang Q. (2022). Chemosensory proteins are associated with thiamethoxam and spirotetramat tolerance in *Aphis gossypii* Glover. Int. J. Mol. Sci..

[65] Gao P., Zhang S., Tan J.J., Li X., Chen M. (2023). Chemosensory proteins are associated with thiamethoxam tolerance in bird cherry-oat aphid *Rhopalosiphum padi*. Pestic. Biochem. Physiol..

[66] Xu H., Pan Y., Li J., Yang F., Chen X., Gao X., Wen S., Shang Q. (2022). Chemosensory proteins confer adaptation to the ryanoid anthranilic diamide insecticide cyantraniliprole in *Aphis gossypii* glover. Pestic. Biochem. Physiol..

[67] Liu G., Xuan N., Chu D., Xie H.Y., Fan Z.X., Bi Y.P., Picimbon J.F., Qin Y.C., Zhong S.T., Li Y.F. (2014). Biotype expression and insecticide response of *Bemisia tabaci* chemosensory protein-1. Arch. Insect Biochem. Physiol..

[68] Kingsolver M.B., Huang Z., Hardy R.W. (2013). Insect antiviral innate immunity: Pathways, effectors, and connections. J. Mol. Biol..

[69] Hillyer J.F. (2016). Insect immunology and hematopoiesis. Dev. Comp. Immunol..

[70] Duarte-Mata D.I., Salinas-Carmona M.C. (2023). Antimicrobial peptides’ immune modulation role in intracellular bacterial infection. Front. Immunol..

[71] Mahanta D.K., Bhoi T.K., Komal J., Samal I., Nikhil R.M., Paschapur A.U., Singh G., Kumar P.V.D., Desai H.R., Ahmad M.A. (2023). Insect-pathogen crosstalk and the cellular-molecular mechanisms of insect immunity: Uncovering the underlying signaling pathways and immune regulatory function of non-coding RNAs. Front. Immunol..

[72] Wang T., Lv H., Zheng C., Yang C., Huang Y., Li X., Li J., Ma K. (2025). Overexpression of multiple odorant binding and chemosensory protein genes contributed to multi-insecticide resistance in *Aphis gossypii* Glover. Ecotoxicol. Environ. Saf..

[73] Wilkins R.M. (2017). Insecticide resistance and intracellular proteases. Pest Manag. Sci..

[74] Verdú J.R., Cortez V., Ortiz A.J., Lumaret J.P., Lobo J.M., Sánchez-Piñero F. (2020). Biomagnification and body distribution of ivermectin in dung beetles. Sci. Rep..

[75] Takaku Y., Shiraki K., Suzuki C., Takehara S., Nishii H., Sasaki T., Hariyama T. (2023). Route of pesticide spread on the body surface of *Blattella germanica* (Linnaeus): A NanoSuit-energy dispersive X-ray spectroscopy analysis. Sci. Rep..

[76] Siddiqui J.A., Khan M.M., Bamisile B.S., Hafeez M., Qasim M., Rasheed M.T., Rasheed M.A., Ahmad S., Shahid M.I., Xu Y. (2022). Role of insect gut microbiota in pesticide degradation: A review. Front. Microbiol..

[77] Wang H., Zhao R., Gao J., Xiao X., Yin X., Hu S., Zhang Y., Liang P., Gu S. (2024). Two cuticle-enriched chemosensory proteins confer multi-insecticide resistance in *Spodoptera frugiperda*. Int. J. Biol. Macromol..

[78] Wu H., Xu Q., Ma Y., Zhao X., Zhang J., Zhang Y. (2026). Two integument-enriched chemosensory proteins contribute to deltamethrin tolerance through cuticular binding in *Locusta migratoria*. Pestic. Biochem. Physiol..

[79] Yi X., Qi J., Zhou X., Hu M.Y., Zhong G.H. (2017). Differential expression of chemosensory-protein genes in midguts in response to diet of *Spodoptera litura*. Sci. Rep..

[80] Martín-Blázquez R., Chen B., Kang L., Bakkali M. (2018). Evolution, expression and association of the chemosensory protein genes with the outbreak phase of the two main pest locusts. Sci. Rep..

[81] Kodaczuk J., Sulek M., Mak P., Fraczek A., Wojda I. (2025). Chemosensory protein 16 has an immune function and participates in host-pathogen interaction in *Galleria mellonella* infected with *Pseudomonas entomophila*. Virulence.

[82] Santovito A., Audisio M., Bonelli S. (2020). A micronucleus assay detects genotoxic effects of herbicide exposure in a protected butterfly species. Ecotoxicology.

[83] Chen L.P., Jiang H.Q., Luo L., Qiu J., Xing X.J., Hou R.Y., Wu Y.J. (2023). The role of intercellular junction proteins in the penetration resistance of *Drosophila* larvae to avermectin. Comp. Biochem. Physiol. C Toxicol. Pharmacol..

[84] Huang J., Sun W., Seong K.M., Mittapalli O., Ojo J., Coates B., Paige K.N., Clark J.M., Pittendrigh B.R. (2020). Dietary antioxidant vitamin C influences the evolutionary path of insecticide resistance in *Drosophila melanogaster*. Pestic. Biochem. Physiol..

[85] Serrato-Salas J., Gendrin M. (2023). Involvement of microbiota in insect physiology: Focus on B vitamins. mBio.

[86] Deng M., Xiao T., Xu X., Wang W., Yang Z., Lu K. (2024). Nicotinamide deficiency promotes imidacloprid resistance via activation of ROS/CncC signaling pathway-mediated UGT detoxification in *Nilaparvata lugens*. Sci. Total Environ..

[87] Hu J., Rao W., Chen F., Zhou X., Wang J., Lin L., Fan G. (2024). The altered lipid composition and key lipid metabolic enzymes in thiacloprid-resistant *Myzus persicae*, with special attention paid to the function of *MpTHEM6a*. Int. J. Mol. Sci..

[88] Tsouri A., Douris V. (2025). The role of chemosensory proteins in insecticide resistance: A review. Insects.

[89] Li F., Venthur H., Wang S., Homem R.A., Zhou J.J. (2021). Evidence for the involvement of the chemosensory protein AgosCSP5 in resistance to insecticides in the cotton aphid, *Aphis gossypii*. Insects.

[90] Peng X., Qu M.J., Wang S.J., Huang Y.X., Chen C., Chen M.H. (2021). Chemosensory proteins participate in insecticide susceptibility in *Rhopalosiphum padi,* a serious pest on wheat crops. Insect Mol. Biol..

[91] Li Y., Ni S., Wang Y., Li R., Sun H., Ye X., Tian Z., Zhang Y., Liu J. (2023). The chemosensory protein 1 contributes to indoxacarb resistance in *Plutella xylostella* (L.). Pest Manag. Sci..

[92] Yao Q., Liang Z., Chen B. (2023). Evidence for the participation of chemosensory proteins in response to insecticide challenge in *Cnopomorpha sinensis*. J. Agric. Food Chem..

[93] Yao Y.J., Yin N.N., Pu L.M., Yang A.J., Liu N.Y. (2024). Three chemosensory proteins enriched in antennae and tarsi of *Rhaphuma horsfieldi* differentially contribute to the binding of insecticides. Pestic. Biochem. Physiol..

[94] Li F., Venthur H., Lin K., Zhang C., Chen Z., Zhou J.J. (2025). Insecticide chemosensory proteins as targets in insecticide resistance and development. New Plant Prot..

[95] Volonté M., Traverso L., Sierra I., Aptekmann A.A., Nadra A.D., Ons S. (2025). Characterization of odorant binding and chemosensory protein families in the kissing bug *Triatoma infestans*: Comparative analysis among Heteroptera species. BMC Genom..

[96] Wang Q., Shentu X., Yu X., Liu Y. (2025). Insect odorant-binding proteins (OBPs) and chemosensory proteins (CSPs): Mechanisms and research perspectives in mediating insecticide resistance. Biology.

[97] Cook M.E., Judd T.E. (2026). Intake regulation of vitamins C, B_1_, and B_3_ in the eastern subterranean termites, *Reticulitermes flavipes* (Blattodea: Heterotermitidae). Ann. Entomol. Soc. Am..

[98] Denecke S., Swevers L., Douris V., Vontas J. (2018). How do oral insecticidal compounds cross the insect midgut epithelium?. Insect Biochem. Mol. Biol..

[99] Frkic R.L., Giang A., Pulsford S.B., Liu J.W., Esmaeily M., Carr P.D., Fraser N.J., Hopkins D., Oakeshott J.G., Batterham P. (2026). Structural and evolutionary constraints of organophosphate resistance in dipteran carboxylesterases. Proc. Natl. Acad. Sci. USA.

[100] Maya-Aguirre C.A., Torres A., Gutiérrez-Castañeda L.D., Salazar L.M., Abreu-Villaça Y., Manhães A.C., Arenas N.E. (2024). Changes in the proteome of *Apis mellifera* acutely exposed to sublethal dosage of glyphosate and imidacloprid. Environ. Sci. Pollut. Res. Int..

[101] da Luz Scheffer J., Lima Y.S., de Castro Lippi I.C., Kadri S.M., Alvarez M.V.N., de Oliveira Orsi R. (2026). Glyphosate exposure induces transcriptomic changes in the head of Africanized *Apis mellifera* honey bees. J. Apic. Res..

[102] Picimbon J.F. (2020). Chapter three—Bioinformatic, genomic and evolutionary analysis of genes: A case study in Dipteran CSPs. Methods Enzymol..

[103] Bian H.X., Chen B.D., Zheng X.X., Ma H.F., Li Y.P., Li Q., Xia R.X., Wang H., Jiang Y.R., Liu Y.Q. (2019). Transcriptomic analysis of the prothoracic gland from two lepidopteran insects, domesticated silkmoth *Bombyx mori* and wild silkmoth *Antheraea pernyi*. Sci. Rep..

[104] Flatt T., Heyland A., Rus F., Porpiglia E., Sherlock C., Yamamoto R., Garbuzov A., Palli S.R., Tatar M., Silverman N. (2008). Hormonal regulation of the humoral innate immune response in *Drosophila melanogaster*. J. Exp. Biol..

[105] Ji Y., Gao B., Zhao D., Zhang L., Wu H., Xie Y., Shi Q., Wang Y., Guo W. (2025). The role of 20-hydroxyecdysone and juvenile hormone in insecticidal activity of *Bacillus thuringiensis* regulated by DUOX-ROS immunity in *Spodoptera exigua*. Pestic. Biochem. Physiol..

[106] Chen C.H., Pan J., Di Y.Q., Liu W., Hou L., Wang J.X., Zhao X.F. (2017). Protein kinase C delta phosphorylates ecdysone receptor B1 to promote gene expression and apoptosis under 20-hydroxyecdysone regulation. Proc. Natl. Acad. Sci. USA.

[107] Liu P., Peng H.J., Zhu J. (2015). Juvenile hormone-activated phospholipase C pathway enhances transcriptional activation by the methoprene-tolerant protein. Proc. Natl. Acad. Sci. USA.

[108] Ojani R., Liu P., Fu X., Zhu J. (2016). Protein kinase C modulates transcriptional activation by the juvenile hormone receptor methoprene-tolerant. Insect Biochem. Mol. Biol..

[109] Wang X., Hou Y., Saha T.T., Pei G., Raikhel A.S., Zou Z. (2017). Hormone and receptor interplay in the regulation of mosquito lipid metabolism. Proc. Natl. Acad. Sci. USA.

[110] Schmitz-Peiffer C. (2013). The tail wagging the dog—Regulation of lipid metabolism by protein kinase C. FEBS J..

[111] Ji Y., Wen J., Sun S., Bai B., Gao B. (2026). Phospholipase Cβ regulates midgut homeostasis and defends against *Bacillus thuringiensis* in *Spodoptera exigua*. Pestic. Biochem. Physiol..

[112] Ahmed S., Hrithik M.T.H., Roy M.C., Bode H., Kim Y. (2022). Phurealipids, produced by the entomopathogenic bacteria, *Photorhabdus*, mimic juvenile hormone to suppress insect immunity and immature development. J. Invertebr. Pathol..

[113] Kim I.H., Pham V., Jablonka W., Goodman W.G., Ribeiro J.M.C., Andersen J.F. (2017). A mosquito hemolymph odorant-binding protein family member specifically binds juvenile hormone. J. Biol. Chem..

[114] Gáliková M., Diesner M., Klepsatel P., Hehlert P., Xu Y., Bickmeyer I., Predel R., Kühnlein R.P. (2015). Energy homeostasis control in *Drosophila* adipokinetic hormone mutants. Genetics.

[115] Toprak U. (2020). The role of peptide hormones in insect lipid metabolism. Front. Physiol..

[116] Tan Q.M., Chen W.W., Li H.H., Liao S.C., Yi G.Q., Mei Y., Luo J., Tan H.H., Li X.S. (2022). Adipokinetic hormone signaling regulates cytochrome P450-mediated chlorantraniliprole sensitivity in *Spodoptera frugiperda* (Lepidoptera: Noctuidae). Pest Manag. Sci..

[117] Noriega F.G. (2014). Juvenile hormone biosynthesis in insects: What is new, what do we know, and what questions remain?. Int. Sch. Res. Not..

[118] Gao Y., Su S.C., Xing J.Y., Liu Z.Y., Nässel D.R., Bass C., Gao C., Wu S.F. (2025). Pesticide-induced resurgence in brown planthoppers is mediated by action on a suite of genes that promote juvenile hormone biosynthesis and female fecundity. eLife.

[119] Ozaki M., Wada-Katsumata A., Fujikawa K., Iwasaki M., Yokohari F., Satoji Y., Nisimura T., Yamaoka R. (2005). Ant nestmate and non-nestmate discrimination by a chemosensory sensillum. Science.

[120] Santos A.L., Preta G. (2018). Lipids in the cell: Organisation regulates function. Cell. Mol. Life Sci..

[121] Hasan M.A., Ahmed S., Kim Y. (2019). Biosynthetic pathway of arachidonic acid in *Spodoptera exigua* in response to bacterial challenge. Insect Biochem. Mol. Biol..

[122] Vatanparast M., Ahmed S., Lee D.H., Hwang S.H., Hammock B., Kim Y. (2020). EpOMEs act as immune suppressors in a lepidoptera insect, *Spodoptera exigua*. Sci. Rep..

[123] Malcicka M., Visser B., Ellers J. (2018). An evolutionary perspective on linoleic acid synthesis in animals. Evol. Biol..

[124] Reddy G.V., Guerrero A. (2004). Interactions of insect pheromones and plant semiochemicals. Trends Plant Sci..

[125] Wang F.E., Delannay C., Goindin D., Deng L., Guan S., Lu X., Fouque F., Vega-Rúa A., Picimbon J.F. (2019). Cartography of odor chemicals in the dengue vector mosquito (*Aedes aegypti* L., Diptera/Culicidae). Sci. Rep..

[126] Ebrahim S.A.M., Dweck H.K.M., Weiss B.L., Carlson J.R. (2023). A volatile sex attractant of tsetse flies. Science.

[127] Herrera H., Barros-Parada W., Bergmann J. (2019). Linoleic acid and stearic acid are biosynthetic precursors of (7Z,10Z)-7,10-hexadecadienal, the major component of the sex pheromone of *Chilecomadia valdiviana* (Lepidoptera: Cossidae). PLoS ONE.

[128] Sun Q., Haynes K.F., Zhou X. (2018). Managing the risks and rewards of death in eusocial insects. Philos. Trans. R. Soc. Lond. B Biol. Sci..

[129] Davis H.E., Meconcelli S., Radek R., McMahon D.P. (2018). Termites shape their collective behavioural response based on stage of infection. Sci. Rep..

[130] Shibao H., Kutsukake M., Matsuyama S., Fukatsu T. (2022). Linoleic acid as corpse recognition signal in a social aphid. Zool. Lett..

[131] Buehlmann C., Graham P., Hansson B.S., Knaden M. (2014). Desert ants locate food by combining high sensitivity to food odors with extensive crosswind runs. Curr. Biol..

[132] Yin C., Shan G., Guo D., Wang S., Ma X., Xiao H., Liu J., Zhang Z., Liu Y., Zhang Y. (2016). InsectBase: A resource for insect genomes and transcriptomes. Nucleic Acids Res..

[133] Swofford D.L. (2002). PAUP*. Phylogenetic Analysis Using Parsimony (* and Other Methods).

[134] Sáez A., Morales C.L., Ramos L.Y., Aizen M.A. (2014). Extremely frequent bee visits increase pollen decomposition but reduce drupelet set in raspberry. J. Appl. Ecol..

[135] Jacobsen D.J., Raguso R.A. (2018). Lingering effects of herbivory and plant defenses on pollinators. Curr. Biol..

[136] Hu S., Dilcher D.L., Jarzen D.M., Winship Taylor D. (2008). Early steps of angiosperm pollinator coevolution. Proc. Natl. Acad. Sci. USA.

[137] Zeng Y., Merchant A., Wu Q., Wang S., Kong L., Zhou X., Xie W., Zhang Y. (2020). A chemosensory protein BtabCSP11 mediates reproduction in *Bemisia tabaci*. Front. Physiol..

[138] Liu A., Li D.Z., Yang R.N., Yang M.J., Abdelnabby H., Wang M.Q. (2026). The GRP94/LMAN1-MCFD2 axis controls secretion of chemosensory proteins and enables a carboxylesterase complex in *Nilaparvata lugens*. Pest Manag. Sci..

[139] Maynard J.C., Pham T., Zheng T., Jockheck-Clark A., Rankin H.B., Newgard C.B., Spana E.P., Nicchitta C.V. (2010). Gp93, the *Drosophila* GRP94 ortholog, is required for gut epithelial homeostasis and nutrient assimilation-coupled growth control. Dev. Biol..

[140] Liu G., Yue S., Rajashekar B., Picimbon J.F. (2019). Expression of chemosensory protein (CSP) structures in *Pediculus humanus corporis* and *Acinetobacter baumannii*. SOJ Microbiol. Infect. Dis..

[141] Nguyen L.T., Schmidt H.A., von Haeseler A., Minh B.Q. (2015). IQ-TREE: A fast and effective stochastic algorithm for estimating maximum likelihood phylogenies. Mol. Biol. Evol..

[142] Chenna R., Sugawara H., Koike T., Lopez R., Gibson T.J., Higgins D.G., Thompson J.D. (2003). Multiple sequence alignment with the Clustal series of programs. Nucleic Acids Res..

[143] Smirnov V., Warnow T. (2021). Phylogeny estimation given sequence length heterogeneity. Syst. Biol..

[144] Kinene T., Wainaina J., Maina S., Boykin L.M. (2016). Rooting Trees, Methods for. Encyclopedia of Evolutionary Biology.

[145] Wilharm G., Skiebe E., Michalska A., Higgins P.G., Weber K., Schaudinn C., Neugebauer C., Görlitz K., Meimers G., Rizova Y. (2026). *Acinetobacter baumannii*’s lifestyle includes soil-dwelling colonization of decaying plant material and airborne spread. Nat. Commun..

[146] Moon S.Y., Zheng Y. (2003). Rho GTPase-activating proteins in cell regulation. Trends Cell Biol..

[147] Gouin E., Egile C., Dehoux P., Villiers V., Adams J., Gertler F., Li R., Cossart P. (2004). The RickA protein of *Rickettsia conorii* activates the Arp2/3 complex. Nature.

[148] Cronmiller E., Toor D., Shao N.C., Kariyawasam T., Wang M.H., Lee J.H. (2019). Cell wall integrity signaling regulates cell wall-related gene expression in *Chlamydomonas reinhardtii*. Sci. Rep..

[149] Boissan M., Schlattner U., Lacombe M.L. (2018). The NDPK/NME superfamily: State of the art. Lab. Investig..

[150] Sridharan Iyer S., Wu J., Pollard T.D., Voth G.A. (2025). Molecular mechanism of Arp2/3 complex activation by nucleation-promoting factors and an actin monomer. Proc. Natl. Acad. Sci. USA.

[151] Breer H., Fleischer J., Pregitzer P., Krieger J., Picimbon J.F. (2019). Molecular mechanisms of insect olfaction: Olfactory receptors. Olfactory Concepts of Insect Control-Alternative to Insecticides.

[152] Picimbon J.F., Picimbon J.F. (2019). Evolution of protein physical structures in insect chemosensory systems. Olfactory Concepts of Insect Control-Alternative to Insecticides.

[153] Ribeiro J.M., Martin-Martin I., Arcà B., Calvo E. (2016). A Deep insight into the sialome of male and female *Aedes aegypti* mosquitoes. PLoS ONE.

[154] Yadav K., Rana V.S., Anjali, Saurav G.K., Rawat N., Kumar A., Sunil S., Singh O.P., Rajagopal R. (2023). Mucin protein of *Aedes aegypti* interacts with Dengue Virus 2 and influences viral infection. Microbiol. Spectr..

[155] Ho S. (2008). The molecular clock and estimating species divergence. Nat. Educ..

[156] Schachat S.R., Goldstein P.Z., Desalle R., Bobo D.M., Boyce K., Payne J.L., Labandeira C.C. (2023). Illusion of flight? Absence, evidence and the age of winged insects. Biol. J. Linn. Soc..

[157] Cornman R.S. (2010). The distribution of GYR- and YLP-like motifs in *Drosophila* suggests a general role in cuticle assembly and other protein-protein interactions. PLoS ONE.

[158] Teparić R., Lozančić M., Mrša V. (2020). Evolutionary overview of molecular interactions and enzymatic activities in the yeast cell walls. Int. J. Mol. Sci..

[159] Adachi N., Senda T., Horikoshi M. (2016). Uncovering ancient transcription systems with a novel evolutionary indicator. Sci. Rep..

[160] Akil C., Kitaoku Y., Tran L.T., Liebl D., Choe H., Muengsaen D., Suginta W., Schulte A., Robinson R.C. (2021). Mythical origins of the actin cytoskeleton. Curr. Opin. Cell Biol..

[161] Rodriguez-Oliveira T., Wollweber F., Ponce-Toledo R.I., Xu J., Rittmann S.K.-M.R., Klingl A., Pilhofer M., Schleper C. (2023). Actin cytoskeleton and complex cell architecture in an *Asgard archaeon*. Nature.

[162] Charles-Orszag A., Petek-Seoane N.A., Mullins R.D. (2024). Archaeal actins and the origin of a multifunctional cytoskeleton. J. Bacteriol..

[163] Waterhouse A., Bertoni M., Bienert S., Studer G., Tauriello G., Gumienny R., Heer F.T., de Beer T.A.P., Rempfer C., Bordoli L. (2018). SWISS-MODEL: Homology modelling of protein structures and complexes. Nucleic Acids Res..

[164] Ho H.-Y.H., Rohatgi R., Ma L., Kirschner M.W. (2001). CR16 forms a complex with N-WASP in brain and is a novel member of a conserved proline-rich actin-binding protein family. Proc. Natl. Acad. Sci. USA.

[165] Chereau D., Kerff F., Graceffa P., Grabarek Z., Langsetmo K., Dominguez R. (2005). Actin-bound structures of Wiskott-Aldrich syndrome protein (WASP)-homology domain 2 and the implications for filament assembly. Proc. Natl. Acad. Sci. USA.

[166] Rogerio A.P., Anibal F.F. (2012). Role of leukotrienes on protozoan and helminth infections. Mediat. Inflamm..

[167] Stanley D., Kim Y. (2019). Chapter Eight—Insect prostaglandins and other eicosanoids: From molecular to physiological actions. Adv. Insect Physiol..

[168] Roy M.C., Nam K., Kim J., Stanley D., Kim Y. (2021). Thromboxane mobilizes insect blood cells to infection foci. Front. Immunol..

[169] El-Yassimi A., Hichami A., Besnard P., Khan N.A. (2008). Linoleic acid induces calcium signaling, Src kinase phosphorylation, and neurotransmitter release in mouse CD36-positive gustatory cells. J. Biol. Chem..

[170] Feng D.D., Zhao Y.F., Luo Z.Q., Keating D.J., Chen C. (2008). Linoleic acid induces Ca2+-induced inactivation of voltage-dependent Ca2+ currents in rat pancreatic β-cells. J. Endocrinol..

[171] Clements J., Groves R.L., Cava J., Barry C.C., Chapman S., Olson J.M. (2019). Conjugated linoleic acid as a novel insecticide targeting the agricultural pest *Leptinotarsa decemlineata*. PLoS ONE.

[172] Macháček S., Tupec M., Horáček N., Halmová M., Roy A., Machara A., Kyjaková P., Lukšan O., Pichová I., Hanus R. (2023). Evolution of linoleic acid biosynthesis paved the way for ecological success of termites. Mol. Biol. Evol..

[173] Carpentier J., Abenaim L., Luttenschlager H., Dessauvages K., Liu Y., Samoah P., Francis F., Caparros Megido R. (2024). Microorganism contribution to mass-reared edible insects: Opportunities and challenges. Insects.

[174] Arien Y., Dag A., Zarchin S., Masci T., Shafir S. (2015). Omega-3 deficiency impairs honey bee learning. Proc. Natl. Acad. Sci. USA.

[175] Vesala L., Basikhina Y., Tuomela T., Nurminen A., Siukola E., Vale P.F., Salminen T.S. (2024). Mitochondrial perturbation in immune cells enhances cell-mediated innate immunity in *Drosophila*. BMC Biol..

[176] Wrońska A.K., Kaczmarek A., Boguś M.I., Kuna A. (2023). Lipids as a key element of insect defense systems. Front. Genet..

[177] Mercola J. (2025). Linoleic acid, mitochondria, gut microbiome, and metabolic health: A mechanistic review. Adv. Redox Res..

[178] Giblin A., Cammack A.J., Blomberg N., Anoar S., Mikheenko A., Carcolé M., Atilano M.L., Hull A., Shen D., Wei X. (2025). Neuronal polyunsaturated fatty acids are protective in ALS/FTD. Nat. Neurosci..

[179] Marmunti M., Catalá A. (2001). Incorporation of 1-(14)C linoleic acid in rat liver nuclei and chromatin fractions. Int. J. Biochem. Cell Biol..

[180] Ando H., Wen Z.M., Kim H.Y., Valencia J.C., Costin G.E., Watabe H., Yasumoto K., Niki Y., Kondoh H., Ichihashi M. (2006). Intracellular composition of fatty acid affects the processing and function of tyrosinase through the ubiquitin-proteasome pathway. Biochem. J..

[181] Masner M., Lujea N., Bisbal M., Acosta C., Kunda P. (2021). Linoleic and oleic acids enhance cell migration by altering the dynamics of microtubules and the remodeling of the actin cytoskeleton at the leading edge. Sci. Rep..

[182] Savini M., Folick A., Lee Y.T., Jin F., Cuevas A., Tillman M.C., Duffy J.D., Zhao Q., Neve I.A., Hu P.W. (2022). Lysosome lipid signalling from the periphery to neurons regulates longevity. Nat. Cell Biol..

[183] Parsons J.B., Rock C.O. (2013). Bacterial lipids: Metabolism and membrane homeostasis. Prog. Lipid Res..

[184] Atilla-Gokcumen G.E., Muro E., Relat-Goberna J., Sasse S., Bedigian A., Coughlin M.L., Garcia-Manyes S., Eggert U.S. (2014). Dividing cells regulate their lipid composition and localization. Cell.

[185] Blank H.M., Perez R., He C., Maitra N., Metz R., Hill J., Lin Y., Johnson C.D., Bankaitis V.A., Kennedy B.K. (2017). Translational control of lipogenic enzymes in the cell cycle of synchronous, growing yeast cells. EMBO J..

[186] Yuan Y., Jin M., Fang F., Tocher D.R., Betancor M.B., Jiao L., Hong Y., Zhou Q. (2022). New Insight into the molting and growth in crustaceans: Regulation of energy homeostasis through the lipid nutrition. Front. Mar. Sci..

[187] Feyereisen R. (1999). Insect P450 enzymes. Annu. Rev. Entomol..

[188] Fujinaga D., Ohhara Y., Okamoto N., Chu H., Mauck K.E., Yamanaka N. (2026). Two cytochrome P450 epoxidases mediate juvenile hormone biosynthesis in *Drosophila melanogaster*. Insect Biochem. Mol. Biol..

[189] Yokoyama N., Fónagy A., Tatsuki S., Arie T., Yamashita S., Matsumoto S. (2003). Ultrastructural studies on the pheromone producing cells in the silkmoth, *Bombyx mori*: Formation of cytoplasmic lipid droplets before adult eclosion. Acta Biol. Hung..

[190] Xuan N., Bu X., Liu Y.Y., Yang X., Liu G., Fan Z.X., Bi Y.P., Yang L.Q., Lu Q.N., Rajashekar B. (2014). Molecular evidence of RNA editing in the *Bombyx* chemosensory protein family. PLoS ONE.

[191] Hull J., Fónagy A., Picimbon J.F. (2019). Molecular basis of pheromonogenesis regulation in moths. Olfactory Concepts of Insect Control-Alternative to Insecticides.

[192] Wang L., Guo P., Zhang X., Duan Y., Ning J., Zhang T., Yang X. (2025). Identification of Δ9 and Δ11 desaturases involved in sex pheromone biosynthesis in *Mythimna loreyi* (Lepidoptera: Noctuidae). J. Agric. Food Chem..

[193] Ibarra A., Hetzer M.W. (2015). Nuclear pore proteins and the control of genome functions. Genes Dev..

[194] Lin D.H., Hoelz A. (2019). The structure of the Nuclear Pore Complex (an update). Annu. Rev. Biochem..

[195] Thelen A.M., Zoncu R. (2017). Emerging roles for the lysosome in lipid metabolism. Trends Cell Biol..

[196] Kenis S., Istiban M.N., Van Damme S., Vandewyer E., Watteyne J., Schoofs L., Beets I. (2023). Ancestral glycoprotein hormone receptor pathway controls growth in *C. elegans*. Front. Endocrinol..

[197] Roger A.J., Muñoz-Gómez S.A., Kamikawa R. (2017). The origin and diversification of mitochondria. Curr. Biol..

[198] Fisher W.W., Hemp J., Johnson J.E. (2016). Evolution of oxygenic photosynthesis. Annu. Rev. Earth Planet. Sci..

[199] Dermauw W., Van Leeuwen T., Feyereisen R. (2020). Diversity and evolution of the P450 family in arthropods. Insect Biochem. Mol. Biol..

[200] Lang T., Hansson G.C., Samuelsson T. (2007). Gel-forming mucins appeared early in metazoan evolution. Proc. Natl. Acad. Sci. USA.

[201] Lang T., Klasson S., Larsson E., Johansson M.E., Hansson G.C., Samuelsson T. (2016). Searching the evolutionary origin of epithelial mucus protein components-Mucin and FCGBP. Mol. Biol. Evol..

[202] McShane A., Bath J., Jaramillo A.M., Ridley C., Walsh A.A., Evans C.M., Thornton D.J., Ribbeck K. (2021). Mucus. Curr. Biol..

[203] Syed Z.A., Hard T., Uv A., van Dijk-Hard I.F. (2008). A potential role for *Drosophila* mucins in development and physiology. PLoS ONE.

[204] Zhao X., Zhang J., Yang J., Niu N., Zhang J., Yang Q. (2020). Mucin family genes are essential for the growth and development of the migratory locust, *Locusta migratoria*. Insect Biochem. Mol. Biol..

[205] Huang Y., Li L., Rong Y.S. (2022). JiangShi (僵尸): A widely distributed Mucin-like protein essential for *Drosophila* development. G3.

[206] Lou Y.H., Shen Y., Li D.T., Huang H.J., Lu J.B., Zhang C.X. (2019). A mucin-like protein is essential for oviposition in *Nilaparvata lugens*. Front. Physiol..

[207] Ibrahim S.P., Dias R.O., Ferreira C., Silva C.P., Terra W.R. (2023). Histochemistry and transcriptomics of mucins and peritrophic membrane (PM) proteins along the midgut of a beetle with incomplete PM and their complementary function. Insect Biochem. Mol. Biol..

[208] Moriyama M., Hayashi T., Fukatsu T. (2022). A mucin protein predominantly expressed in the female-specific symbiotic organ of the stinkbug *Plautia stali*. Sci. Rep..

[209] Deng F., Wu S., Wu Y., Liu X., Wu P., Zhai Z. (2020). Identification of mucins and their expression in the vector mosquito *Aedes albopictus*. J. Vector Ecol..

[210] Taparia T., Ignell R., Hill S.R. (2017). Blood meal induced regulation of the chemosensory gene repertoire in the southern house mosquito. BMC Genom..

[211] Hill S.R., Ghaninia M., Ignell R. (2019). Blood meal induced regulation of gene expression in the maxillary palps, a chemosensory organ of the mosquito *Aedes aegypti*. Front. Ecol. Evol..

[212] Shangguan X., Zhang J., Liu B., Zhao Y., Wang H., Wang Z., Guo J., Rao W., Jing S., Guan W. (2018). A mucin-like protein of planthopper is required for feeding and induces immunity responses in plants. Plant Physiol..

[213] Slomiany B.L., Murty V.L., Sarosiek J., Piotrowski J., Slomiany A. (1988). Role of associated and covalently bound lipids in salivary mucin hydrophobicity: Effect of proteolysis and disulfide bridge reduction. Biochem. Biophys. Res. Commun..

[214] Gong D.H., Turner B., Bhaskar K.R., Lamont J.T. (1990). Lipid binding to gastric mucin: Protective effect against oxygen radicals. Am. J. Physiol.-Gastrointest. Liver Physiol..

[215] Bansil R., Turner B.S. (2018). The biology of mucus: Composition, synthesis and organization. Adv. Drug Deliv. Rev..

[216] Terra W.R., Dias R.O., Oliveira P.L., Ferreira C., Venancio T.M. (2018). Transcriptomic analyses uncover emerging roles of mucins, lysosome/secretory addressing and detoxification pathways in insect midguts. Curr. Opin. Insect Sci..

[217] Vial T., Marti G., Missé D., Pompon J. (2021). Lipid interactions between flaviviruses and mosquito vectors. Front. Physiol..

[218] Carlson T.L., Yildiz H., Dar Z., Lock J.Y., Carrier R.L. (2018). Lipids alter microbial transport through intestinal mucus. PLoS ONE.

[219] Fu L., Wang M., Li D., Ma S., Zhang F., Zheng L. (2025). Microbial metabolites short chain fatty acids, tight junction, gap junction, and reproduction: A review. Front. Cell Dev. Biol..

[220] Gouda M.N.R., Subramanian S. (2026). Odorant-binding proteins and chemosensory proteins in insects: Structural insights, functional plasticity, and prospects for targeted pest management. Mol. Biol. Rep..

