# Supplementary material for: Renaming the ‘OS-D/CSP’ Family (Part 2): ‘4-Cysteine Soluble Proteins’ (4CSPs)—Intracellular Functions
Source: Insects. 2026 Sep 8;17(9):940. doi: 10.3390/insects17090940 (PMC13607926; doi:10.3390/insects17090940)
Supplement: Supplementary file 1 [file insects-17-00940-s001.zip › FigureS1.pdf]

```
               1       10        20        30        40        50        60        70        80        90        100       110       120      130
               |--------+---------+---------+---------+---------+---------+---------+---------+---------+---------+---------+---------+---------|
Mp10           MFSTQSQAMDSRIAVVCVVLAVFAVDQTVGAPQKDAVAASGPAYTTKYDHIDIDQVLGSKRLVNSYVQCLLDKKPCTPEGAELRKILPDALKTQCVKCNATQKNAALKVVDRLQRDYDKEWKQLLDKWDP
ACYPI000097            MDSRIAVVCVVLAVFAVDQTVGAPQKDAVAASGTAYTTKYDHIDIDQVLASKRLVNSYVQCLLDKKPCTPEGAELRKILPDALKTQCAKCNATQKNAALKVVDRLQRDYDKEWKQLLDKWDP
BPHOGS10008228                           MYVPYRVGVSLCLADDQYTSKYDNIDLDKILDSKRLVSNYVQCLLGTKGCPPEGLELKRILPDALKTQCSKCTCVQRQGAVKAIQRLQKDYPDEWKLLLDKWDA
BPHOGS10002786                    MSNYIIKCVLLVSVSVSLCLADDQYTSKYDNIDLDKILDSKRLVSNYVQCLLGTKGCPPEGLELKRILPDALKTQCSKCTCVQRQGAVKAIQRLQKDYPDEWKLLLDKWDA
AAJJ0269C                             MIPLIAIAGILAVSAAPAEFYESRYDHLDVESILNNRRMVNYYAACLLSKGPCPPQGVDLKRVLPEALQTNCAKCTEKQRTAAYRSIKRLKKEYPKIWEQLRAVWDP
AAJJ0269B                     MIKRKFRFIPVHDFVNGKTLHRSTRDDKYTTRYDNVDVDRILHSKRLLLNYINCLLEKGPCSPEGRELKKILPDALVTNCSKCSEVQKKQAGKILTFVLLNYRNEWNQLVAKYDP
DP0006842                          MHCASVFFVVSALLVLISAQSSPYTSKYDNVDVDKILKNDRVLTNYIKCLMEEGPCTPEGRELRKTLPDALASGCSKCNEKQKSTTEKVIRHLQTRRAKDWDRLSKKYDP
XP_012166268                              MRTEETAQVVCVTAEDYTTKYDNVDVDRILQNGRILTNYIKCMLDEGPCTNEGRELKKILPDALSTGCSKCNEKQKHTANKVVNYLRTKRPKDWERLSAKYDS
HMEL010990                         MKTVLC--LFALVAVACALPSSTYTDKYDNIDLDEILSNKRLLTPYVKCMLDEGKCSPEGKELKSHVREALENECSKCTENQKAGSRKVIGHLINNEQEYWSKLTAKYDP
CSUOGS107535                       MKAIILIGLSVVVTIAWARPGNTYTDKWDHINVDEILESNRLLKGYVDCLLERGRCTPDGKALKETLPDALEHECSKCTEKQKTASDKVIRHLVNKRPDLWTELAAKYDP
Bta06193                          MNLLSVVVLVCCLFAAVLSAPAEFYTSQFDNIDIESILNNEKLLDNYFKCLMDEGPCTLEGRTLKSLVPDALNTSCAKCTDKQKQIARRVITFYLDKYPANSARIIKKYDP
PVENscaf20457                       KLFVLAFCLATAATVLAVPAPAYTTKYDNVNIDEILGNDRLF----------------------LPDALENDCKGCSEKQKEGAERIIKHLIDNKPDMWTKLEEKYDK
Consensus      Signal peptide ......................a.p...YT.k%Dn!#.#.IL.n.Rll..y..cll..gpc.peg.elk..lP#AL.t.CskC.ekQk..a.kv!..l...yp..W..L..KyDp
                                                                                     *      *                  *  *

               131    140       150       160       170       180       190       200       210       220       230       240       250      260
               |--------+---------+---------+---------+---------+---------+---------+---------+---------+---------+---------+---------+---------|
Mp10           KREYFQKFQQFLAEEKKKGVVKF
ACYPI000097    KREQFQKFQQFLAEEKKKGVVKF
BPHOGS10008228 KRESLAKFDDYVKKGGKIC
BPHOGS10002786 KRESLAKFDDYVKKGGKIC
AAJJ0269C      DDVFIRKFETSFESGKPSGVISTNTSPPSPILSNRFGENEEADAASNVISSTPLPPTTSTTTRTTLTTKFTTKPSTKPTNKPVVVTKPPQAPPFATVGANLQATVSFGTNLVGGIVRSLGTLGSRVVESG
AAJJ0269B      DGIYRKQYEIDDDYDYSELDSAKK
DP0006842      EGVYKQKYTAELKTETTA
XP_012166268   SGEYKKRYENVLQPTKNS
HMEL010990     DQKYAKKYEKELKTVSA
CSUOGS107535   DNMYQQRYKDKIEEVKGKQ
Bta06193       ENKFKDGIEKALLGSR
PVENscaf20457  TG
Consensus      ........................

               261     270 274
               |--------+---|
Mp10
ACYPI000097
BPHOGS10008228
BPHOGS10002786
AAJJ0269C      TKLANMVISAAIRP
AAJJ0269B
DP0006842
XP_012166268
HMEL010990
CSUOGS107535
Bta06193
PVENscaf20457
Consensus
```
