# Supplementary material for: Renaming the ‘OS-D/CSP’ Family (Part 2): ‘4-Cysteine Soluble Proteins’ (4CSPs)—Intracellular Functions
Source: Insects. 2026 Sep 8;17(9):940. doi: 10.3390/insects17090940 (PMC13607926; doi:10.3390/insects17090940)
Supplement: Supplementary file 1 [file insects-17-00940-s001.zip › FigureS2.pdf]

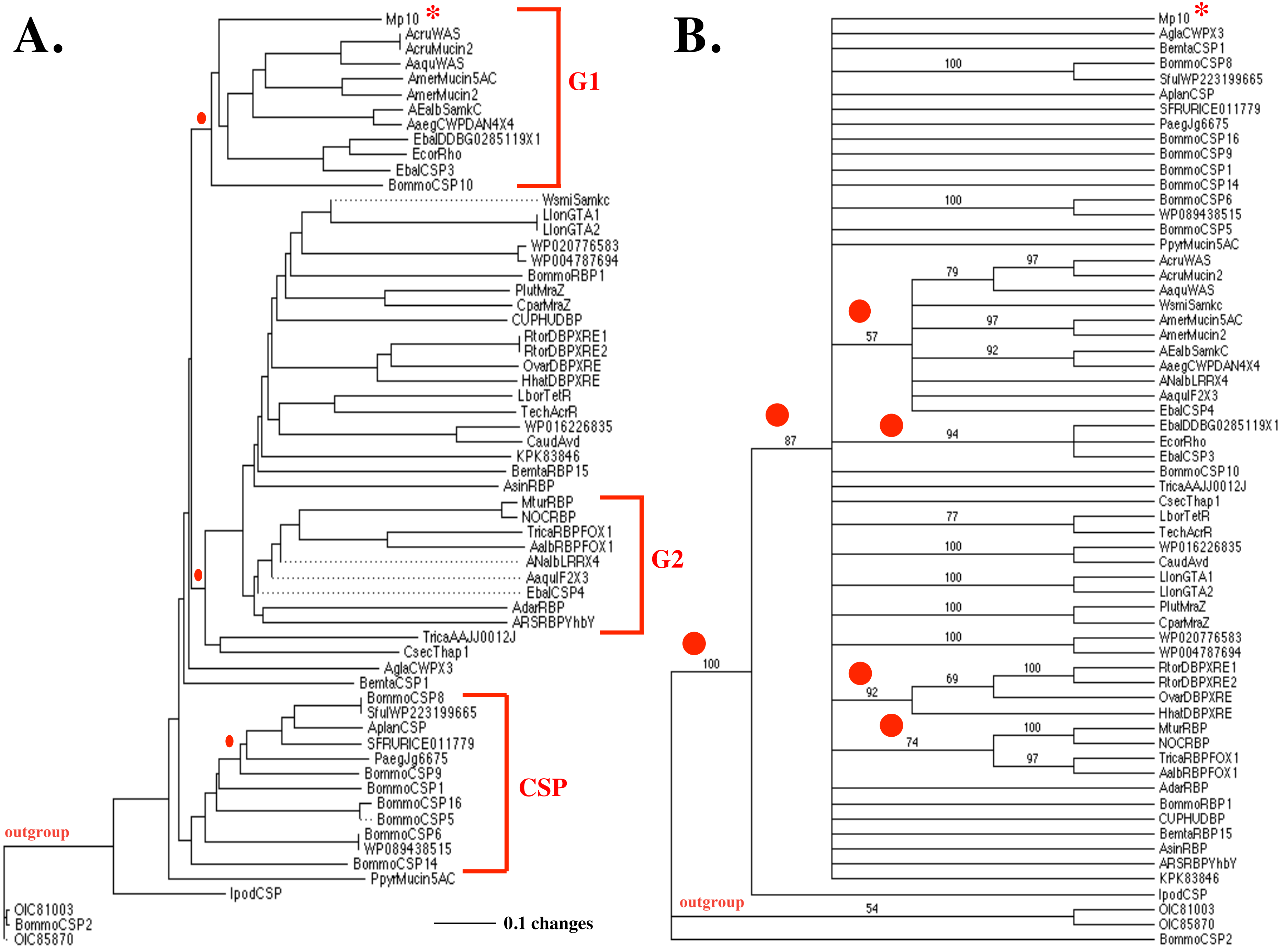
A.
Mp10 *
AcruWAS
AcruMucin2
AaquWAS
AmerMucin5AC
AmerMucin2
AEalbSamkC
AaegCWPDAN4X4
EbalDDBG0285119X1
EcorRho
EbalCSP3
BommoCSP10
G1
WsmiSamkc
LlonGTA1
LlonGTA2
WP020776583
WP004787694
BommoRBP1
PlutMraZ
CparMraZ
CUPHUDBP
RtorDBPXRE1
RtorDBPXRE2
OvarDBPXRE
HhatDBPXRE
LborTetR
TechAcrR
WP016226835
CaudAvd
KPK83846
BemtaRBP15
AsinRBP
MturRBP
NOCRBP
TricaRBPFOX1
AalbRBPFOX1
ANalbLRRX4
AaquIF2X3
EbalCSP4
AdarRBP
ARSRBPYhbY
G2
TricaAAJJ0012J
CsecThap1
AglaCWPX3
BemtaCSP1
BommoCSP8
SfulWP223199665
AplanCSP
SFRURICE011779
PaegJg6675
BommoCSP9
BommoCSP1
BommoCSP16
BommoCSP5
BommoCSP6
WP089438515
BommoCSP14
PpyrMucin5AC
CSP
IpodCSP
outgroup
OIC81003
BommoCSP2
OIC85870
0.1 changes
B.
Mp10 *
AglaCWPX3
BemtaCSP1
BommoCSP8
SfulWP223199665
AplanCSP
SFRURICE011779
PaegJg6675
BommoCSP16
BommoCSP9
BommoCSP1
BommoCSP14
BommoCSP6
WP089438515
BommoCSP5
PpyrMucin5AC
AcruWAS
AcruMucin2
AaquWAS
WsmiSamkc
AmerMucin5AC
AmerMucin2
AEalbSamkC
AaegCWPDAN4X4
ANalbLRRX4
AaquIF2X3
EbalCSP4
EbalDDBG0285119X1
EcorRho
EbalCSP3
BommoCSP10
TricaAAJJ0012J
CsecThap1
LborTetR
TechAcrR
WP016226835
CaudAvd
LlonGTA1
LlonGTA2
PlutMraZ
CparMraZ
WP020776583
WP004787694
RtorDBPXRE1
RtorDBPXRE2
OvarDBPXRE
HhatDBPXRE
MturRBP
NOCRBP
TricaRBPFOX1
AalbRBPFOX1
AdarRBP
BommoRBP1
CUPHUDBP
BemtaRBP15
AsinRBP
ARSRBPYhbY
KPK83846
IpodCSP
OIC81003
OIC85870
BommoCSP2
outgroup
100
100
97
79
57
97
92
87
94
77
100
100
100
100
100
69
92
100
74
100
97
54
