# Supplementary material for: Renaming the ‘OS-D/CSP’ Family (Part 2): ‘4-Cysteine Soluble Proteins’ (4CSPs)—Intracellular Functions
Source: Insects. 2026 Sep 8;17(9):940. doi: 10.3390/insects17090940 (PMC13607926; doi:10.3390/insects17090940)
Supplement: Supplementary file 1 [file insects-17-00940-s001.zip › FigureS4.pdf]

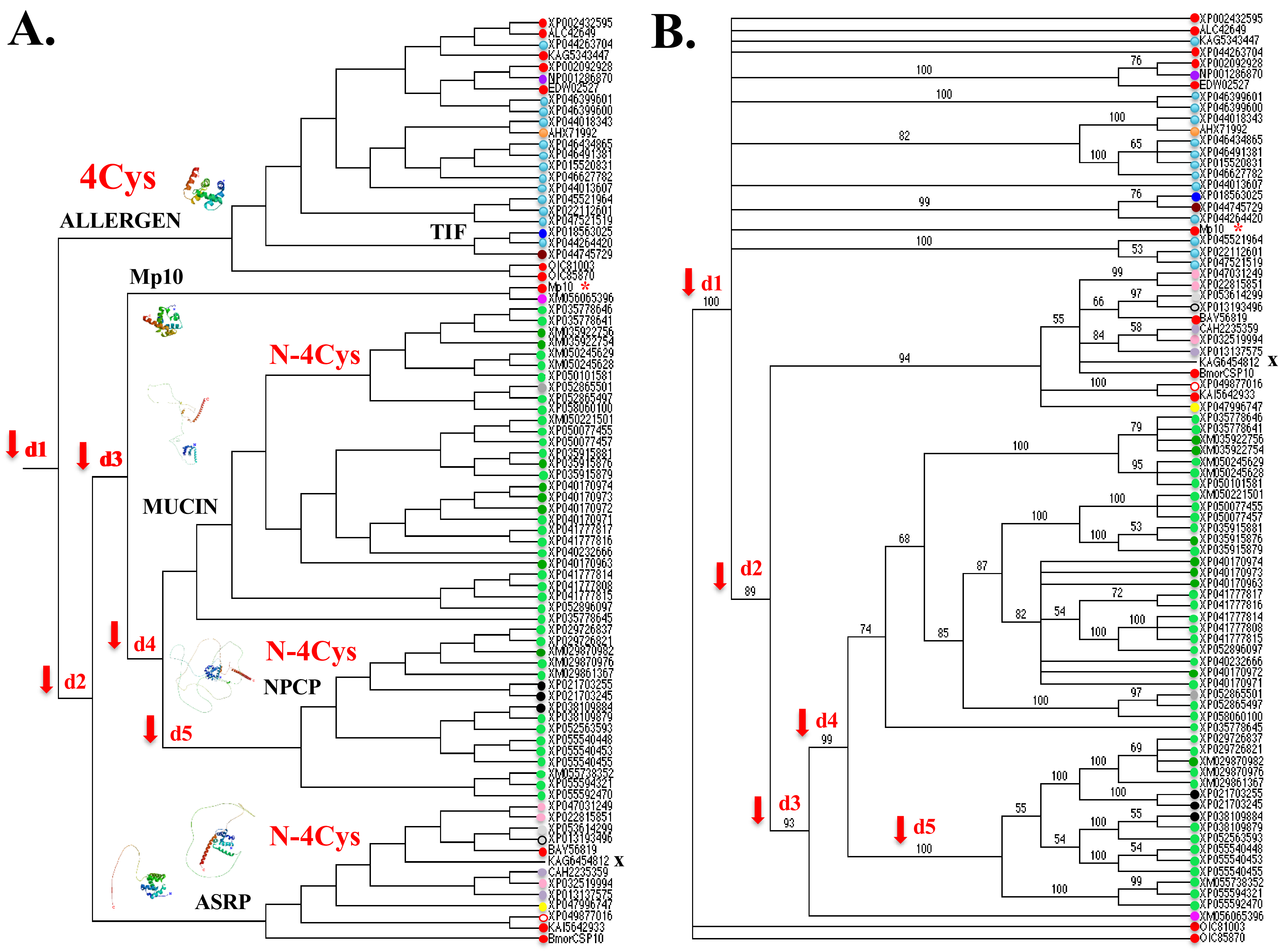

A.
4Cys
ALLERGEN
TIF
Mp10
MUCIN
N-4Cys
NPCP
N-4Cys
ASRP
d1
d2
d3
d4
d5
XP002432595
ALC42649
XP044263704
KAG5343447
XP002092928
NP001286870
EDW02527
XP046399601
XP046399600
XP044018343
AHX71992
XP046434865
XP046491381
XP015520831
XP046627782
XP044013607
XP045521964
XP022112601
XP047521519
XP018563025
XP044264420
XP044745729
OIC81003
OIC85870
Mp10 *
XM056065396
XP035778646
XP035778641
XM035922756
XM035922754
XM050245629
XM050245628
XP050101581
XP052865501
XP052865497
XP058060100
XM050221501
XP050077455
XP050077457
XP035915881
XP035915876
XP035915879
XP040170974
XP040170973
XP040170972
XP040170971
XP041777817
XP041777816
XP040232666
XP040170963
XP041777814
XP041777808
XP041777815
XP052896097
XP035778645
XP029726837
XP029726821
XM029870982
XM029870976
XM029861367
XP021703255
XP021703245
XP038109884
XP038109879
XP052563593
XP055540448
XP055540453
XP055540455
XM055738352
XP055594321
XP055592470
XP047031249
XP022815851
XP053614299
XP013193496
BAY56819
KAG6454812 x
CAH2235359
XP032519994
XP013137575
XP047996747
XP049877016
KAI5642933
BmorCSP10
B.
d1
d2
d3
d4
d5
XP002432595
ALC42649
KAG5343447
XP044263704
XP002092928
NP001286870
EDW02527
XP046399601
XP046399600
XP044018343
AHX71992
XP046434865
XP046491381
XP015520831
XP046627782
XP044013607
XP018563025
XP044745729
XP044264420
Mp10 *
XP045521964
XP022112601
XP047521519
XP047031249
XP022815851
XP053614299
XP013193496
BAY56819
CAH2235359
XP032519994
XP013137575
KAG6454812 x
BmorCSP10
XP049877016
KAI5642933
XP047996747
XP035778646
XP035778641
XM035922756
XM035922754
XM050245629
XM050245628
XP050101581
XM050221501
XP050077455
XP050077457
XP035915881
XP035915876
XP035915879
XP040170974
XP040170973
XP040170963
XP041777817
XP041777816
XP041777814
XP041777808
XP041777815
XP052896097
XP040232666
XP040170972
XP040170971
XP052865501
XP052865497
XP058060100
XP035778645
XP029726837
XP029726821
XM029870982
XM029870976
XM029861367
XP021703255
XP021703245
XP038109884
XP038109879
XP052563593
XP055540448
XP055540453
XP055540455
XM055738352
XP055594321
XP055592470
XM056065396
OIC81003
OIC85870
