# Supplementary material for: Renaming the ‘OS-D/CSP’ Family (Part 2): ‘4-Cysteine Soluble Proteins’ (4CSPs)—Intracellular Functions
Source: Insects. 2026 Sep 8;17(9):940. doi: 10.3390/insects17090940 (PMC13607926; doi:10.3390/insects17090940)
Supplement: Supplementary file 1 [file insects-17-00940-s001.zip › FigureS5.pdf]

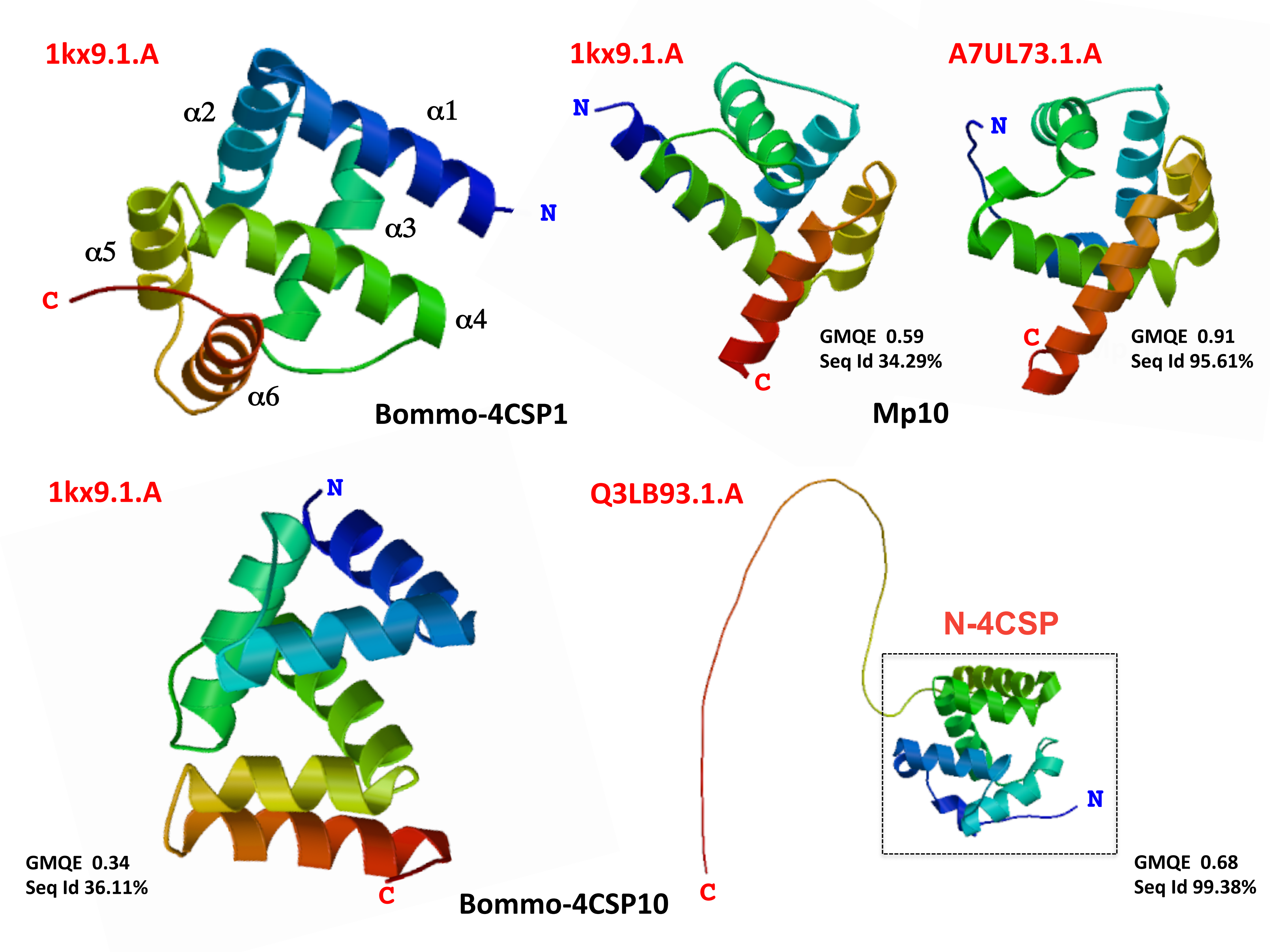

1kx9.1.A
α2
α1
N
α3
α5
C
α4
α6
Bommo-4CSP1
1kx9.1.A
N
C
GMQE 0.59
Seq Id 34.29%
A7UL73.1.A
N
C
GMQE 0.91
Seq Id 95.61%
Mp10
1kx9.1.A
N
C
GMQE 0.34
Seq Id 36.11%
Bommo-4CSP10
Q3LB93.1.A
N-4CSP
N
C
GMQE 0.68
Seq Id 99.38%

Q9W0X2.1.A

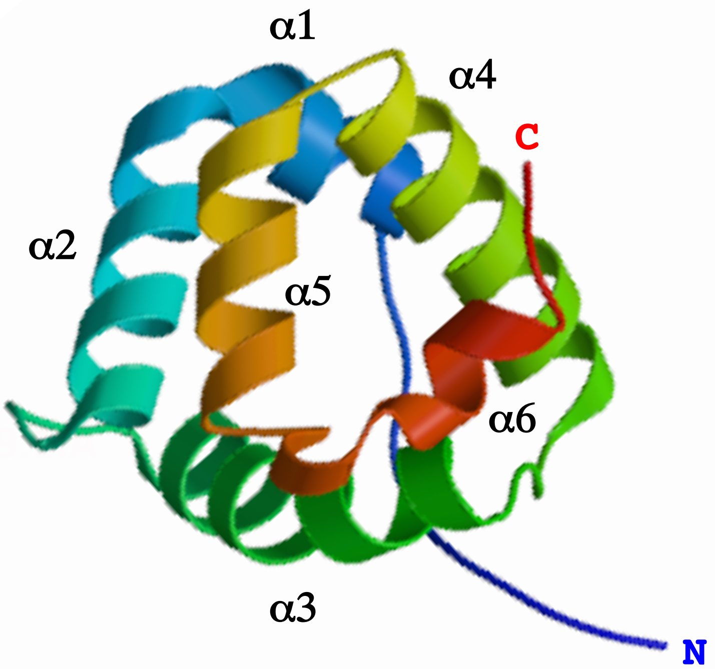

GMQE 0.93
Seq Id 79.41%

1kx9.1.A

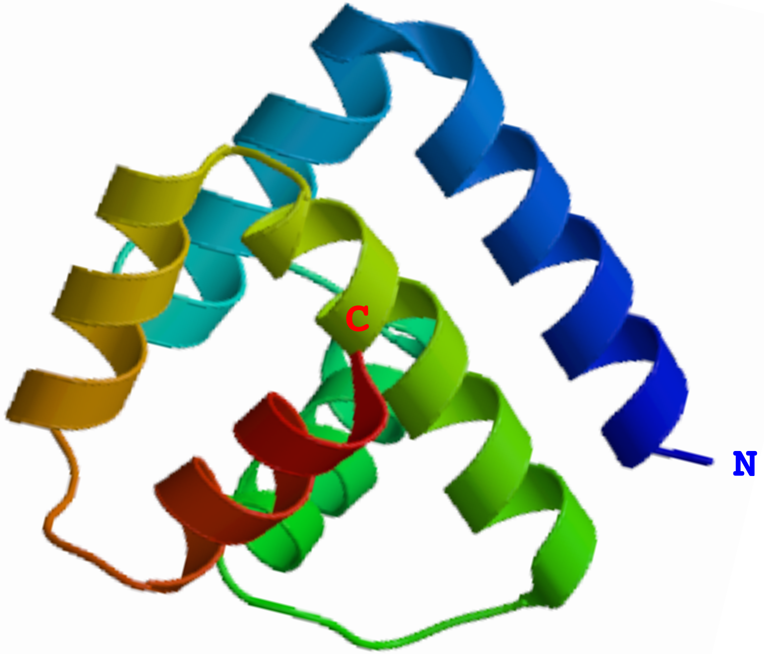

GMQE 0.66
Seq Id 46.53%

GH22042
Drosophila grimshawi
pherokine (hemolymph protein)

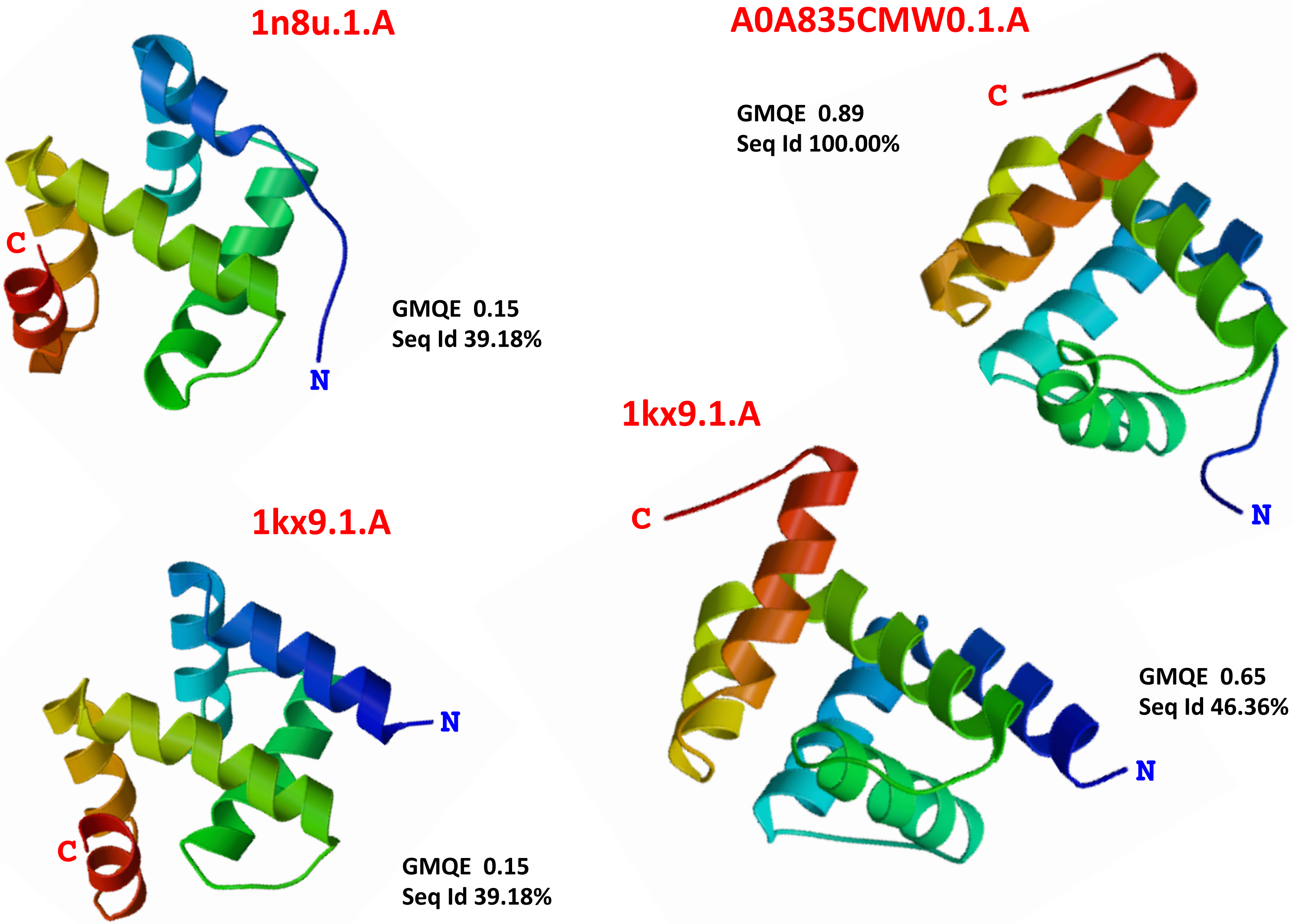

XM_044889794
Coccinella septempunctata
eukaryotic translation initiation factor 4 gamma-like

AHX71992 (KJ451426)
Aphis gossypii
acid trehalase

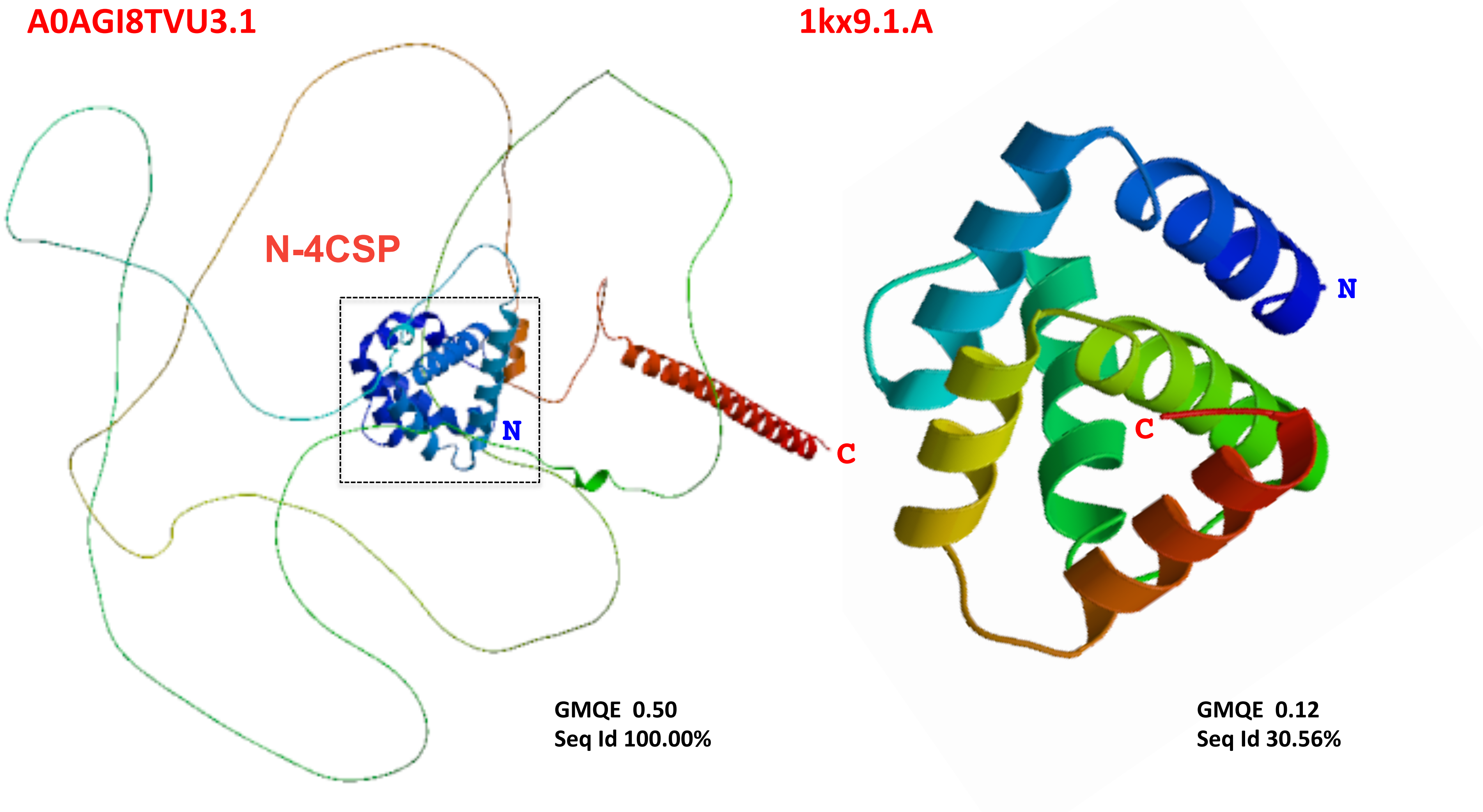

XP_021703245

Aedes aegypti

nuclear pore complex protein

(NPCP)

A0A2H4ZB78.1.A

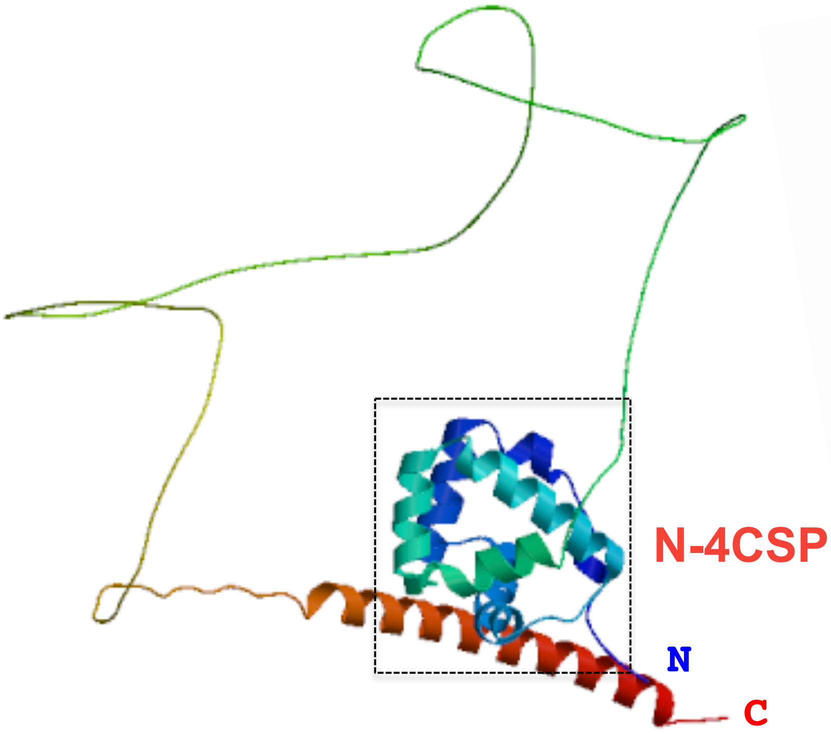

GMQE 0.62
Seq Id 97.54%

1kx9.1.A

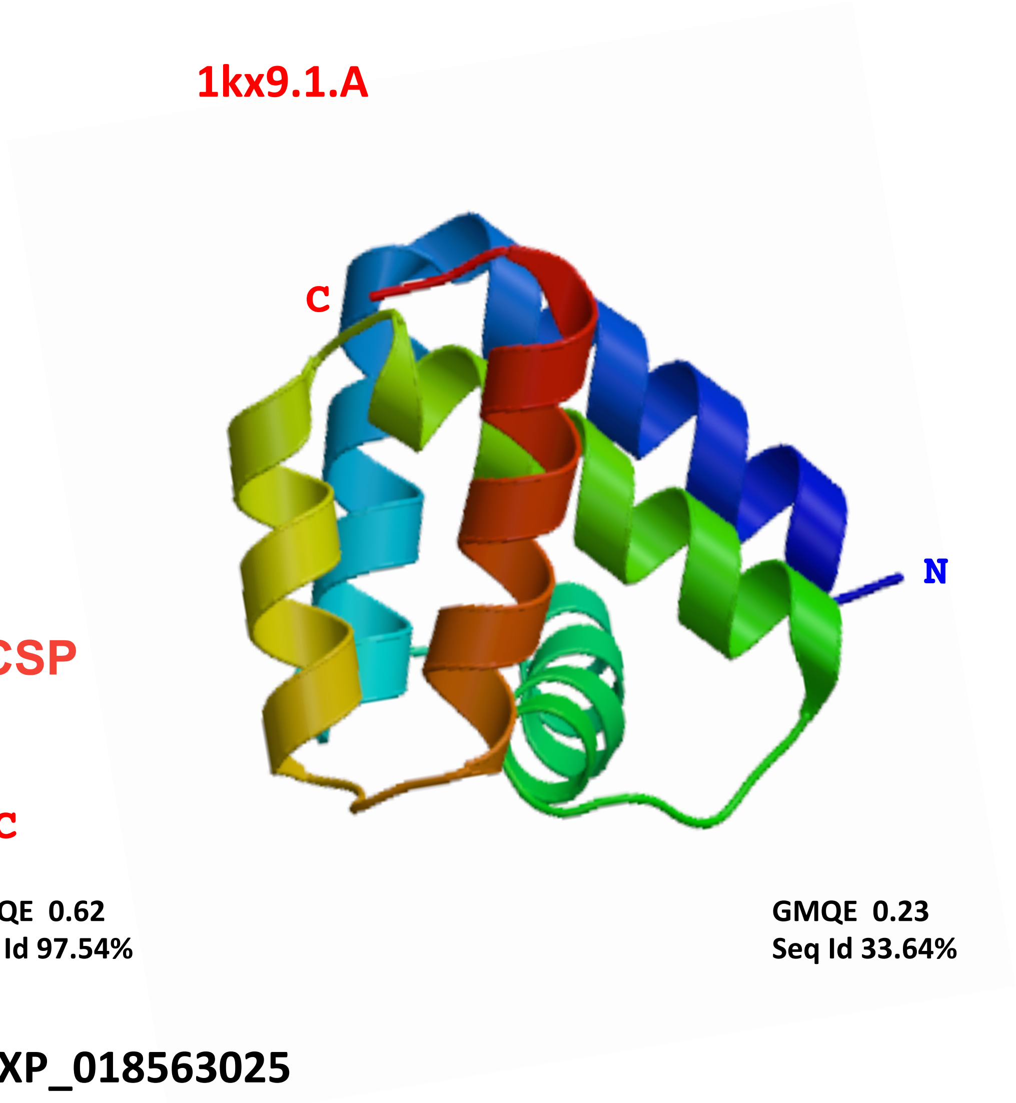

XP_018563025

Anoplophora glabripennis

cell wall integrity and stress response component 3-like (CWP)

Q6H8Y7.1.A

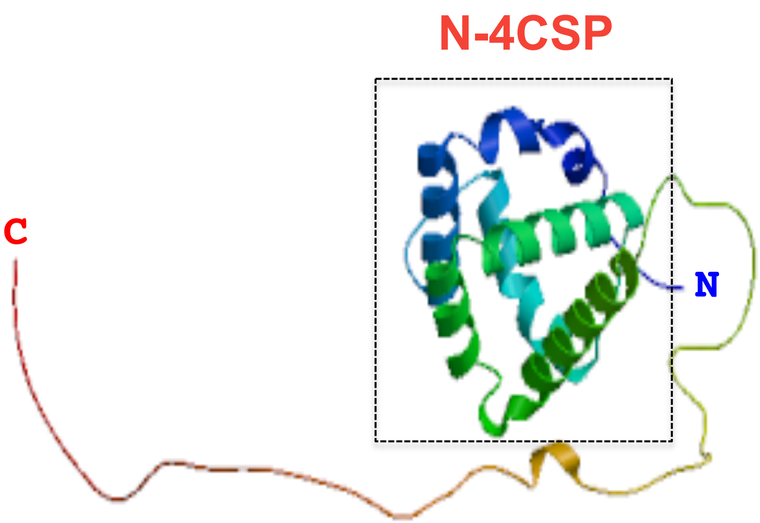

GMQE 0.37
Seq Id 71.34%

1kx9.1.A

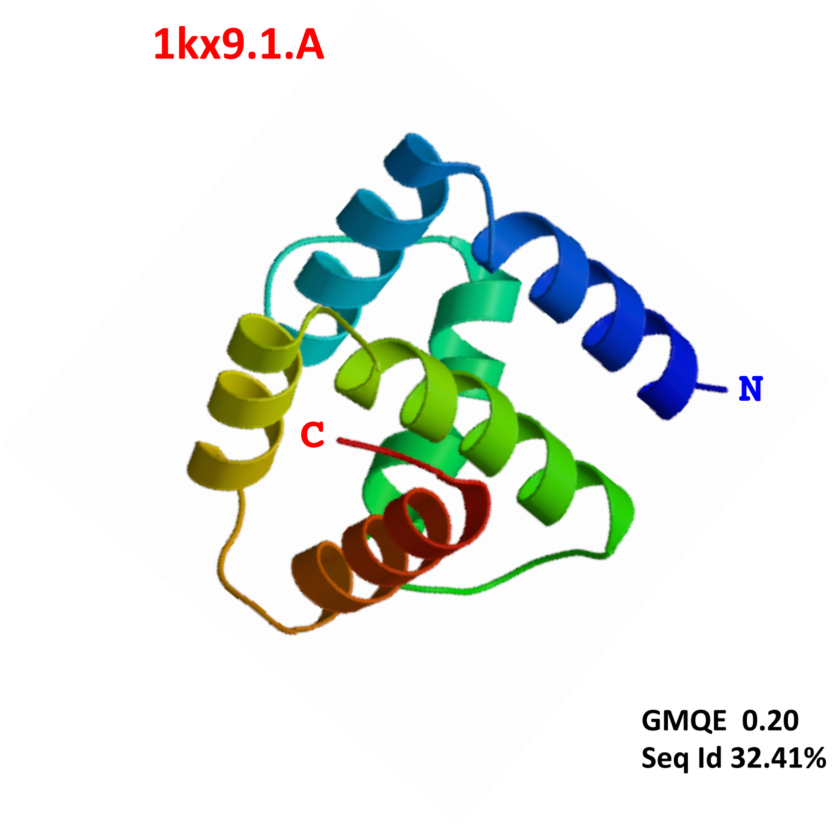

GMQE 0.20
Seq Id 32.41%

XM_055738352
Uranotaenia lowii
integumentary mucin C.1-like
Translation Initiation Factor (TIF)

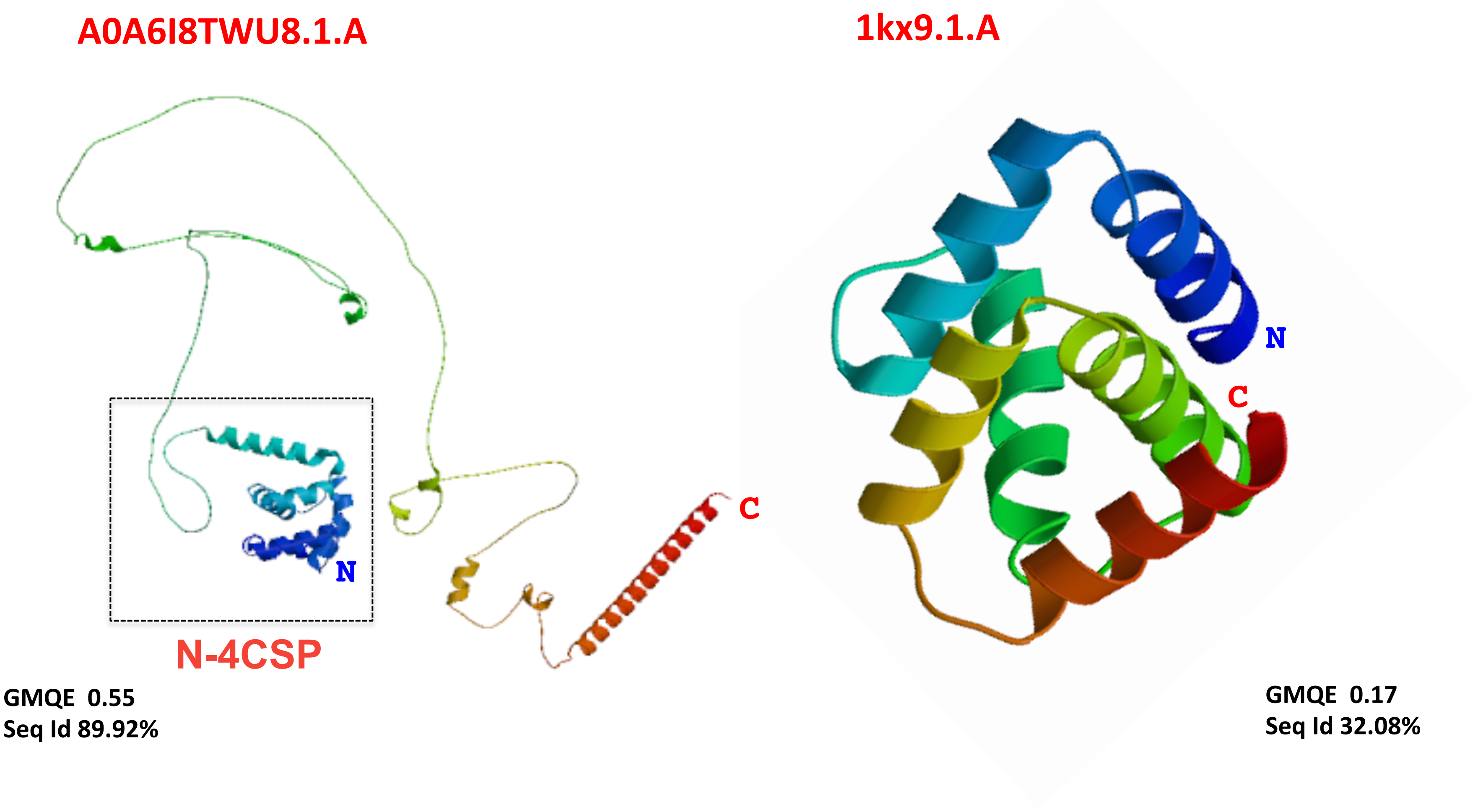

XM_029870982

Aedes albopictus

nuclear pore complex protein

(NPCP)

A0A6H0D4B0.1.A

GMQE 0.55
Seq Id 81.02%

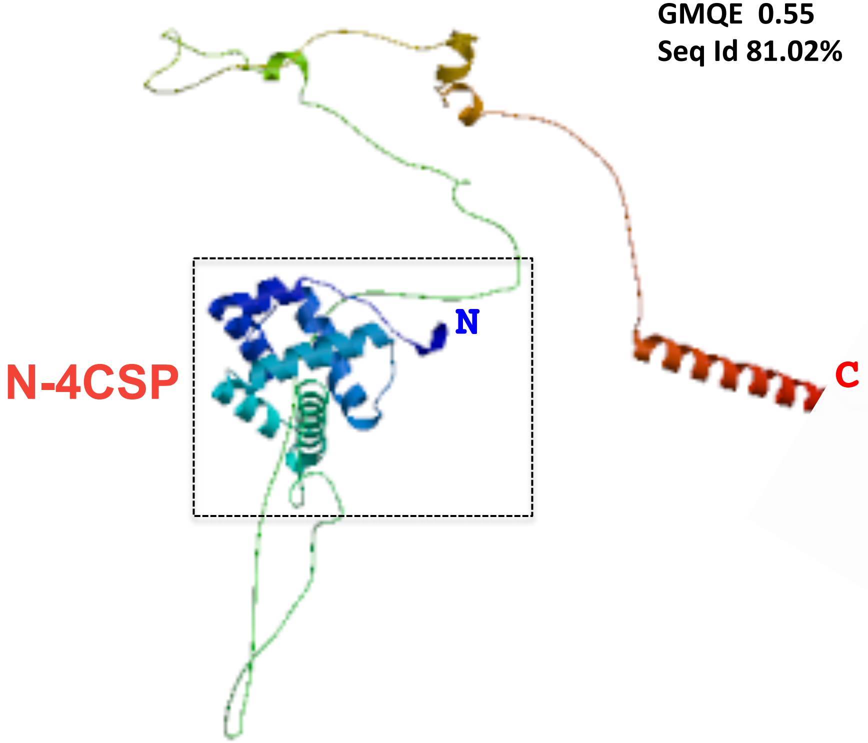

1kx9.1.A

GMQE 0.20
Seq Id 33.02%

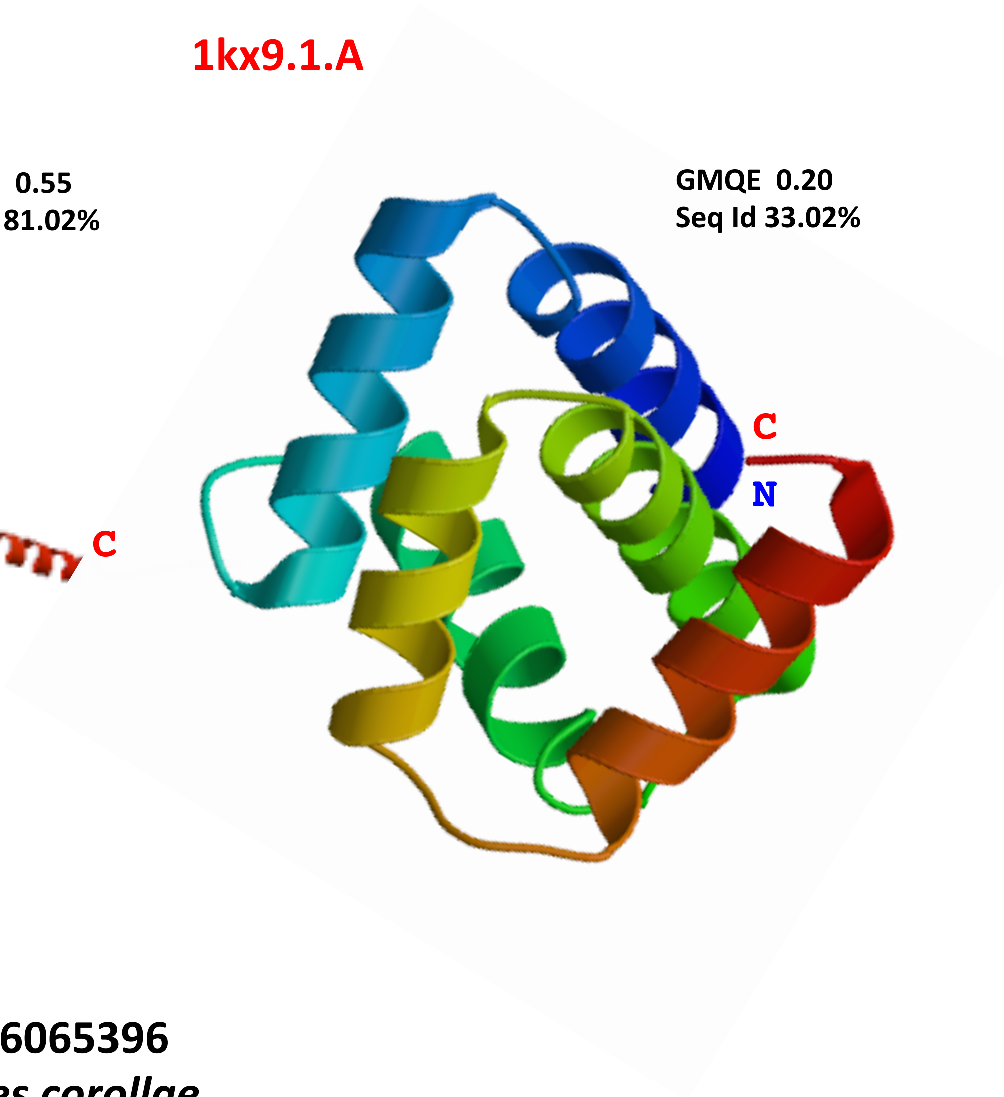

XM_056065396

Eupeodes corollae

rho GTPase-activating protein 17-like

(Rho)

A0A182NU82.1.A

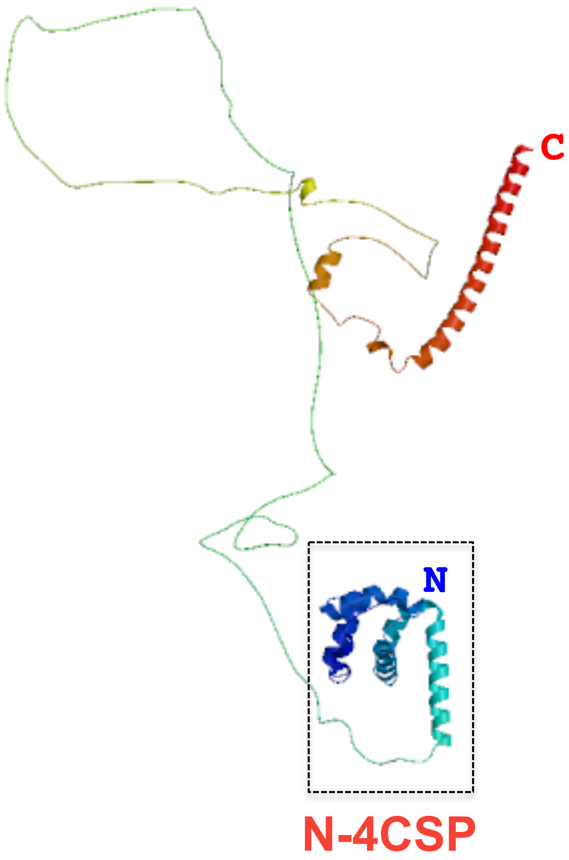

GMQE 0.53
Seq Id 84.12%

1kx9.1.A

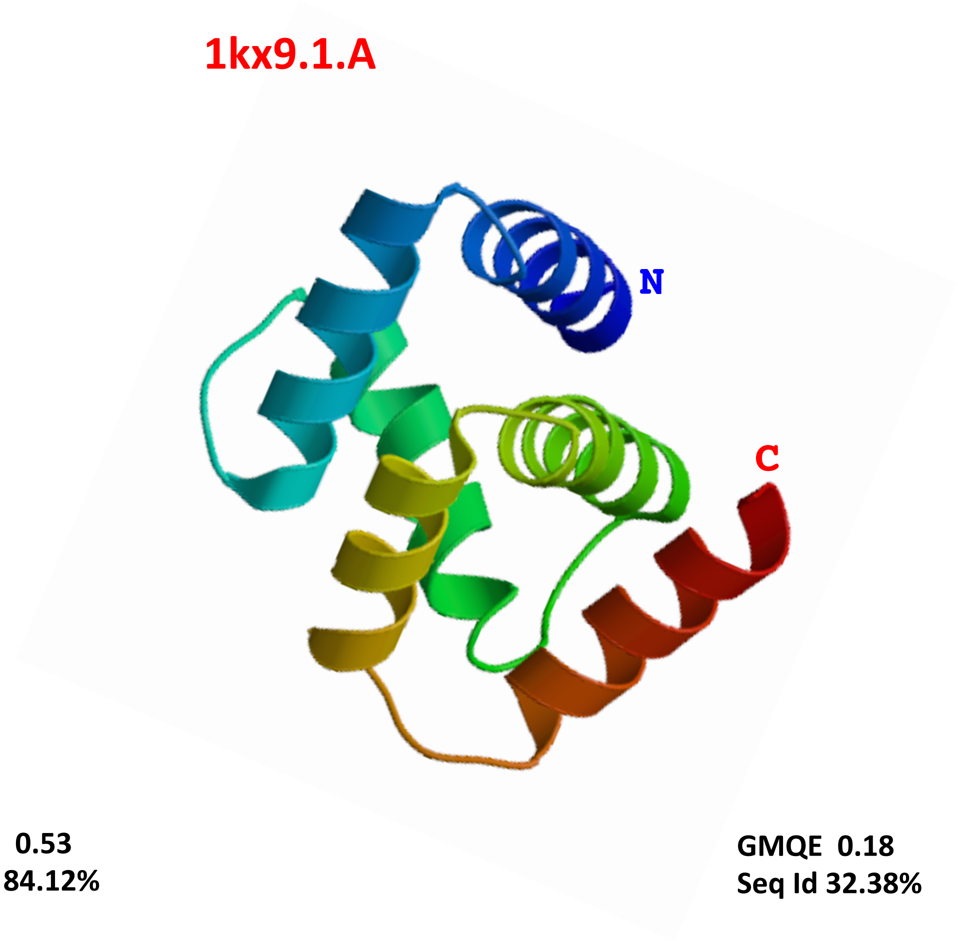

GMQE 0.18
Seq Id 32.38%

XM_050245628
Anopheles aquasalis
WAS/WASL-interacting protein family member 1-like isoform X2
(Mucin)

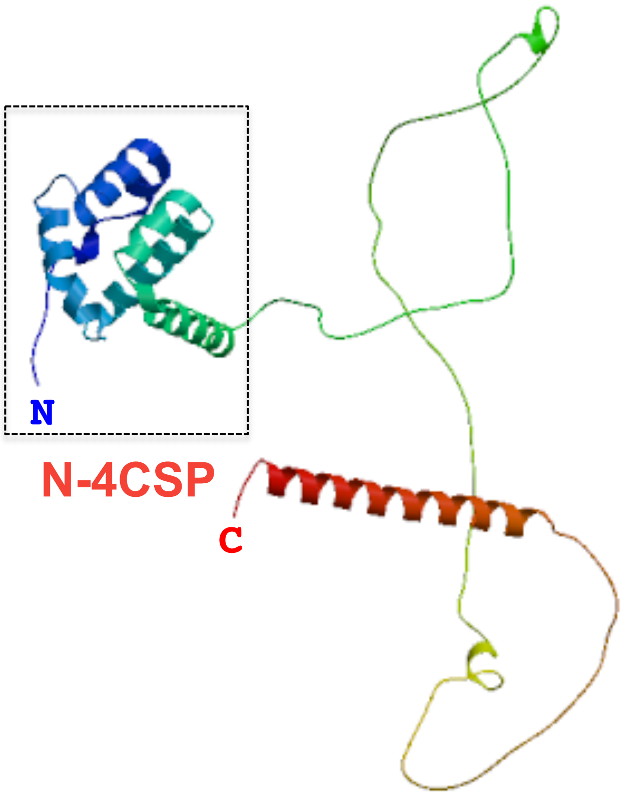

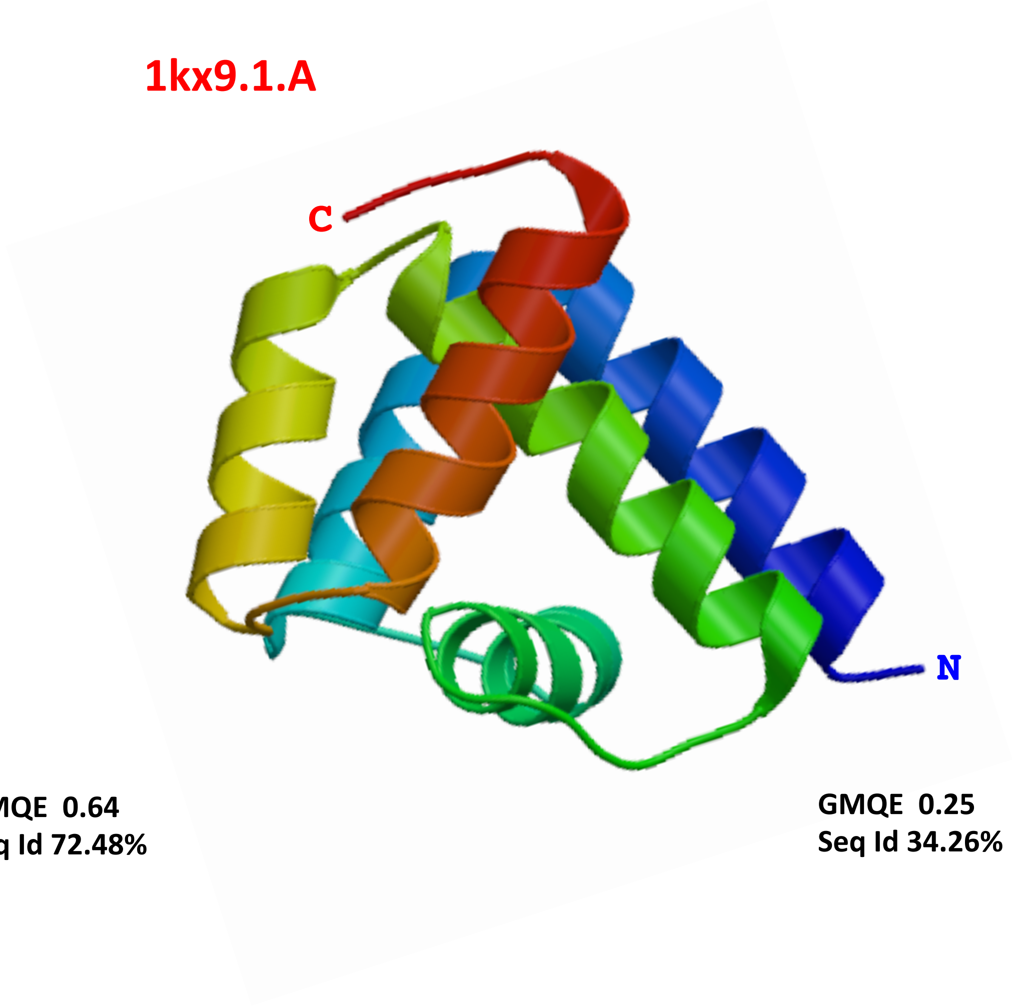

XM_047175293

Helicoverpa zea

actin cytoskeleton-regulatory complex protein PAN1-like (ASRP)

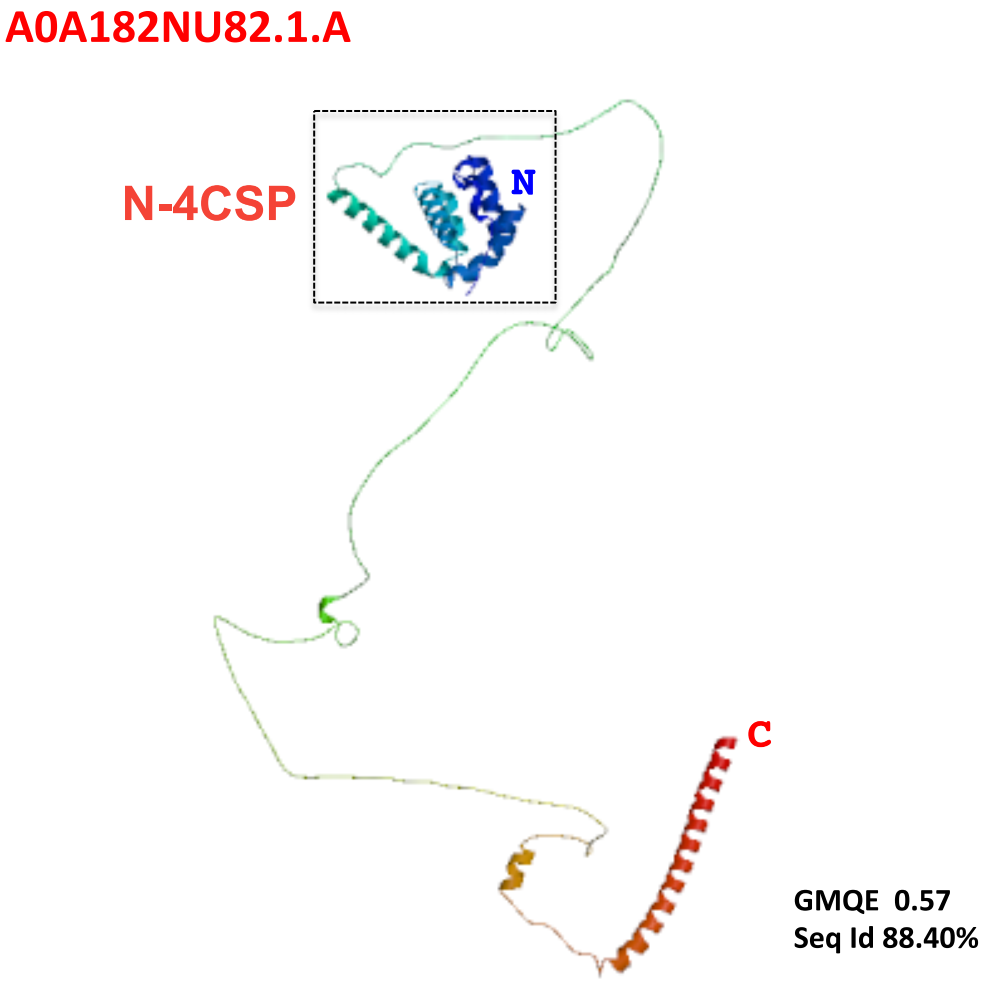

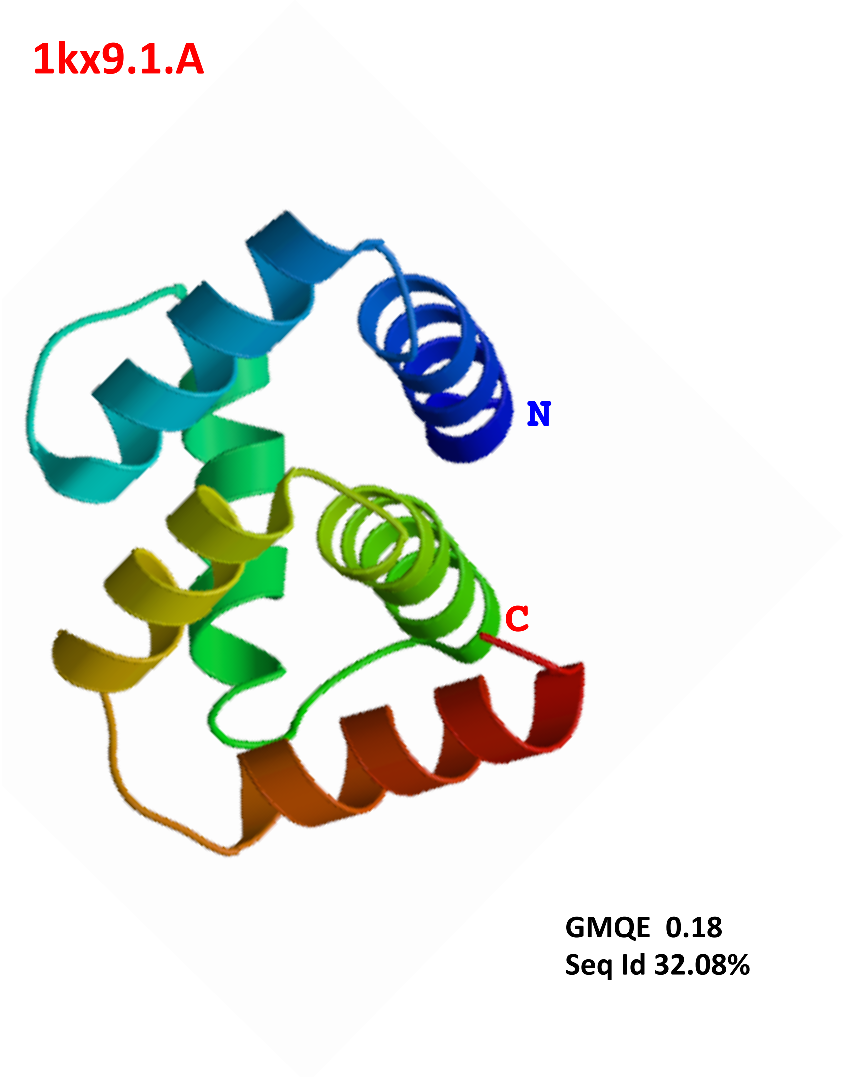

XM_058204117

Anopheles bellator

mucin-2-like

Protein UL36

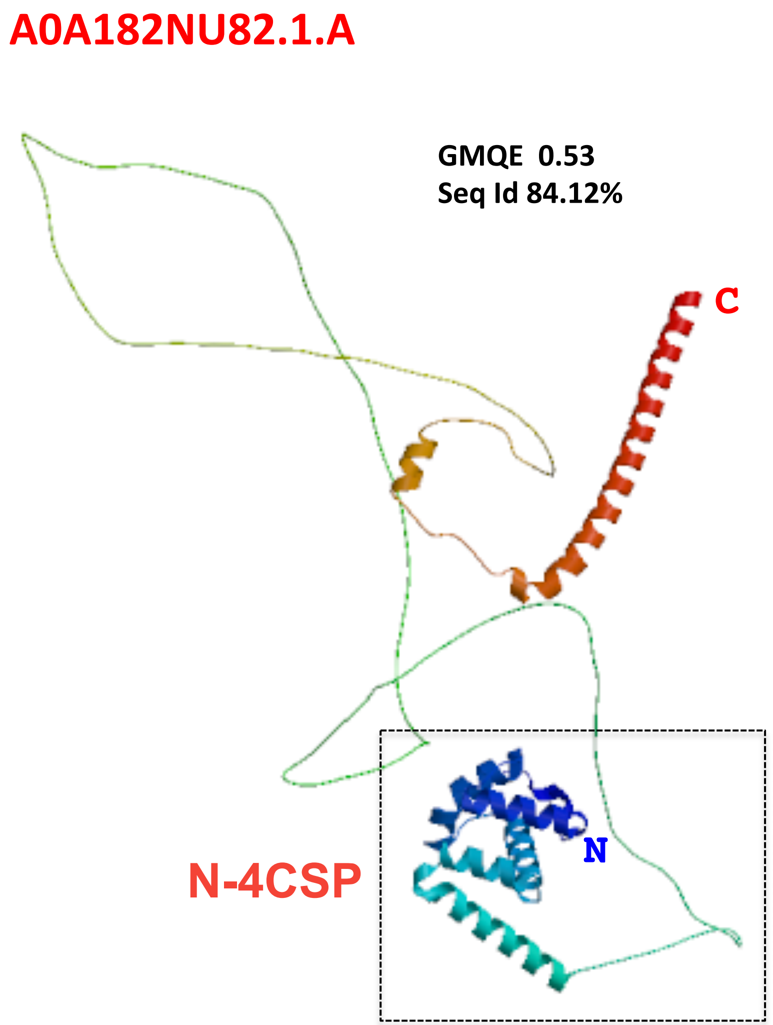

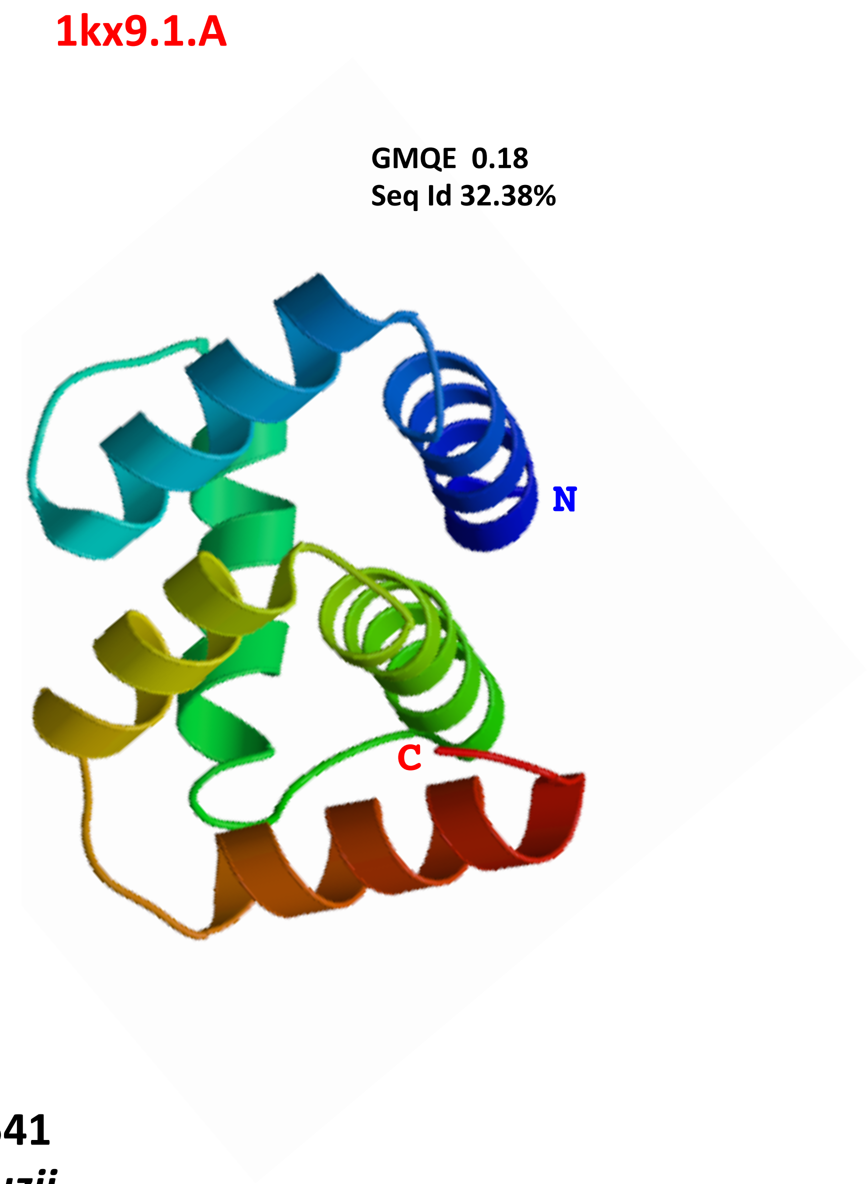

XM_053009541

Anopheles cruzii

WAS/WASL protein

Actin skeleton-regulatory complex (ASRC)

A0A1Q3FP99.1.A

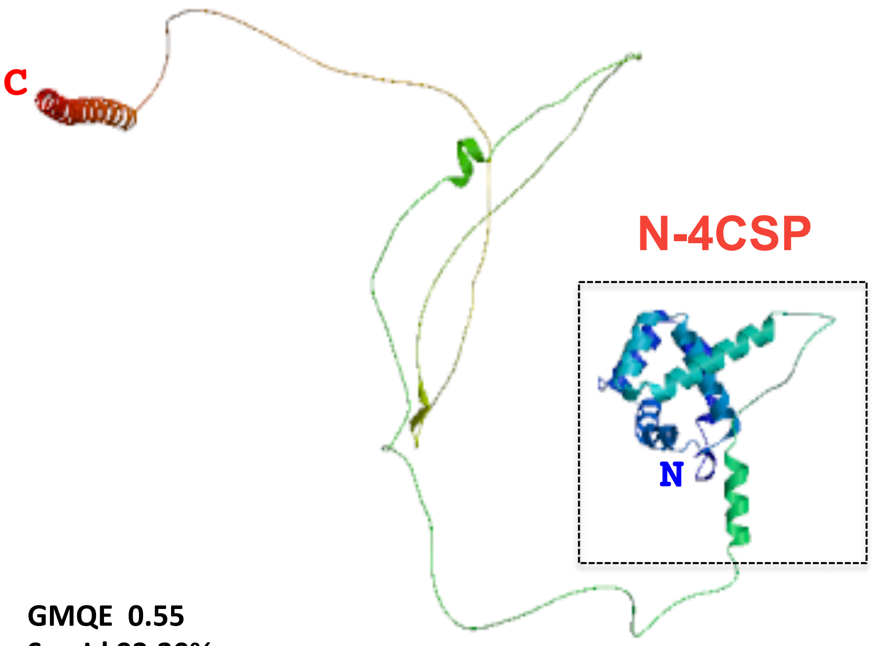

GMQE 0.55
Seq Id 93.30%

1kx9.1.A

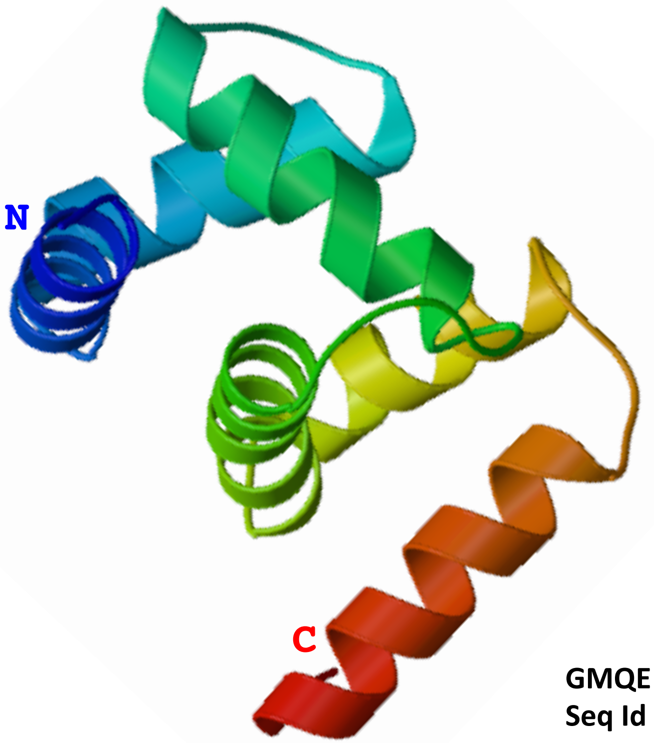

GMQE 0.17
Seq Id 29.91%

XM_038253956
Culex quinquefasciatus
cell wall protein DAN4
(CWP)

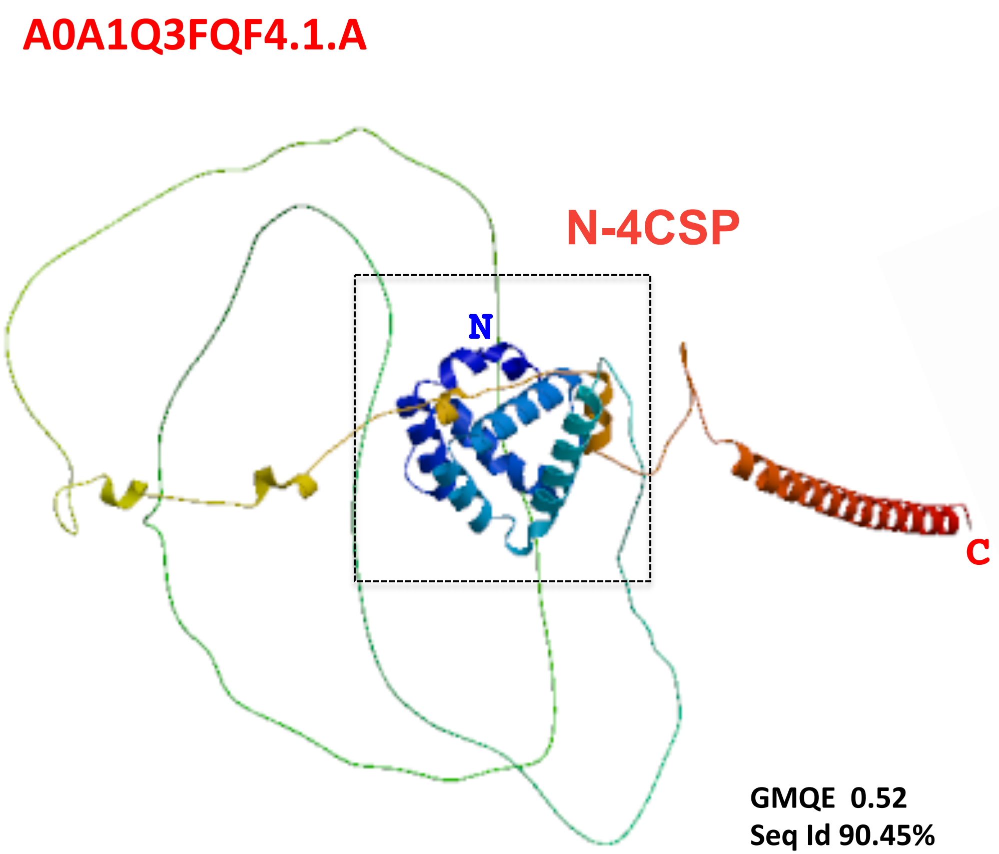

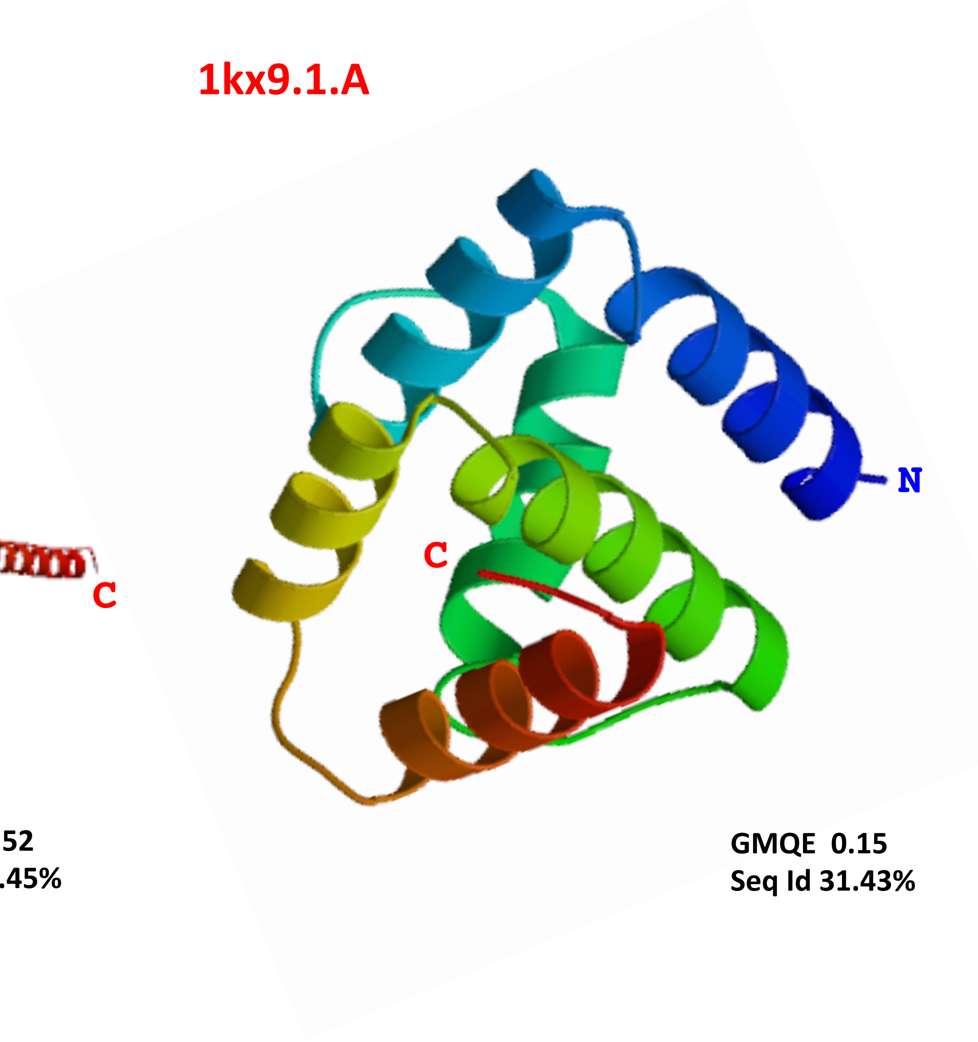

XM_052707633

Culex pipiens pallens

mucin-2-like

(Mucin)

A0A1V1WC08.1.A

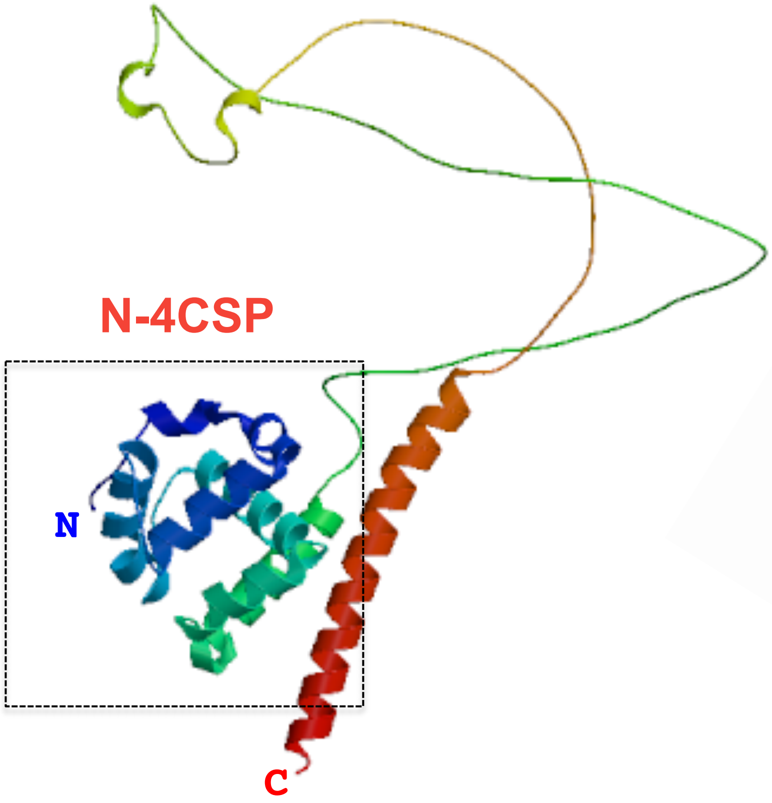

GMQE 0.64
Seq Id 70.31%

1kx9.1.A

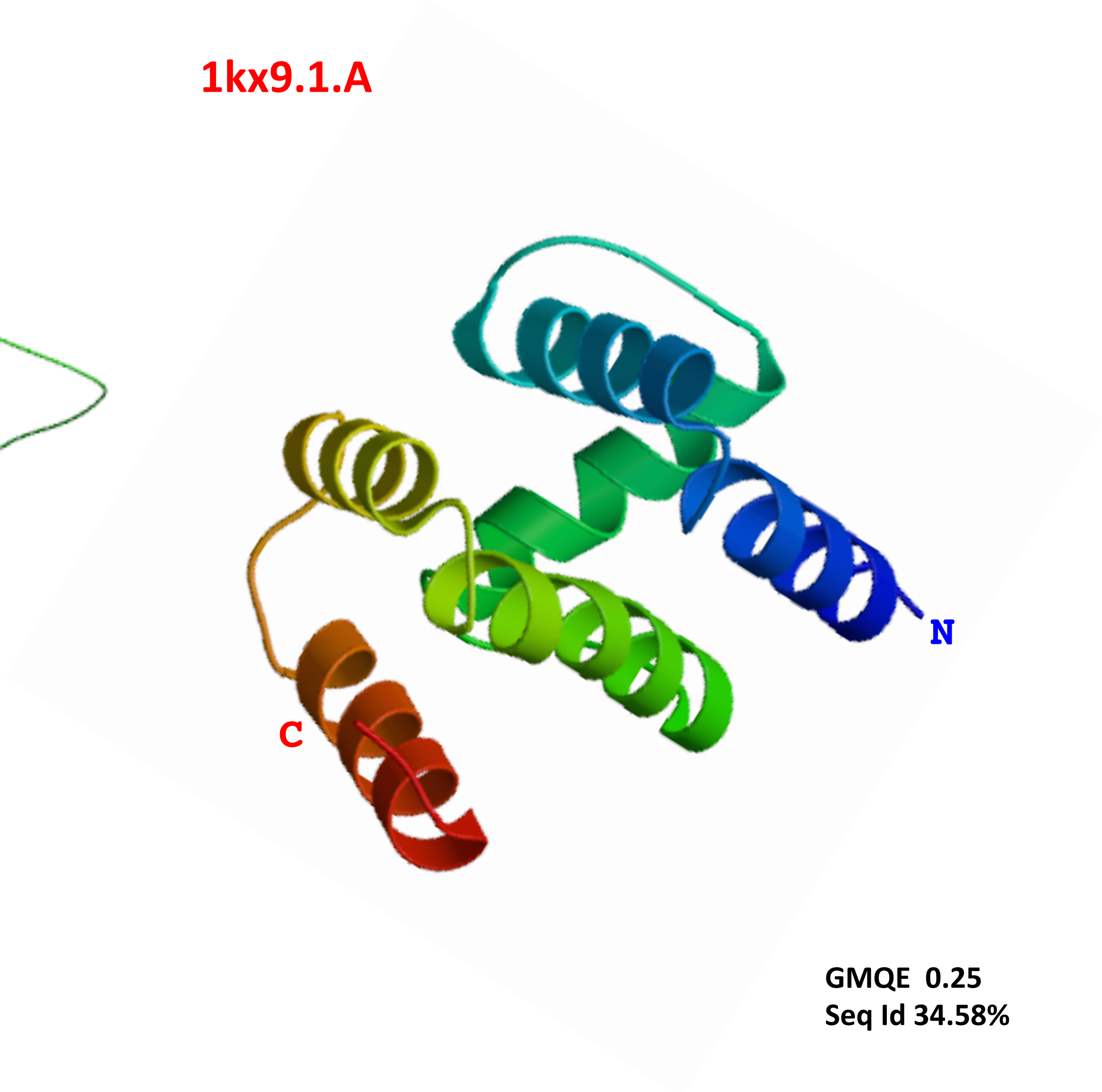

GMQE 0.25
Seq Id 34.58%

CAH2235359

Pararge aegeria aegeria

Herpes virus major outer envelope glycoprotein

(jg5928)

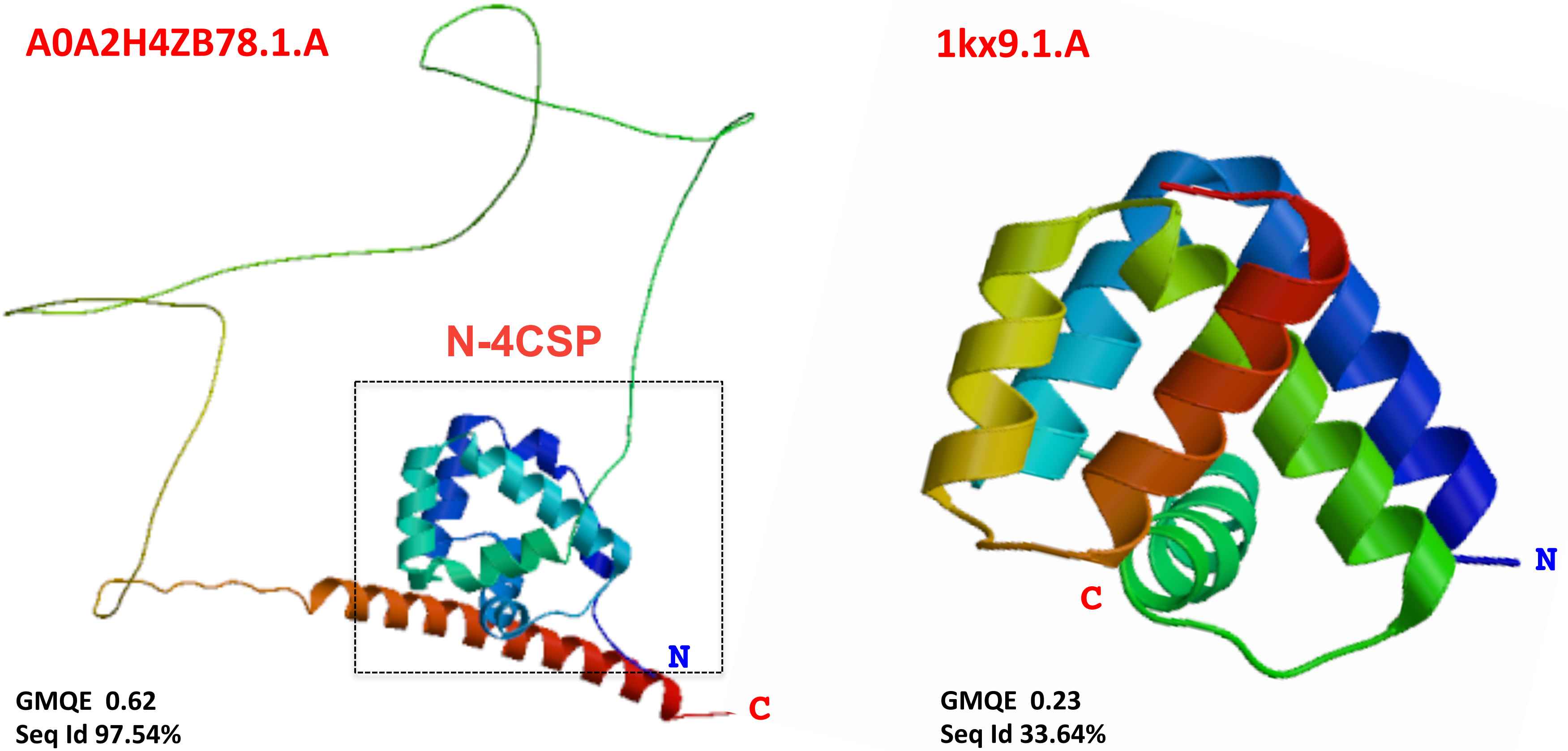

XM_018707509

Anoplophora glabripennis

cell wall integrity and stress response component 3-like (CWP)

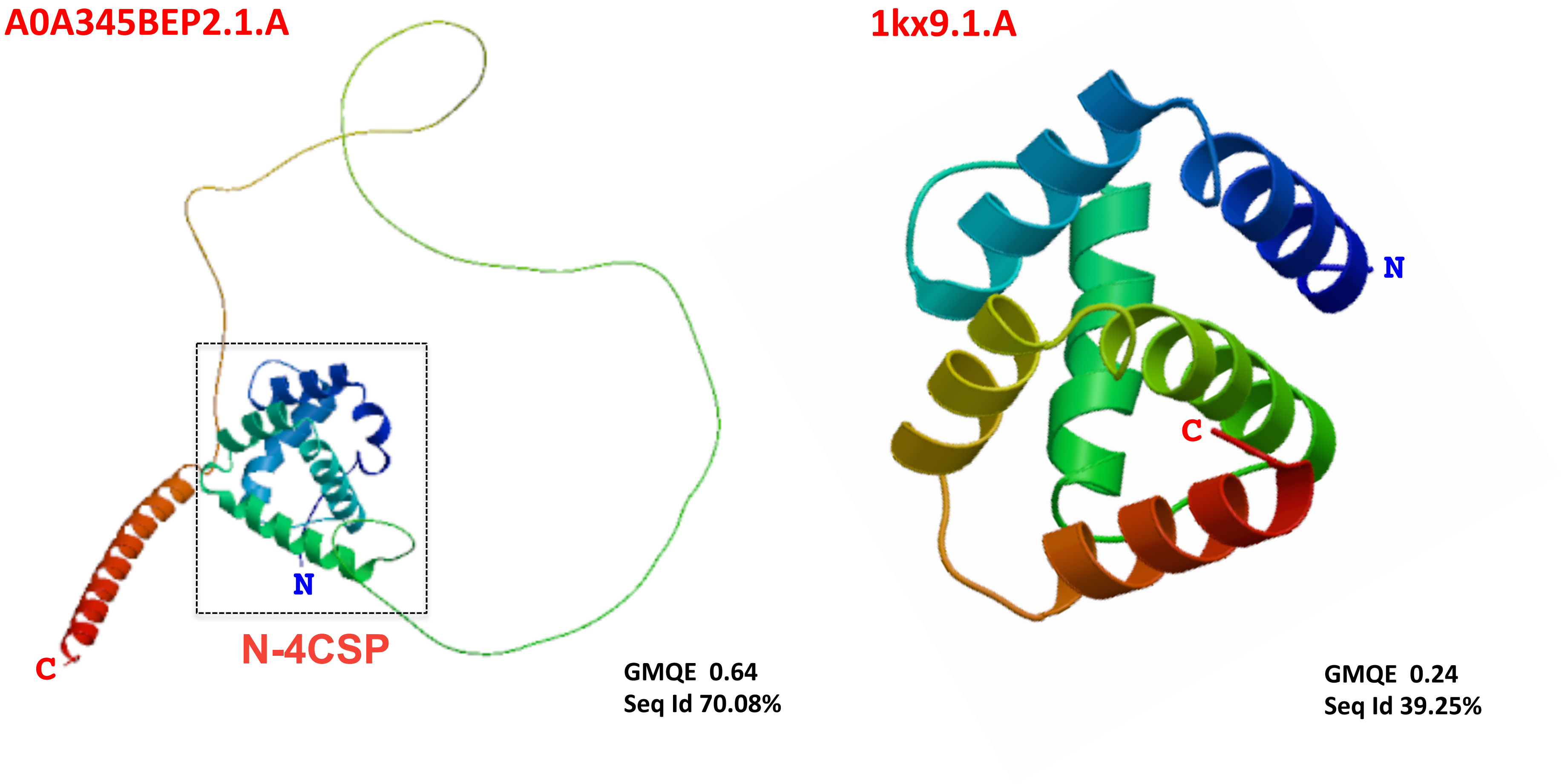

XM_048140790

Leguminivora glycinivorella

Complex-activating protein rickA-like isoform X2

mucin-2-like

A0A1Q3FQF4.1.A

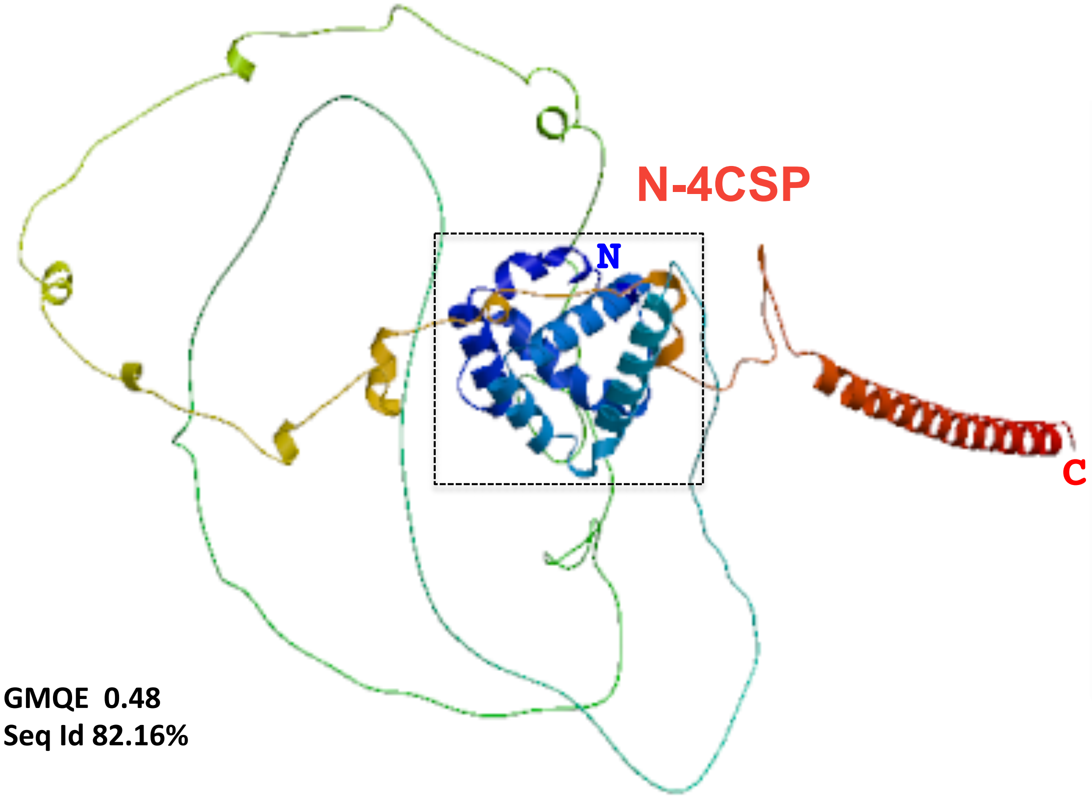

GMQE 0.48
Seq Id 82.16%

1kx9.1.A

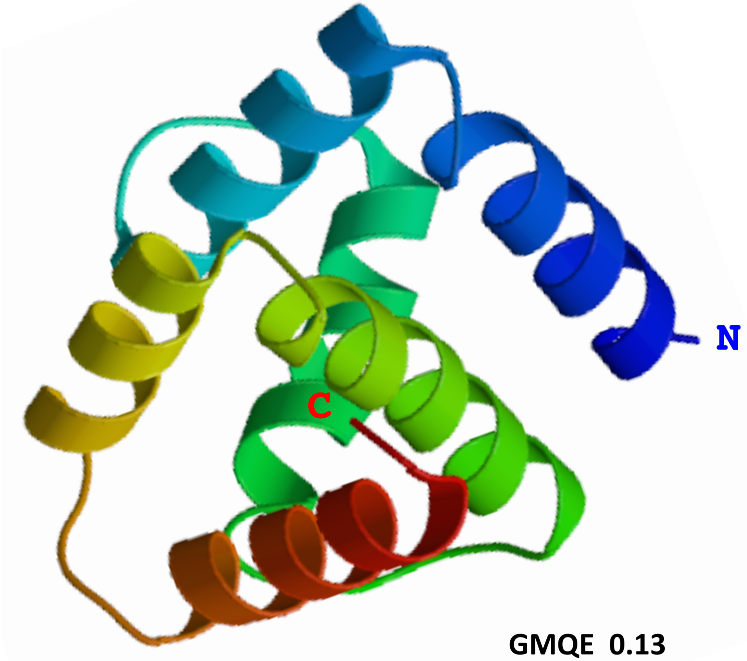

GMQE 0.13
Seq Id 30.19%

XM_038253951
Culex quinquefasciatus
cell wall protein DAN4
(CWP)

D6WUE8.1.A

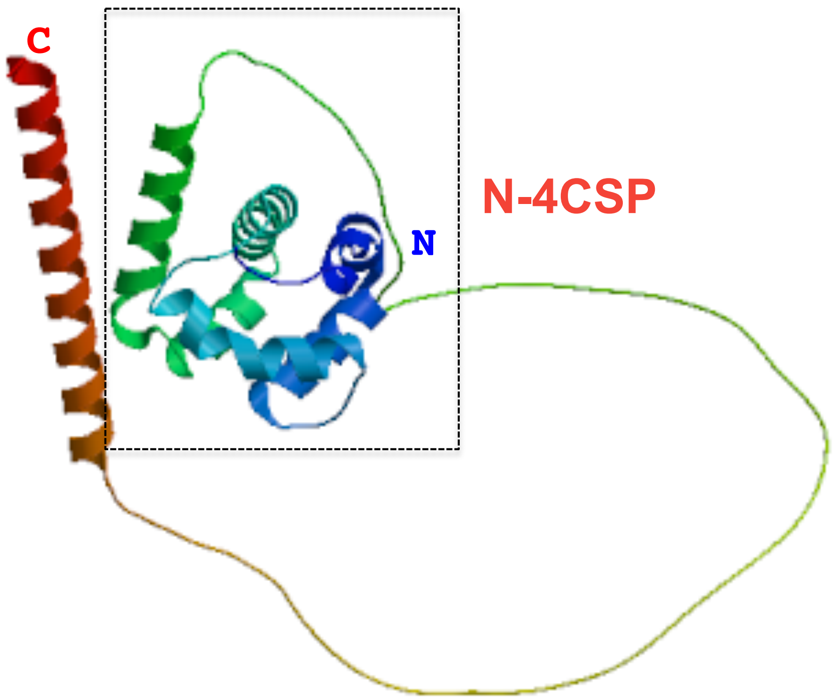

GMQE 0.29
Seq Id 33.64%

1kx9.1.A

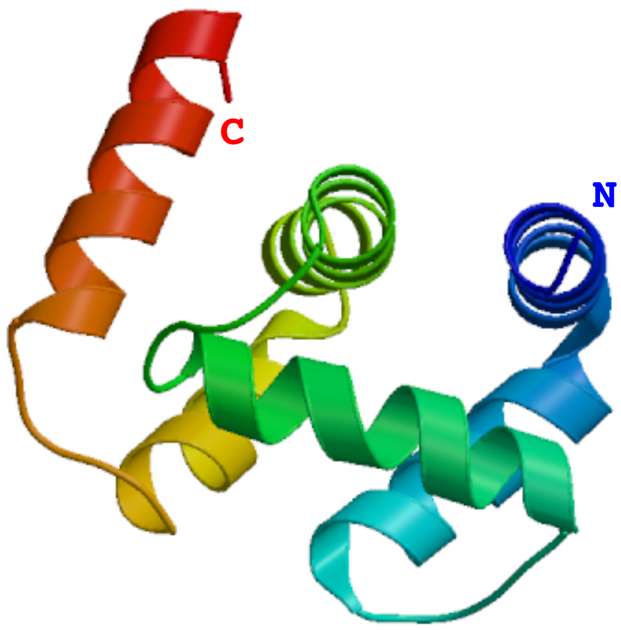

GMQE 0.70
Seq Id 88.79%

XM_044408485
Tribolium madens
Allergen Tha p 1-like
(IgE-binding protein)

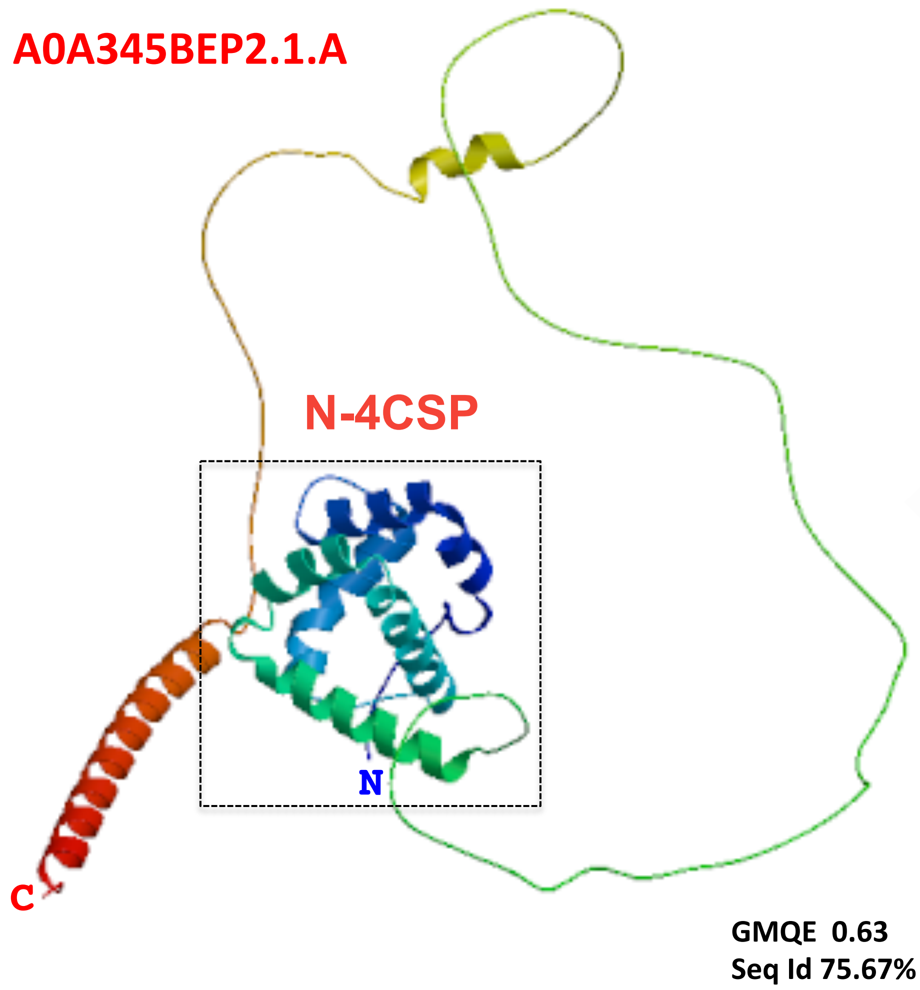

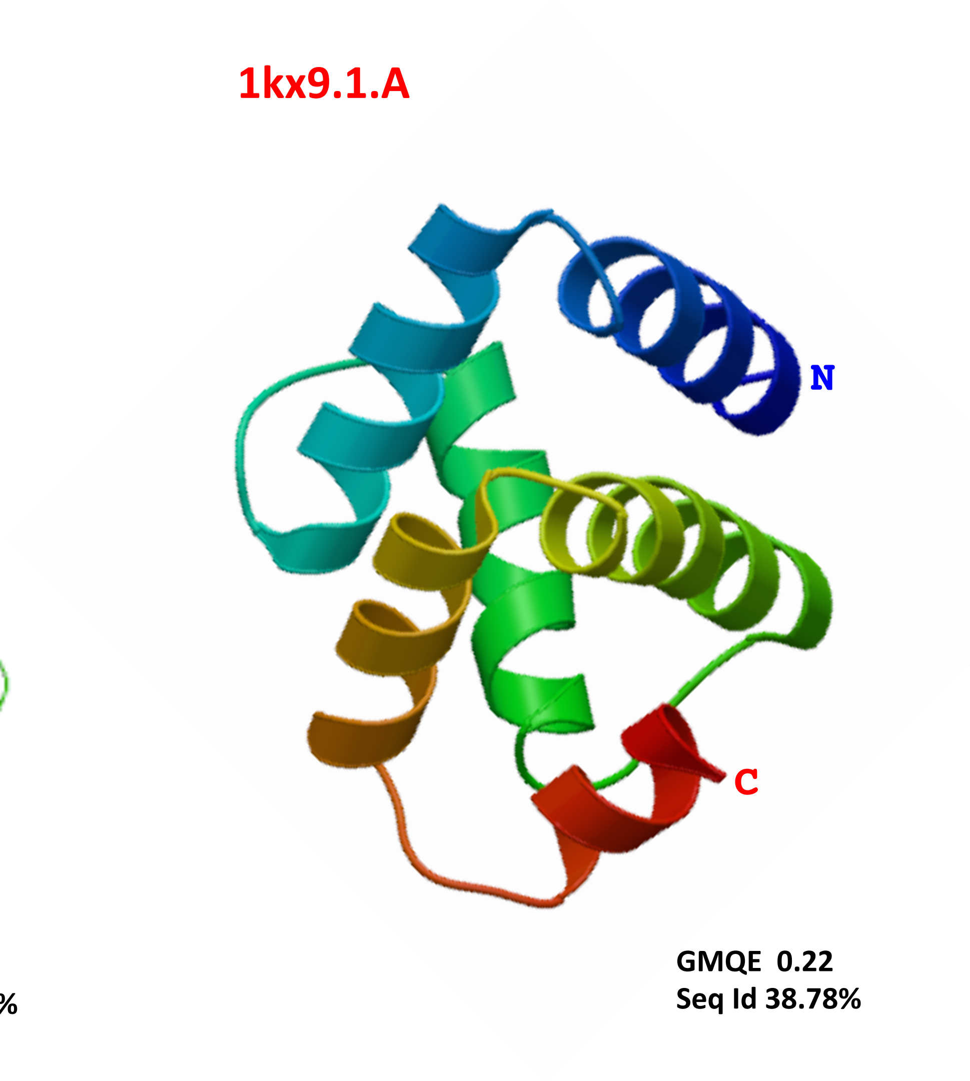

XM_053758324

Plodia interpunctella

Actin microtubule association protein

(Formin-1-like)

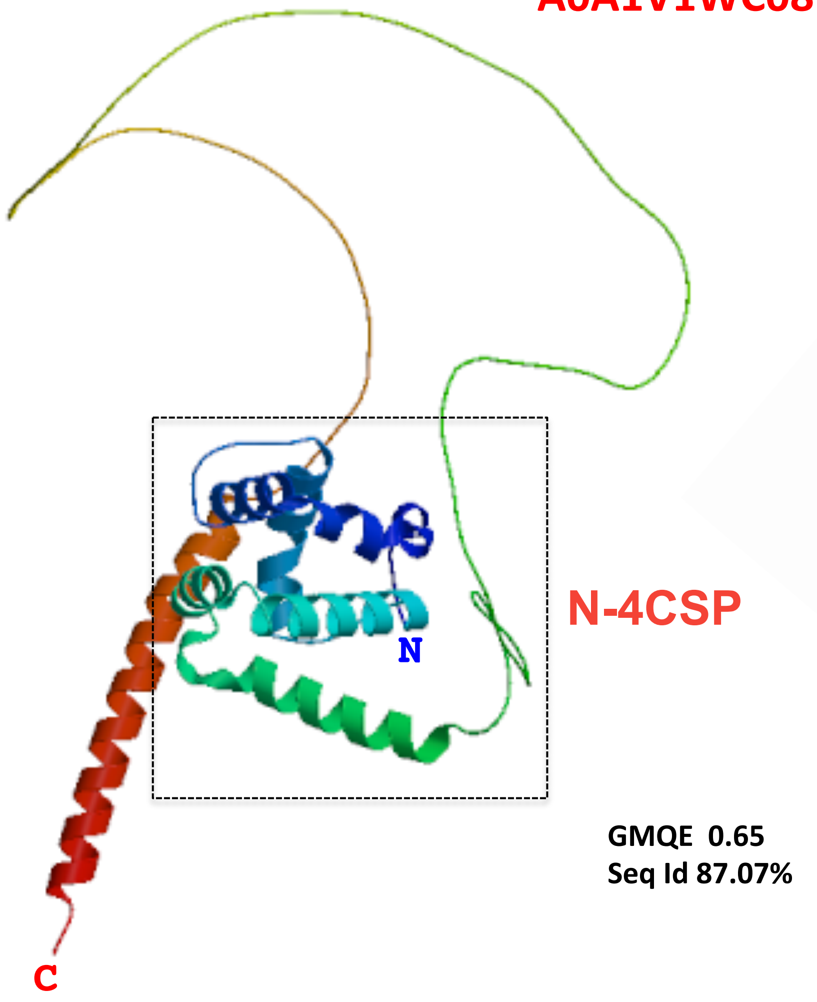

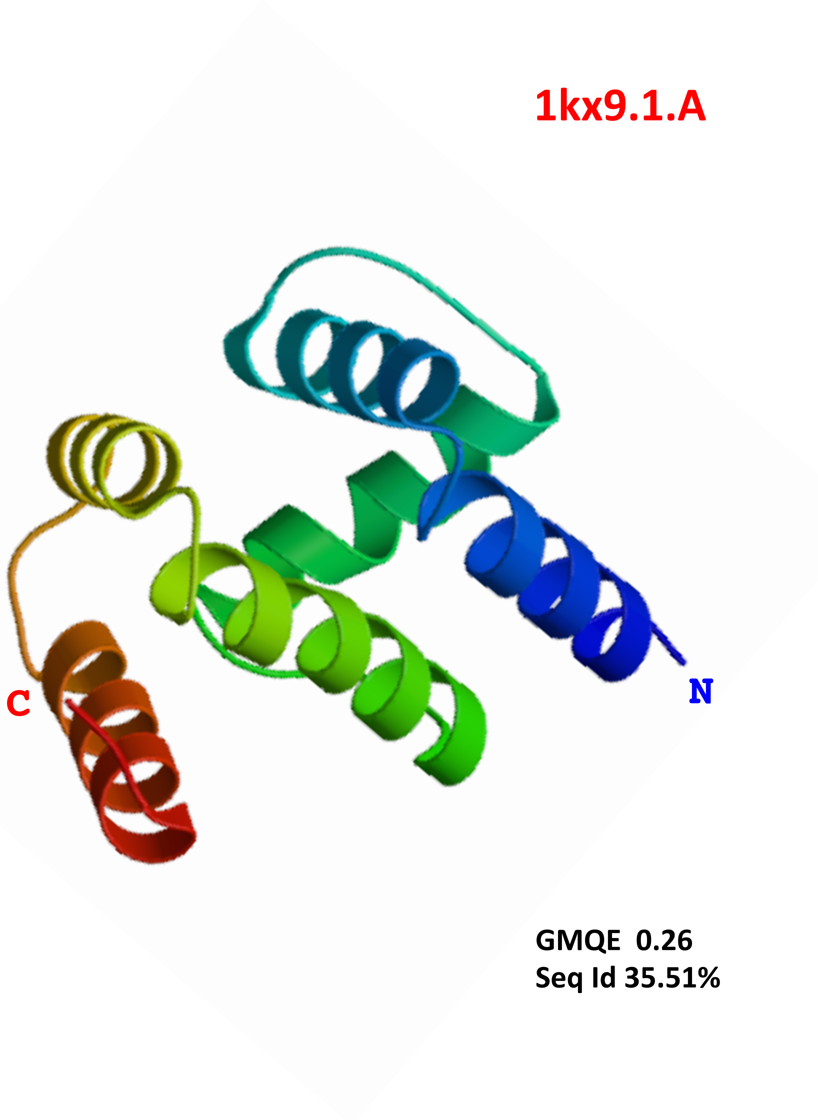

XM_022960083

Spodoptera litura

actin cytoskeleton-regulatory complex protein pan1-like

(AS RP)

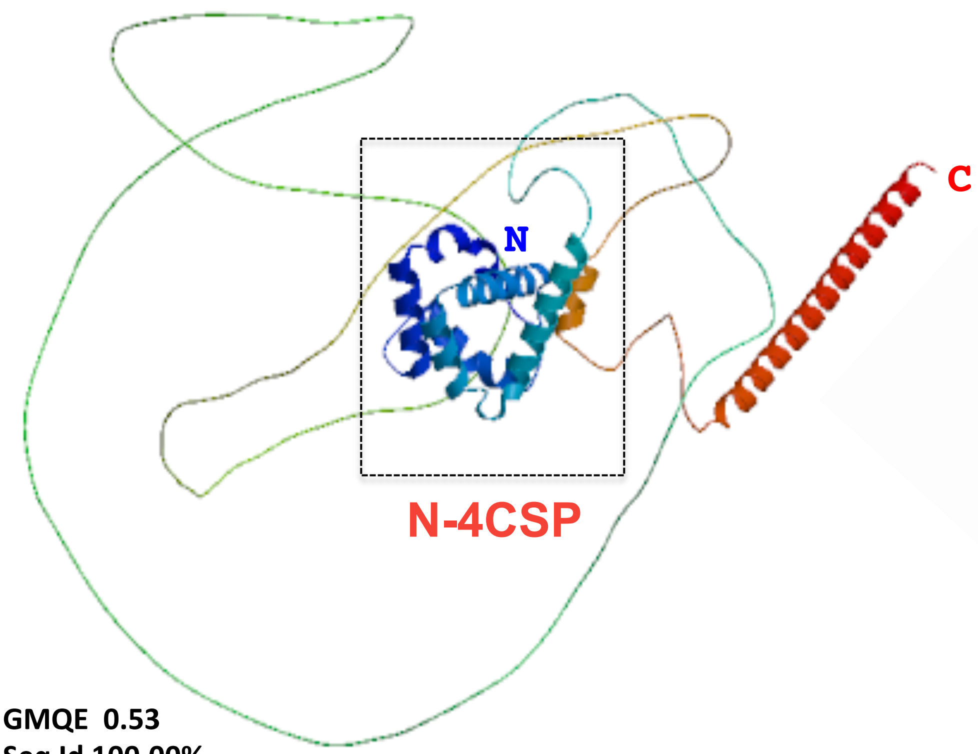

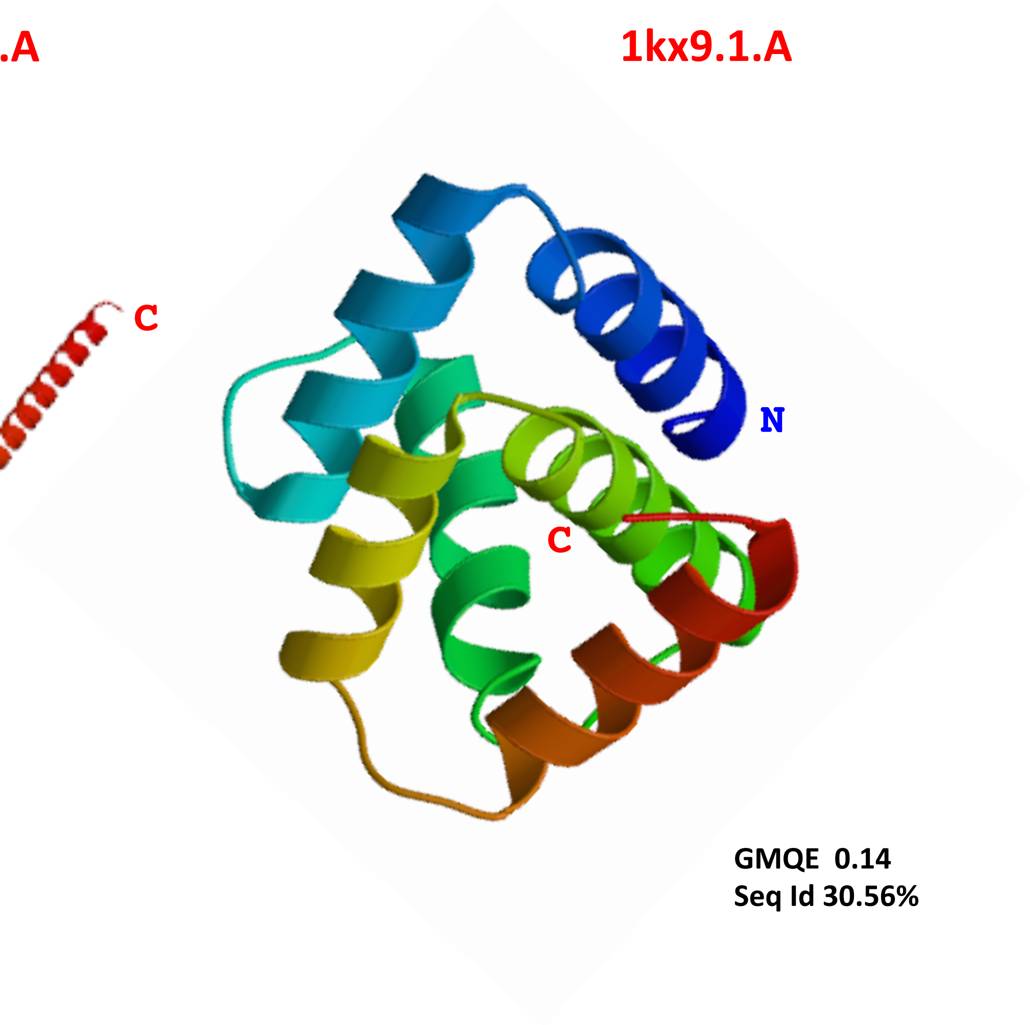

XM_021847563

Aedes aegypti

nuclear pore complex protein

(NPCP)

A0A8I8TWU8.1.A

GMQE 0.55
Seq Id 89.92%

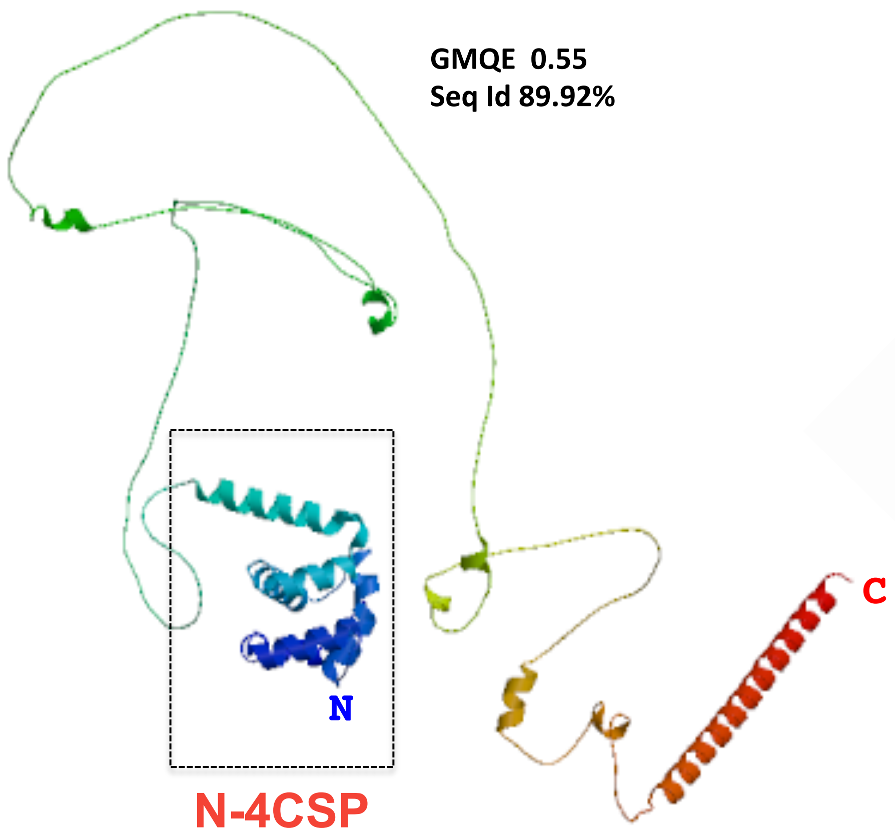

1kx9.1.A

GMQE 0.17
Seq Id 32.08%

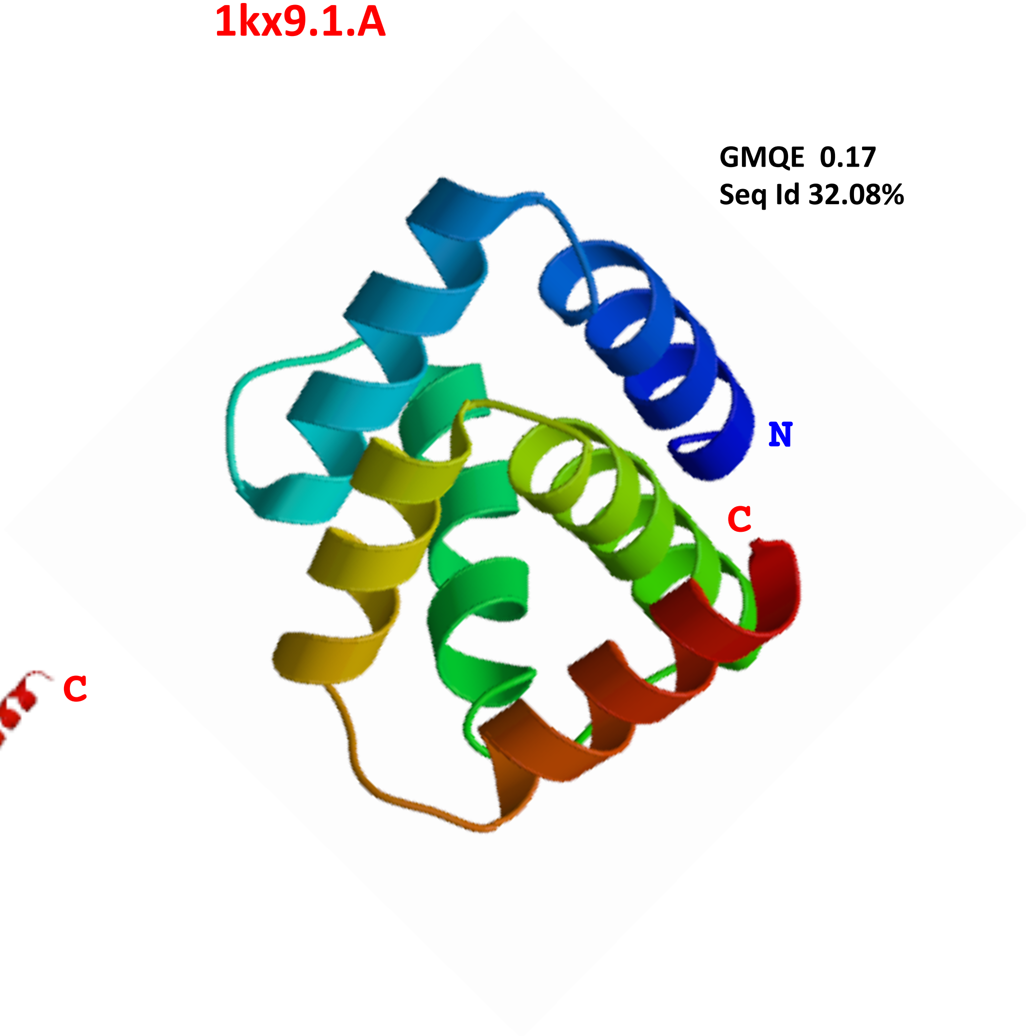

XM_029870982
Aedes albopictus
SamkC nuclear pore complex protein
(NPCP)

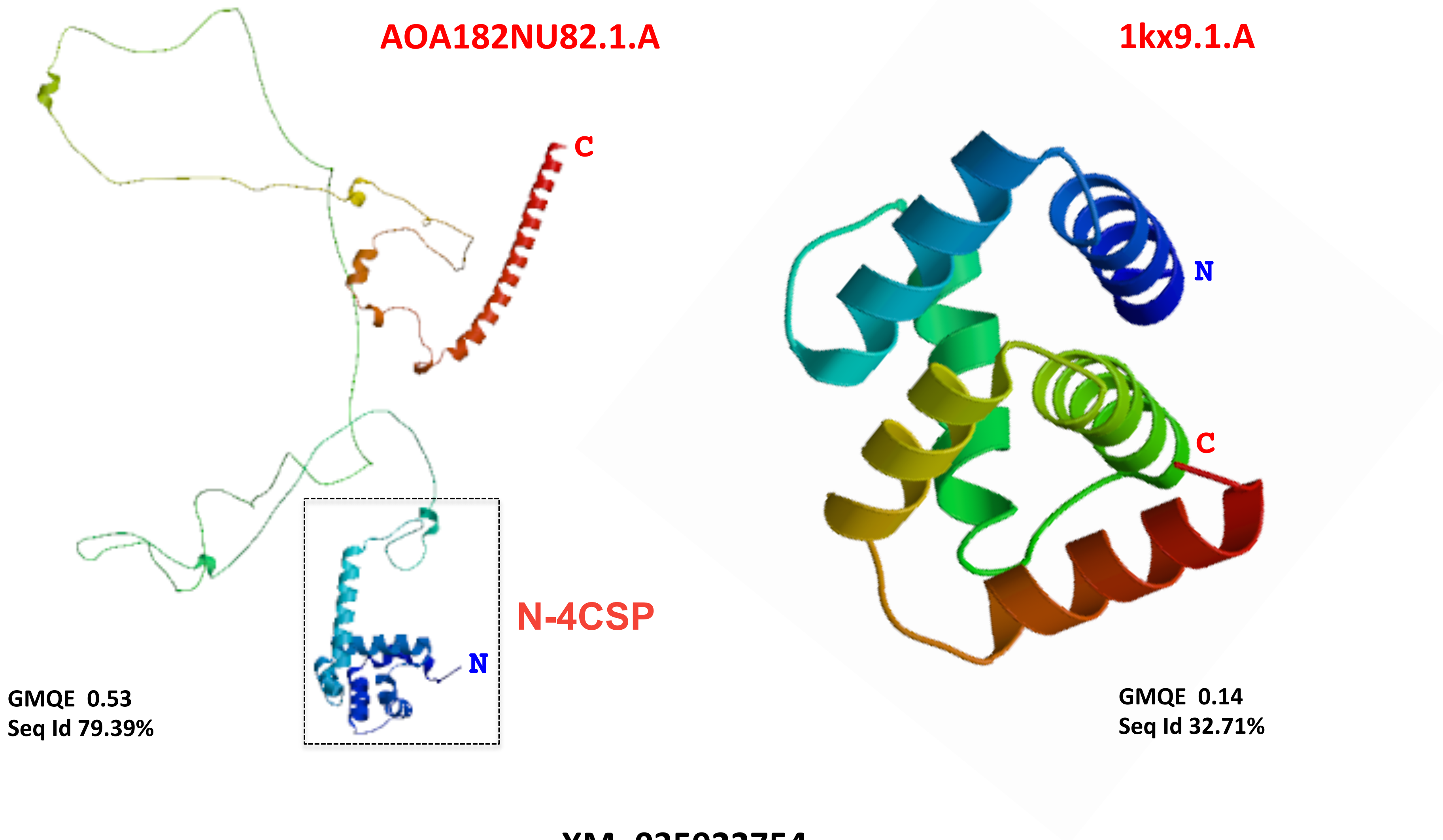

XM_035922754

Anopheles albimanus

extensin-like Leucine-Rich repeat (LRR)

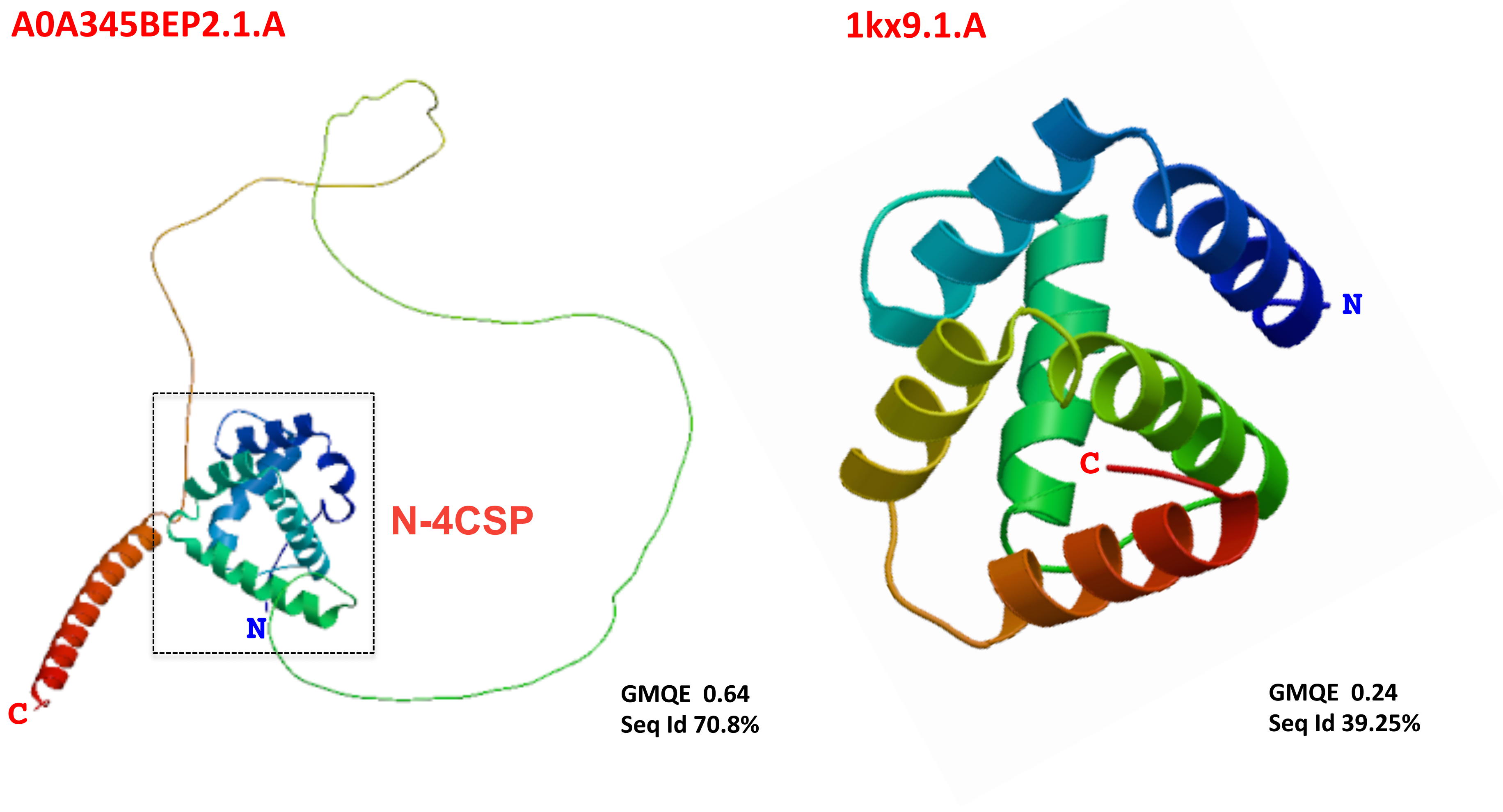

XM_050021059
Pectinophora gossypiella
YLP motif-containing protein 1-like
Nuclear nucleoside kinase (NNK)
