# Supplementary material for: Renaming the ‘OS-D/CSP’ Family (Part 2): ‘4-Cysteine Soluble Proteins’ (4CSPs)—Intracellular Functions
Source: Insects. 2026 Sep 8;17(9):940. doi: 10.3390/insects17090940 (PMC13607926; doi:10.3390/insects17090940)
Supplement: Supplementary file 1 [file insects-17-00940-s001.zip › TableS1.pdf]

Organism	Order	Genome Assembly	Transcript Annotation	Length (bps)	N-terminus	C-terminus	Identity % (Order subject)
Acyrthosiphon pisum *Acypi*	Hemiptera	EQ110797	ACYPI000094	1236	TSSYPTRY	KLTNNTTK	92 (Hemiptera)
		EQ110797	ACYPI009116	363	PAKYTTKY	AKKLNIDV	90 (Hemiptera)
		EQ116326	ACYPI002311	1039	LKKFLSTL	FLENLNTD	78 (Hemiptera)
		EQ117790	ACYPI000097	815	GTAYTTKY	KKKGVVKF	97 (Hemiptera)
		EQ121783	ACYPI000093	812	FPAYMKRF	KLNKFLDA	90 (Hemiptera)
		EQ121783	ACYPI000345	1554	SGYYLSTY	IINFMNQK	80 (Hemiptera)
		EQ122410	ACYPI005842	844	KDSSLPNV	LKQYQSGF	96 (Hemiptera)
		EQ125317	ACYPI000096	669	FTLAEEKY	ERAAAAKH	95 (Hemiptera)
		EQ125317	ACYPI003368	2009	EDDDKPDF	TANIVKIQ	79 (Hemiptera)
		EQ126525	ACYPI000095	645	QEKYSTKY	KKLEKFSA	91 (Hemiptera)
Bemisia tabaci *Bemta*	Hemiptera	LOC109029910	Bta06193+	940	AEFYTSQF	EKALLGSR	47 (Diptera)
		LOC109030432	Bta07590	596	EQSDLEKF	DLEKFMSS	42 (Lepidoptera)
		LOC109030655	Bta07752+	456	MKEALKRM	EKFFASSK	46 (Hemiptera)
		LOC109030749	Bta08123	384	AEFYTSQF	EKALLGSR	51 (Ephemeroptera)
		LOC109030750	Bta05122	384	AETYTTEF	EEAFLGSL	48 (Hemiptera)
		LOC109031385	Bta08846	689	DDFYSDKY	AKFKARFP	59 (Hemiptera)
		LOC109031577	Bta09312	1140	AEFYTTSQ	EKALLGAR	48 (Hymenoptera)
		LOC109031699	Bta09832	330	SAHLGPNF	KIAAKYKK	58 (Hymenoptera)
		LOC109032962	Bta06193+	594	AEFYTSQF	EKALLGSR	53 (Hymenoptera)
		LOC109034312	BtabCSP1	381	ADTYTTQF	KALFGQTL	54 (Diptera)
		LOC109034660	Bta13934	497	AEFYTSQF	EKALLASP	50 (Diptera)
		LOC109040632	Bta10843	569	WGTSGQRV	AKYGRAFF	51 (Hemiptera)
		LOC109040648	XM_019026637	492	DEKLLSKY	ESAKAPKA	64 (Coleoptera)
		LOC109040996	Bta03345	580	RAQENQKQ	AEKDAPKK	43 (Diptera)
		LOC109041650	Bta07752+	907	MKEALKRM	EKFFASSK	46 (Hemiptera)
		LOC109042107	Bta03344	2151	RATYTTKY	PKRGQETG	60 (Hemiptera)
		LOC109042122	XM_019058691	741	EEKYSDKY	LKKETGQA	47 (Dictyotera)
		LOC109042131	BtabCSP2	396	AAPAEDKY	QEYLKASA	53 (Hemiptera)
		LOC109043307	BtabCSP3	417	ESTYTNKY	AEKLGIKV	53 (Hymenoptera)
		LOC109043693	Bta03347	631	TLCSSPAL	EFMDEDND	†40 (Coleoptera)

		LOC109043753	Bta09642	1973	PQTTRATI	KIVKQFTS	75 (Hemiptera)
			KY305451	370	ASTYTTKY	YADEAKKR	94 (Hemiptera)&
		LOC109045321	Bta12075	384	AEFYTSQF	EKALLGSR	47 (Coleoptera)
Halyomorpha halys *Halha*	Hemiptera	LOC106678159	XM_014416524	381	AEVYTSKY	TTAAPAAA	92 (Heteroptera)
		Scaffold214	XM_014418950	387	ERQVLARL	RMEKFAAT	90 (Heteroptera)
		LOC106681195	XM_014421395	330	CAESRPSV	ILKQYAGQ	95 (Heteroptera)
		LOC106681317	XM_014421569	381	QQSYTDKW	KYEYEEDK	87 (Heteroptera)
		LOC106681351	XM_014421626	903	ATTYTTKY	EKRGIKIN	71 (Heteroptera)
		LOC106681352	XM_014421627	363	ADMYTTKY	RKRHNIKA	63 (Heteroptera)
		LOC106681353	XM_014421628	529	KEGYTTKY	YRKKHAQS	63 (Heteroptera)
		LOC106681354	XM_014421629	686	QSAKIDKY	QKLRKLYA	55 (Heteroptera)
		LOC106681357	XM_014421632	478	PDGYTTKY	AEKRGIKI	95 (Heteroptera)
		LOC106681358	XM_014421634	503	KGSYTTRY	AEKRGIKI	63 (Heteroptera)
		LOC106686897	XM_014430494	465	PDGYTTKY	AEKHGIKI	87 (Hemiptera)
		LOC106686898	XM_014430495	408	LAKPTTKT	AEKRGIKL	84 (Heteroptera)
		LOC106688012	XM_014432270	456	EAQLLGGT	RFTPQSSG	85 (Heteroptera)
		LOC106688752	XM_014433350	435	IDVYEKIL	IKEFLETY	85 (Heteroptera)
		LOC106691225	XM_014436935	381	AMSVPTRY	EKRGVKFS	61 Ser/Thr Kinase
		LOC106691226	XM_014436938	384	VTTYTTKY	NKGIKMVV	62 (Heteroptera)
		LOC106691228	XM_014436939	495	PDGYPTKY	AEKRGIKL	72 (Heteroptera)
Pachypsylla venusta Pacve	Hemiptera	Scaffold96	PVEN002856	357	LSKYDNVN	TYRAKYGV	54 (Psylloidea)
		Scaffold122	un	un	CWSYDSTY	LEAFLASP	54 (Psylloidea)º
		Scaffold301	PVEN005587	240	TNQESALL	--------	62 (Psylloidea)
		Scaffold301	PVEN005588	213	TTKYDNID	--------	72 (Psylloidea)
		Scaffold301	PVEN005589	315	--------	NSNLFLGT	78 (Psylloidea)
		Scaffold301	PVEN005590	381	TTKYDNVN	YQSQLANL	76 (Psylloidea)
		Scaffold301	PVEN005591	351	ISLIHCNK	LKAGTLKL	80 (Psylloidea)£
		Scaffold301	PVEN005592	722	TTKYDNVN	YQAQLSTL	77 (Psylloidea)
		Scaffold363	PVEN005138	1171	MHLQASIL	LREKYGFT	83 (Psylloidea)
		Scaffold363	PVEN005140	434	QEDLEKKY	TEKYGETV	60 (Psylloidea)

		Scaffold6471	un	un	TNKYDNFD	QLLDKAKT	88 (Psylloidea)
		Scaffold6471	PVEN013969	189	TKKYDPTG	--------	87 (Psylloidea)
		Scaffold8826	PVEN014347	661	TTKYDVDL	GAKFALKT	70 (Psylloidea)
		Scaffold20457	un	un	TTKYDNVN	--------	97 (Psylloidea)
		Scaffold21288	un	un	TTKYDNVN	EEKYDKTG	76 (Psylloidea)
		Scaffold21840	un	un	TTKYDNVN	--------	70 (Psylloidea)

Table S1. Acypi '4CSP' gene repertoire in comparison to other Hemiptera, genome assembly, and protein identity. *Acypi*: *Acyrthosiphon pisum*, pea aphid (Sternorrhyncha, Aphididae); *Bemta*: *Bemisia tabaci*, silverleaf/tobacco whitefly (Sternorrhyncha, Aleyrodidae); *Halha*: *Halyomorpha halys*, brown marmorated stink bug (Heteroptera, Pentatomidae); *Pacve*: *Pachypsylla venusta*, hackberry petiole gall psyllid (Sternorrhyncha, Aphalaridae). £: three Cys, †: five Cys, º: seven Cys. un: unnoticed transcript. Dashed lines: partial genes.

&: No evidence of Bemta's KY305451 locus, also known as "Bemisia tabaci chemosensory protein 8." Our analysis should not include it (possible contamination).
