# Supplementary material for: Renaming the ‘OS-D/CSP’ Family (Part 2): ‘4-Cysteine Soluble Proteins’ (4CSPs)—Intracellular Functions
Source: Insects. 2026 Sep 8;17(9):940. doi: 10.3390/insects17090940 (PMC13607926; doi:10.3390/insects17090940)
Supplement: Supplementary file 1 [file insects-17-00940-s001.zip › TableS2.pdf]

Locus	mRNA bps	Access Number (R)	Protein aa (kDa)	Access Number (P)	Species	Identity	Tissue	Function
LOC111036095	459	XM_022317999	153 (17.2)	XP_022173691	*Myzus persicae*	Mp10 "CSP4" Ebsp-3	Whole body Asexual adult	Innate Immunity induction
BABH01021709	633	NM_001043604	211 (24.6)	AK368835	*Bombyx mori*	Bommo-CSP10 "BmorCSP10" Bommo-4CSP10	Antennae Legs Fat body Gut Epidermis[f]	Immune response[f]
LOC126581753	2492	XM_050245629	351 (37.7)	XP_050101586	*Anopheles aquasalis*	Mucin-2 Variant X6		Translation initiation factor
LOC109621340	2802	XM_029870982	408 (43.4)	XP_029726842	*Aedes albopictus*	SamkC Variant X9	All body Pupae	Serine-Threonine protein kinase
LOC129745337	1986	XM_055738352	364 (38.8)	XP_055594327	*Uranotaenia lowii*	Mucin C.1 Variant X6	Integument Whole body	Translation initiation factor
LOC115260417	1257	XM_029861367	418 (45.8)	XP_029717227	*Aedes albopictus*	Mucin-2	All body Pupae	Translation initiation factor
LOC126581753	2693	XM_050245628	418 (45.6)	XP_050101585	*Anopheles aquasalis*	Mucin-2 Variant X5		WAS-WASL-interacting protein
LOC118459415	2720	XM_035922756	418 (46.2)	XP_035778649	*Anopheles albimanus*	Extensin-like Variant X5	Whole body Pupae	Cell wall component Defense
LOC118459415	2909	XM_035922754	418 (52.7)	XP_035778647	*Anopheles albimanus*	Extensin-like Variant X7	Whole body Pupae	Leucine-rich repeat Extensin-like
LOC129952650	2022	XM_056065396	361 (39.4)	XP_055921371	*Eupeodes corollae*	Rho GTPase-activating protein X3		Rho GTPase-activator
LOC109621340	3093	XM_029870976	505 (54.4)	XP_029726836	*Aedes albopictus*	Mucin-2 Variant X3	All body Pupae	Nuclear pore protein complex
LOC126564444	1245	XM_050221501	401 (44.3)	XP_050077458	*Aedes maculipalpis*	Mucin-2 Variant X4		Translation initiation factor
LOC118513797	2611	XM_036059988	400 (43.9)	XP_035915881	*Aedes stephensi*	Mucin-2 Variant X3	Whole body	Translation initiation factor

LOC118459415	2461	XM_035922752	547 (56.2)	XP_035778645	*Aedes albimanus*	Mucin-5AC Variant X2	Whole body Pupae	Extensin-like
LOC109621340	3084	XM_029870977	502 (54.8)	XP_029726837	*Aedes albopictus*	Mucin-2 Variant X4	All body Pupae	Nuclear pore protein complex
LOC129744111	2632	XM_055736495	496 (50.1)	XP_055592470	*Uranotaenia lowii*	Mucin-2 Variant X3	Whole body	Nuclear pore protein complex
LOC109621340	3318	XM_029870961	580 (62.3)	XP_029726821	*Aedes albopictus*	Mucin-2 Variant X1	All body Pupae	Nuclear pore protein complex
LOC120904750	2951	XM_040315040	493 (54.4)	XP_040170974	*Aedes arabiensis*	Extensin-like Variant X5		Extensin-like
LOC118459415	2921	XM_035922753	493 (51.4)	XP_035778646	*Aedes albimanus*	Mucin-2 Variant X3	Whole body Pupae	Extensin-like
LOC121596701	3034	XM_041921881	452 (48.7)	XP_041777815	*Anopheles merus*	Mucin-5AC Variant X7	Whole body	Protein transport protein Sec31-like
LOC121596701	3371	XM_041921880	564 (59.6)	XP_041777814	*Anopheles merus*	Mucin-2 Variant X2	Whole body	Mucin5AC-like
LOC121596701	3377	XM_041921874	566 (59.8)	XP_041777808	*Anopheles merus*	Mucin-2 Variant X1	Whole body	Mucin5AC-like
LOC121596701	3022	XM_041921883	448 (47.8)	XP_041777817	*Anopheles merus*	Mucin-2 Variant X5	Whole body	Mucin5AC-like
LOC121596701	3028	XM_041921882	450 (47.9)	XP_041777816	*Anopheles merus*	Mucin-5AC Variant X8	Whole body	Mucin5AC-like
LOC120904750	3137	XM_040315039	555 (60.8)	XP_040170973	*Anopheles arabiensis*	Extensin-like Variant X4	Male	Extensin-like
LOC129745337	2346	XM_055738346	483 (51.0)	XP_055594321	*Uranotaenia lowii*	Mucin-C1 Variant X1	Integument Whole body	Mucin-C1-like
LOC131210824	2638	XM_058204117	386 (42.7)	XP_058060100	*Anopheles bellator*	Mucin-2 Variant X1	Tegument Whole body	Protein UL36
LOC124638351	1860	XM_047175293	292 (32.1)	XP_047031249	*Helicoverpa zea*	PAN1-like	Whole body Pupae	Actin cytoskeleton-regulatory complex
LOC128303246	2787	XM_053040137	472 (51.2)	XP_052896097	*Anopheles moucheti*	Mucin-2 Variant X1		Mucin-like
LOC118513797	2824	XM_036059983	470 (51.2)	XP_035915876	*Anopheles stephensi*	Extensin-like Variant X1	Whole body	Leucine-rich repeat

								Extensin-like
Scaffold00198	878	JH668476	288 (32.1)	KAG6454812	*Manduca sexta*	Hypothetical		
LOC118513797	2774	XM_036059986	454 (49.5)	XP_035915879	*Anopheles stephensi*	Mucin-like Variant X2	Whole body	Mucin-2-like
LOC126374415	3912	XM_050021059	298 (32.7)	XP_049877016	*Pectinophora gossypiella*	YLP motif-protein 1	Whole body Larvae	Nuclear nucleoside kinase
LOC126564444	2774	XM_050221498	554 (59.7)	XP_050077455	*Anopheles macupalpis*	Mucin-2 Variant X1		Mucin-2-like
LOC118459415	3326	XM_035922748	628 (66.0)	XP_035778641	*Anopheles albimanus*	Mucin-2 Variant X1	Whole body Pupae	Mucin-2-like
LOC126564444	1410	XM_050221500	456 (50.0)	XP_050077457	*Anopheles maculipalpis*	Mucin-like Variant X2		Mucin-2-like
Taxon93504	651	LC027715	217 (24.0)	BAV56819	*Ostrinia furnacalis*	"CSP15"	Antennae*	
LOC128271871	1721	XM_053009541	396 (43.6)	XP_052865501	*Anopheles cruzii*	WAS/WASL		Actin skeleton-regulatory complex
LOC128677457	1206	XM_053758324	301 (33.6)	XP_053614299	*Plodia interpunctella*	Formin-1-like	Whole body Larvae	Actin-microtubule association
LOC120904750	3488	XM_040315029	672 (72.6)	XP_040170963	*Anopheles arabiensis*	Extensin-like Variant X1		Extensin-like
LOC111349097	1268	XM_022960083	287 (31.8)	XP_022815851	*Spodoptera litura*	PAN-1	Whole body	Actin skeleton-regulatory complex
LOC5573135	2771	XM_021847563	482 (51.4)	XP_021703255	*Aedes aegypti*	DAN4 Variant X4	Whole body Pupae	Nuclear pore protein complex
LOC126581753	2885	XM_050245624	482 (52.0)	XP_050101581	*Anopheles aquasalis*	Mucin-2 Variant X1		Mucin-like
LOC120904750	3470	XM_040315038	666 (71.5)	XP_040170972	*Anopheles arabiensis*	Extensin-like Variant X9		Leucine-rich protein Extensin-like
LOC120904750	3476	XM_040315037	668 (71.7)	XP_040170971	*Anopheles arabiensis*	Mucin-2 Variant X2	Male	Mucin-like
LOC5573135	3059	XM_021847553	576 (71.5)	XP_021703245	*Aedes aegypti*	NPCP Variant X1	Whole body Pupae	Nuclear pore protein complex
LOC120955660	3533	XM_040376732	667 (71.4)	XP_040232666	*Anopheles*	Mucin-2		Mucin-like

					coluzzii	Variant X1		
LOC106137243	1976	XM_021847553	305 (34.0)	XP_013193496	*Amyelois transitella*	WASP-2	Larvae	Actin skeleton-regulatory complex
LOC6034358	2296	XM_038253956	402 (43.3)	XP_038109884	*Culex quinquefasciatus*	DAN4 Variant X4	Whole body	Cell wall protein
LOC106102595	1190	XM_013282121	294 (32.9)	XP_013137575	*Papilio polites*	GP Ib-like α-chain		Outer surface membrane protein
LOC120427056	2533	XM_052707633	461 (49.5)	XP_052563593	*Culex pipiens pallens*	Mucin-2 Variant X5	Whole body	Mucin-like
M_scaff_1411	820	CAKXAJ010025 123	269 (30.3)	CAH2235359	*Pararge aegeria aegeria*	Jg5928		Major outer envelope protein
LOC108904830	1520	XM_018707509	576 (32.8)	XP_018563025	*Anoplophora glabripennis*	CWA-3 Variant X1	Larvae	Cell wall integrity Stress response
LOC125234523	2861	XM_048140790	287 (32.0)	XP_047996747	*Leguminivora glycinivorella*	RickA-like Variant X2	Whole body Larvae	Arp2/3 complex-activating protein
LOC128271871	2177	XM_053009537	548 (59.0)	XP_052865497	*Anopheles cruzii*	Mucin-2 Variant X1	Tegument	Virus-tegument cross-linker
LOC6034358	2697	XM_038253951	535 (58.9)	XP_038109879	*Culex quinquefasciatus*	Mucin-2 Variant X1	Whole body	Cell wall protein
LOC123011164	1400	XM_044408485	239 (26.3)	XP_044264420	*Tribolium maedens*	Allergen Tha p1 Variant X2	Whole body	IgE binding protein
LOC116772069	1195	XM_032664103	297 (32.9)	XP_032519994	*Danaus plexipus plexipus*	WAS/WASL		Actin skeleton-regulatory complex
LOC129727069	2801	XM_055684473	625 (66.2)	XP_055540448	*Wyeomyia smithii*	Mucin-2 Variant X1	Whole body	Mucin-like
LOC129727069	2580	XM_055684478	553 (58.5)	XP_055540453	*Wyeomyia smithii*	Mucin-2 Variant X3	Whole body	Mucin-like
LOC122855961	1243	XM_044157672	128 (14.5)	XP_044013607	*Aphidius gifuensis*	Allergen Tha p1	Adults	IgE binding protein
LOC129727069	2476	XM_055684480	518 (54.9)	XP_055540455	*Wyeomyia smithii*	Mucin-2 Variant X5	Whole body	Mucin-like
Scaffold_15245	372	CH916367	122 (14.0)	GH22042	*Drosophila grimshawi*	Pherokine-3-PC	Embryo Pupae	Immunity
LOC6532152	607	XM_002092892	239 (13.6)	XP_002092928	*Drosophila*	Ebsp-3/PebIII	Ejaculatory	Sperm storage

					yakuba		bulb/duct	
LOC122859060	559	XM_044162408	128 (14.2)	XP_044018343	*Aphidius gifuensus*	Allergen Tha p1	Adults	IgE binding protein
LOC125060609	487	XM_047665563	121 (14.2)	XP_047521519	*Pieris napi*	Allergen Tha p1		IgE binding protein
LOC123307467	1891	XM_044889794	457 (50.7)	XP_044745729	*Coccinella septempunctata*	Eukaryotic TIF Variant 4 γ		Translation initiation factor
Taxon80765	387	KJ451426	128 (14.4)	AHX71992	*Aphis gossypii*	Acid trehalase "CSP3"	Fat body	Trehalose metabolism
LOC123712760	465	XM_045666008	239 (26.3)	XP_045521964	*Pieris brassicae*	Allergen Tha p1		IgE binding protein
Scaffold00027	846	JANFCV010000 027	282 (31.4)	KAI5642933	*Phthorimaea operculella*	A10/OS-D domain	Adults	
Scaffold133	393	JAANIC010002 949	131 (14.7)	KAG5343447	*Acromyrmex charruanus*	PEB3	Whole body	
LOC124166051	721	XM_046543645	131 (14.5)	XP_046399601	*Ischnura elegans*	Allergen Tha p1 Variant X2		IgE binding protein
PHUM594540	423	XM_002432550	140 (15.5)	XP_002432595	*Pediculus hum. corporis*	Ebsp-3/PebIII	Larvae	
LOC123010695	476	XM_044407769	124 (14.4)	XP_044263704	*Tribolium maedens*	Allergen Tha p1	Whole body Pupae	IgE binding protein
Chromosome 2R	339	CP012524	113 (13.1)	ALC42649	*Drosophila busckii*	PebIII	Whole body Larva	
Dmel_CG9358	363	NM_001299941	121 (13.4)	NP_001286870	*Drosophila melanogaster*	Pherokine-3 Variant B	Embryonic gonads	Immunity
Scaffold_15245	366	CH916367	122 (13.8)	EDW02527	*Drosophila grimshawi*	GH22042/OS-D		
LOC107225040	553	XM_015665345	127 (14.1)	XP_015520831	*Neodiprion lecontei*	Allergen Tha p1 Variant X1	Thorax Abdomen	IgE binding protein
LOC124186858	574	XM_046578909	127 (14.2)	XP_046434865	*Neodiprion fabricii*	Allergen Tha p1	Thorax Abdomen	IgE binding protein
LOC124166051	708	XM_046543644	131 (14.2)	XP_046399600	*Ischnura elegans*	Allergen Tha p1 Variant X1		IgE binding protein
LOC124223445	658	XM_046635425	127 (14.2)	XP_046491381	*Neodiprion pinetum*	Allergen Tha p1 Variant X2	Thorax Abdomen	IgE binding protein
LOC124308780	659	XM_046771826	127 (14.2)	XP_046627782	*Neodiprion*	Allergen Tha p1	Thorax	IgE binding

					virginianus	Variant X2	Abdomen	protein
LOC110991536	755	XM_022256909	121 (14.2)	XP_022112601	*Pieris rapae*	Allergen Tha p1		IgE binding protein
Contig_79	360	LYFD01000069	120 (13.8)	OIB81003	*Acinetobacter baumannii*	A10/OS-D	Prokaryotic cell	
Contig_92	261	LYGG01000091	87 (9.8)	OIC85870	*Acinetobacter baumannii*	A10/OS-D	Prokaryotic cell	

Table S2. Sequences producing significant alignments with Mp10 (query length 153 amino acids; e-value 2e-107 to 3e-31). The sequences are listed from highest identity % protein to query to lowest identity percentage protein (NCBI BLAST, blastp, nr database). OIB81003 and OIC85870 are from microbial protein blast [14].

* The asterisk denotes that an antennal clone was used to obtain the sequence. There was no tissue-distribution research done. *f* Results of the real-time PCR tissue distribution analysis test [2]. Lack of information leaves a void under Tissue and Function. We renamed BmorCSP10 to "4-Cysteine Soluble Protein10" ("4CSP10") instead of "Chemosensory Protein" ("CSP10").
