# Supplementary material for: Renaming the ‘OS-D/CSP’ Family (Part 2): ‘4-Cysteine Soluble Proteins’ (4CSPs)—Intracellular Functions
Source: Insects. 2026 Sep 8;17(9):940. doi: 10.3390/insects17090940 (PMC13607926; doi:10.3390/insects17090940)
Supplement: Supplementary file 1 [file insects-17-00940-s001.zip › TableS3.pdf]

Name	Nb aa	MW (kDa)	Access Number (P)	Organism Species	Identity	Tissue Chr Gene locus	Function
AaegCWPDAN4X4	482	51.4	XP_021703255	*Aedes aegypti*	DAN4 isoform X4	Whole pupae, tegument Chr2	Cell wall protein
AaquTIF2	351	37.7	XP_050101586	*An. aquasalis*	Translation initiation factor IF-2-like isoform X3	Chr2	"OS-D-like" Translation initiator
AaquWAS/WASL	418	45.4	XP_050101585	*Anopheles aquasalis*	WAS/WASL-interacting protein family member 1-like isoform X2	Chr2 LOC126581753	"OS-D-like" Mucin-2
AEalbSamkC	408	44.3	XP_029726842	*Ae. albopictus*	SamkC isoform X9	All body Chr2	Ser/Thr-protein kinase
AglaCWPX3	243	26.7	XP_018563027	*Anoplophora glabripennis*	CWP3-like isoform X3	Larvae LOC108904830	"OS-D" Integrity of cell wall Stress response
ANalbLRRX4	489	52.6	XP_035778647	*Anopheles albimanus*	LRR-like protein 5 isoform X4	Adult female, whole body Chr2	"OS-D" Leucine-rich repeat extensin
AcruMucin2	548	58.6	XP_052865497	*An. cruzii*	Mucin2-like isoform 1	Tegument Chr3	Mucin
AcruWAS/WASL	396	43.6	XP_052865501	*An. cruzii*	WAS/WASL isoform X4 UL36	Tegument Chr3	WAS/WASL-interacting protein
AmerMucin2	448	47.8	XP_041777817	*An. merus*	Mucin-2-like isoform X5	Adults, whole body Chr3R	Mucin
AmerMucin5AC	452	48.7	XP_041777815	*An. merus*	Sec-31-like isoform X3	Adults, whole body	Protein transport protein
AMS2711750	96	9.6	KPK83846	*Bacteroides* sp. SM23_62_1 Bacteria Bacteroidia Bacteroidales	Hypothetical protein AMS27_11750	Sediment metagenome	Hypothetical
AplanCSP	325	37.3	CAB3231652	*Arctia plantaginis*	Unnamed	LOCUS4521	"OS-D"-like
CAUAvd	136	16.1	DAK32723	*Heunggongvirae* Caudoviricetes, Viruses	*ctUaT18 Avd-like	Human metagenome	DGR-protein
CparMraZ	164	18.7	HHE32374	*Chlorobaculum parvum* Bacteria Chlorobiia Chlorobiales	MraZ	Hydrothermal vent metagenome	Transcriptional regulator
CsecThap1	126	14.4	XP_023715540	*Cryptotermes secundus*	Tha p 1	Workers, whole body	Allergen
CUPHUDBP	152	15.8	WP_035823962	*Cupriavidus sp.* SK-4 Bacteria β-Proteobacteria; Burkholderiales	DNA-binding protein HU family		Histone-like DBP Chromosome condensation
EbalCSP3	213	24.2	QIS77190	*Episyrphus balteatus*	"CSP"		"OS-D"-like

EbalCSP4	321	35.3	QIS77191	*E. balteatus*	"CSP"		"OS-D"-like
EbalDDBG0285119 X1	371	41.1	XP_055839111	*E. balteatus*	DDB_ G0285119 isoform X1	Chr1	"OS-D-like"
EcorRho	361	39.4	XP_055921371	*Eupeodes corollae*	Rho17 isoform X3	Chr3	Rho GTPase-activator
HhatDBPXRE	154	18.0	EFC99453	*Hungatella hathewayi* DSM 13479 Bacteria, Clostridia, Eubacteriales	DNA-binding helix-turn-helix protein	Cytoplasm, CLOSTHATH_ 02328	Transcriptional regulator XRE-family
IpodCSP	263	30.7	CAH2042437	*Iphiclides podalirius*	Unnamed	Chr14 IPOD504_ LOCUS3820	"OS-D"-like
LborTetR/AcrR	170	19.2	WP_011671486 WP_061220709	*Leptospira borgpetersenii* Bacteria Spirochaetia Leptospirales	TetR/AcrR		Transcriptional regulator Regulation of DNA-templated transcription
LlonGTA1	475	52.6	WP_058530090	*Legionella londiniensis* Bacteria g-Proteobacteria Legionellales	Mannose-1-P-guanylyl transferase/ mannose-6-phosphate isomerase		Mannose Glycosyl transferase family A
LlonGTA2	486	54.0	KTD19891	*L. londiniensis* Bacteria g-Proteobacteria Legionellales	Phospho mannose isomerase GDP mannose pyro-phosphorylase	Llon_ctg033	P-mannose Glycosyl transferase family A
OvarDBPXRE	143	16.3	BAL01528	*Oscillibacter valericigenes* Sjm18-20, Bacteria, Clostridia, Eubacteriales	DNA-binding protein	OBV_43290	Transcriptional regulator XRE-family Nucleotide binding
PaegJg6675	217	25.0	CAH2266975	*Pararge aegeria aeg.*	Jg6675	PAEG_Locus 25571	"OS-D"-like
PlutMraZ	153	17.3	KZK74918 WP_303680859 WP_011358831	*Pelodictyon luteolum* (*Chlorobium luteolum*) Bacteria Chlorobiia Chlorobiales	Division/cell wall cluster transcriptional repressor MraZ	Lake water metagenome	Transcriptional regulator
PpyrMucin5AC	338	37.7	XP_031351203	*Photinus pyralis*	Mucin-5AC -like	Adult male, whole body LOC116176644	"OS-D-like" Mucin
SFRURICE011779	265	30.8	KAF9818733	*Spodoptera frugiperda*	A10/OS-D	Adult males, whole body	"OS-D-like"
SfulWP223199665	67	15.5	WP_223199665	*Solihabitans fulvus* Bacteria, Actinomycetes,	A10/OS-D		"OS-D-like"

				Pseudonocardiales			
TechAcrR	193	20.7	SEF50864	*Thermonospora echinospora*<br>Bacteria, Actinomycetota, Actinomycetes, Streptosporangiales	**AcrR family DNA-binding protein	SAMN0448971 2_101243	DNA-binding transcriptional regulator
RtorDBPXRE1	138	15.6	WP_044998036	*Ruminococcus torques*<br>Bacteria Clostridia, Eubacteriales	Helix-turn-helix Transcriptional regulator		Transcriptional regulator XRE-family Nucleotide binding
RtorDBPXRE2	146	16.7	EDK23754	*Ruminococcus torques*<br>Bacteria, Clostridia, Eubacteriales	DNA-binding helix-turn-helix protein	Contig63	Transcriptional regulator XRE-family Nucleotide binding
WP016226835	135	15.7	EOS77856<br>WP_016226835	*Lachnospiraceae bacterium*<br>10-1<br>Bacteria, Clostridia, Eubacteriales	Hypothetical	C819_00471	Hypothetical
WP020776583	163	18.9	WP_020776583	*Leptospira meyeri*<br>Bacteria Spirochaetia Leptospirales	Hypothetical		Hypothetical
WP004787694	163	19.0	WP_004787694	*L. meyeri*<br>Bacteria Spirochaetia Leptospirales	Hypothetical		Hypothetical
WsmiSamkC	520	56.2	XP_055540454	*Wyeomyia smithii*	Serine/threonine -protein kinase SamkC isoform X4	Adult male, whole body Chr3 LOC129727069	Ser/Thr kinase SamkC

Table S3. Sequences producing significant PAUP-Tree between 4CSP and intracellular proteins. 4CSP-related intracellular proteins are CWP, LRR, Rho, SamkC, Sec31, TIF, WAS-WASL, and DBPs (transcriptional regulators of the XRE-family).
*Viral Accessory variability determinant (Avd) proteins, such as ctUaT18, are a subset of Diversity Generating Retroelements (DGR) genetic cassettes that mutate target genes only in a specific way to produce hypervariable proteins.
**Bacterial AcrR is a single-component repressor of the antibiotic resistance and lipid transport genes.
Chr: Chromosome.
