# Supplementary material for: Renaming the ‘OS-D/CSP’ Family (Part 2): ‘4-Cysteine Soluble Proteins’ (4CSPs)—Intracellular Functions
Source: Insects. 2026 Sep 8;17(9):940. doi: 10.3390/insects17090940 (PMC13607926; doi:10.3390/insects17090940)
Supplement: Supplementary file 1 [file insects-17-00940-s001.zip › TableS4.pdf]

**Name**	**Nb aa**	**MW (kDa)**	**Access Number (P)**	**Organism Species**	**Identity**	**Tissue Chr Gene locus**	**Function**
AalbRBPFOX1	671	67.0	XP_062709901	*Aedes albopictus*	RNA-binding protein fox-1 homolog 1 isoform X8	Adult male, whole body<br>Chr2<br>LOC109419707	Splicing regulation (via GCAUG)
AdarRBP	405	43.7	ETN67466<br>XP_049529535	*Anopheles darlingi*	RNA-binding protein<br>Cleavage stimulation factor	Chr2	RNA recognition motif<br>Transcription termination
ARS-RBPYhbY	97	10.8	RXK34257<br>WP_032114384	*Arsenophonus*<br>Multispecies<br>Bacteria<br>γ-proteobacteria<br>Enterobacterales	RNA-binding protein YhbY	Cytoplasm<br>Endosymbiont of. *B. tabaci*<br>Bacteriocyte	RNA binding<br>Preribosome binding<br>Ribosome assembly*
AsinRBP	64	7.2	KFB40836	*An. sinensis*	RNA-binding protein	ZHAS_ 0008365	RNA binding
BmorRBP1	142	16.7	XP_012547769	*Bombyx mori*	RNA-binding protein-1 isoform X2	Chr15<br>Gene Rbp1<br>733053	RNA binding
BtabRBP15	251	28.0	UYF10700<br>XP_018914255	*Bemisia tabaci*	RNA-binding protein 15	Adult, whole body	RNA motif-binding
MturRBP	80	8.4	WP_086153840	*Mycobacterium Tuberculosis*<br>Bacteria<br>Actinomycetes<br>Mycobacteriales	RNA-binding protein		Nucleotide binding
NOC-RBP	80	8.3	MBO08556625<br>WP_040708791<br>WP_067514134	*Nocardia*<br>Multispecies<br>Bacteria<br>Actinomycetes<br>Mycobacteriales	RNA-binding protein	Termitarium metagenome	Nucleotide binding
TcasRBPFOX1	178	18.8	KYB27196<br>XP_008193531<br>XP_008193529	*Tribolium castaneum*	RNA-binding protein fox-1 homolog 3-like	LOC655682	RNA motif-<br>Nucleotide binding

Table S4. Sequences of RNA-binding proteins (RBPs) used in PAUP-tree for molecular comparisons with 4CSP and other intracellular proteins. 4CSP-related intracellular proteins are: CWP, Rho, SamkC, Sec31, TIF, WAS-WASL, and DBPs.

* Associated with the pre-50S ribosome subunit in ribosome assembly, required for optimal growth but not for cell viability.
